# National Burden of Breast Cancer in Saudi Arabia, 1990–2023, With Forecasts to 2050: A Systematic Analysis for the Global Burden of Disease Study 2023

**DOI:** 10.65416/ehealthsci.2026.117757

**Published:** 2026-04-05

**Authors:** Yazan J. Alalwani, Shimah Maibed Alsalhi, Nawaf Salem Baradhwan, Rahaf Muqbil Alsubaie, Norah Abdullah Alhweish, Rawan Abdullah A. Jarah, Lujain Abdulrahman Alsarhan, Reem Mohammed A. Albarrak, Zahra Shafiq Yousef Almatar, Njood Mohammed Faleh Alhajri, Rahaf Abdullah Ali Alshahrani, Eiman Mohammed AlShammari, Ahmed Y. Azzam

**Affiliations:** a College of Medicine, Imam Abdulrahman Bin Faisal University, Khobar, Saudi Arabia; b College of Medicine, King Saud University, Riyadh, Saudi Arabia; c College of Medicine, Umm Al-Qura University, Makkah, Saudi Arabia; d College of Medicine, Princess Nourah Bint Abdulrahman University, Riyadh, Saudi Arabia; e Faculty of Medicine, University of Tabuk, Tabuk, Saudi Arabia; f College of Medicine, Qassim University, Buraydah, Saudi Arabia; g Department of General Surgery, King Fahad Hospital of the University, College of Medicine, Imam Abdulrahman Bin Faisal University, Khobar, Saudi Arabia; h Division of Global Health and Public Health, School of Nursing, Midwifery and Public Health, University of Suffolk, Ipswich, United Kingdom; i Department of Neuroradiology, West Virginia University, Morgantown, West Virginia, United States

**Keywords:** Breast Cancer, Breast, Breast Oncology, Saudi Arabia, Health Policy, Cancer Epidemiology, Breast Malignancies, Global Burden of Disease, Epidemiology, General Surgery, Public Health, Obstetrics & Gynecology, Oncology

## Abstract

**Introduction:**

Breast cancer represents the most common malignancy among Saudi Arabian women; however, structured population-level burden assessments integrating advanced epidemiological methods remain limited. We quantified the growing burden of breast cancer in Saudi Arabia from 1990 to 2023 and projected trends to 2050.

**Methods:**

Using Global Burden of Disease 2023 estimates, we analyzed incidence, mortality, and disability-adjusted life-years (DALYs) through joinpoint regression, age-period-cohort modeling, and Das Gupta decomposition. Forecasts utilized Bayesian model averaging across four statistical models. Healthcare system performance was assessed via mortality-to-incidence ratio trajectories against socio-demographic index benchmarks.

**Results:**

Incident cases increased from 454 (95% Uncertainty Interval [UI]: 322–630) in 1990 to 4,168 (3,009–5,990) in 2023, representing an 818% increase. Age-standardized incidence rose from 6.47 to 19.58 per 100,000 (+203%). Deaths increased from 243 to 1,197 (+393%), while DALYs rose from 8,524 to 43,561 (+410%). The mortality-to-incidence ratio improved from 0.535 to 0.362 (−32%), indicating enhanced survival. Age effects explained 77.7% of variance in incidence patterns, with period effects contributing 20.6%. Decomposition attributed 44% of the incidence increase to population growth, 48% to epidemiological change, and 7% to aging. Performance frontier analysis identified 659 preventable deaths annually (57% of total) if Saudi Arabia achieved benchmark efficiency. Ensemble forecasts project stabilization at 19.3 per 100,000 by 2050.

**Conclusions:**

The breast cancer burden in Saudi Arabia has increased significantly, driven primarily by true epidemiological change and population growth. Despite improving survival, significant preventable mortality persists, necessitating enhanced screening and treatment optimization.

## 1. INTRODUCTION

Breast cancer constitutes the most frequently diagnosed malignancy among women globally, accounting for approximately 2.3 million new cases and 685,000 deaths annually. Breast cancer burden demonstrates marked geographic heterogeneity, with age-standardized incidence rates ranging from below 30 per 100,000 in parts of sub-Saharan Africa to exceeding 90 per 100,000 in Western Europe and North America. This variation reflects complex interactions between genetic susceptibility, reproductive factors, metabolic risk factors, healthcare access, and screening penetration [1-3].

Saudi Arabia has undergone rapid socioeconomic transformation over the past three decades, with the Socio-demographic Index (SDI) increasing from 0.62 in 1990 to 0.83 in 2023. This transition has been accompanied by major shifts in lifestyle factors associated with breast cancer risk, including increasing obesity prevalence, declining physical activity, later age at first birth, and reduced breastfeeding duration. Concurrently, healthcare infrastructure has expanded considerably, with the establishment of comprehensive cancer centers and the introduction of national screening initiatives [4-7].

Previous investigations of breast cancer epidemiology in Saudi Arabia have been limited by reliance on hospital-based registries, restricted geographic coverage, abbreviated temporal windows or limited utilization of statistical and computational approaches that can be further integrated for better quantification of breast cancer in Saudi Arabia [6,8]. Population-based estimates including uncertainty quantification, advanced decomposition methods, and projection modeling remain sparse. Understanding the precise magnitude of burden change, the relative contributions of demographic versus epidemiological drivers, and the trajectory of healthcare system performance is essential for evidence-based resource allocation and policy formulation [9].

The Global Burden of Disease (GBD) Study 2023 provides standardized estimates enabling comprehensive assessment of disease burden across locations and time periods [10,11]. The present analysis utilizes these data to characterize the growing burden of breast cancer among Saudi Arabian population from 1990 to 2023, decompose temporal changes into constituent components, evaluate healthcare system efficiency against international benchmarks, and project burden trajectories to 2050. These findings aim to inform national cancer control priorities and identify opportunities for mortality reduction.

## 2. MATERIALS AND METHODS

### Data Source and Study Population:

This systematic analysis utilized estimates from the GBD Study 2023, coordinated by the Institute For Health Metrics and Evaluation (IHME). The GBD utilizes standardized analytical frameworks to synthesize data from vital registration systems, cancer registries, verbal autopsy studies, and health surveys to generate internally consistent estimates of incidence, prevalence, mortality, years of life lost (YLLs), years lived with disability (YLDs), and disability-adjusted life-years (DALYs) for 371 diseases and injuries across 204 countries and territories from 1990 to 2023.

Breast cancer was defined according to International Classification of Diseases, Tenth Revision code (ICD-10) C50, including all malignant neoplasms of breast tissue. The study population included all residents of Saudi Arabia population regardless of if they are Saudi citizens or not, with sex-specific analyses focusing on females given that 96.6% of incident cases occur in women. Age-specific analyses included 18 five-year age groups from 15–19 to 85–89 years, plus a terminal category of 90–94 years.

The GBD study operates under a waiver of informed consent approved by the University of Washington Institutional Review Board (IRB), given exclusive use of de-identified, aggregated secondary data. No additional ethical approval was required for this analysis.

Data for this study were obtained from the GBD 2023 Study through the GBD Results Tool, an interactive data visualization and download platform maintained by the IHME at the University of Washington (https://vizhub.healthdata.org/gbd-results/). We extracted age-specific and age-standardized estimates for breast cancer incidence, mortality, prevalence, YLDs, YLLs, and DALYs for Saudi Arabia from 1990 to 2023.

### Burden Estimation and Uncertainty Quantification:

Incidence and mortality estimates were generated using DisMod-MR 2.1, a Bayesian meta-regression tool that synthesizes heterogeneous data sources while enforcing epidemiological consistency constraints [12]. This compartmental model simultaneously estimates incidence, prevalence, remission, and cause-specific mortality while maintaining internal consistency through differential equations governing transitions between disease states. The model integrates Gaussian process priors for age patterns, hierarchical random effects for geographic variation, and study-level covariates to adjust for systematic bias in data sources. Uncertainty was propagated through 1,000 posterior draws from the joint distribution of all parameters.

DALYs were calculated as the sum of YLLs and YLDs following standard GBD methodology. YLLs were computed by multiplying deaths in each age-sex group by the residual life expectancy from the GBD standard reference life table, which uses the lowest observed mortality rates across all age groups from countries with populations exceeding five million. YLDs were calculated as prevalent cases multiplied by disability weights derived from population-based surveys conducted in nine countries using paired comparison methods. Disability weights for breast cancer ranged from 0.049 (95% confidence interval [CI]: 0.031–0.072) for the controlled remission phase to 0.540 (0.377–0.687) for the terminal phase, with intermediate values of 0.288 (0.193–0.399) for primary treatment and 0.451 (0.307–0.600) for metastatic disease.

Age-standardization utilized the GBD world population standard to enable valid comparisons across time periods and populations with differing age structures. All estimates are reported with 95% uncertainty intervals (UI) derived from the 2.5th and 97.5th percentiles of posterior distributions [13].

### Temporal Trend Analysis:

Temporal trends were assessed using joinpoint regression, which identifies statistically significant changes in trend direction through permutation testing. The algorithm fits piecewise log-linear segments using weighted least squares, with joinpoints representing calendar years where the annual percent change (APC) shifts significantly. The grid search method evaluated all possible joinpoint configurations, with significance determined at α=0.05 using 4,499 Monte Carlo permutations under the null hypothesis of no joinpoint [14-16]. Up to three joinpoints were permitted based on Bayesian Information Criterion (BIC) optimization. Average annual percent change (AAPC) summarized overall trends as the weighted geometric mean of segment-specific APCs, with weights proportional to segment length [17,18].

Trend robustness was evaluated through complementary non-parametric methods. The Mann-Kendall test assessed monotonic trend presence without distributional assumptions, while Sen’s slope estimator provided a robust measure of trend magnitude resistant to outliers [19]. Structural break detection employed the Chow test for known break points and Pettitt’s non-parametric change-point analysis for unknown break points. Autocorrelation was assessed via Durbin-Watson statistics, with Newey-West heteroskedasticity and autocorrelation-consistent standard errors applied when serial correlation was detected [20].

### Age-Period-Cohort Modeling:

Age-period-cohort analysis was conducted to disentangle the independent effects of age at diagnosis, calendar period, and birth cohort on incidence patterns. This approach addresses confounding between temporal dimensions, as observed rate changes may reflect biological aging, period-specific exposures affecting all ages simultaneously, or cohort-specific early-life exposures persisting across the lifespan.

Given the identification problem arising from the linear dependency among age, period, and cohort (birth cohort = calendar period − age), we used Bayesian age-period-cohort modeling with intrinsic conditional autoregressive priors [21]. This approach imposes smoothness constraints through second-order random walk priors on age, period, and cohort effects, with precision parameters estimated from the data. The model specification followed:

where μ_ij_ represents expected cases in age group i and period j, α is the intercept, and the Greek letters represent age, period, and cohort effects respectively. Model estimation used integrated nested Laplace approximation (INLA) for computational efficiency.

Variance decomposition quantified the relative contributions of age, period, and cohort effects to overall deviance using sequential analysis of deviance. Rate ratios with 95% credible intervals were calculated relative to reference categories at the median values of each dimension. Lexis diagrams provided visualization of incidence patterns across the age-period plane, with diagonal bands representing birth cohorts [22-24].

### Decomposition of Burden Changes:

Changes in absolute burden between time periods were decomposed into contributions from population growth, population aging, and epidemiological (rate) change using Das Gupta’s stepwise standardization method with three-factor interaction terms [25,26]. This approach sequentially substitutes population size, age structure, and age-specific rates between comparison periods using the formula:

where N represents burden counts, P is total population, A is age structure (proportion in each age group), and R represents age-specific rates. The method allocates interaction terms proportionally to main effects.

Robustness was assessed through Shapley value decomposition, which provides order-invariant attribution by averaging contributions across all 3! = 6 possible substitution sequences. This approach ensures that the sum of attributed components exactly equals total change regardless of decomposition order. Kitagawa’s two-factor decomposition with Monte Carlo uncertainty propagation (n=500 iterations) separated rate and composition effects while integrating GBD UIs through parametric bootstrapping [27]. Oaxaca-Blinder decomposition using period one as reference provided supplementary estimates, distinguishing explained (compositional) from unexplained (coefficient) differences [28,29].

### Age-Specific Analysis:

Age-specific rates for 2023 were smoothed using log-polynomial regression to ensure positive fitted values while capturing the characteristic age-incidence curve. Model selection utilized Akaike Information Criterion with finite sample correction (AICc), with polynomial degree 4 selected based on minimizing AICc while avoiding overfitting. Goodness-of-fit was assessed through adjusted R^2^ and residual diagnostics. First derivatives of fitted functions identified the age at peak incidence acceleration, representing the inflection point in the age-specific rate curve.

Rate ratios comparing 2023 to 1990 reference rates were calculated for each age stratum with 95% uncertainty intervals derived from the delta method applied to log-transformed rates. Age-specific APCs were estimated via log-linear regression across the 34-year observation period, with Bonferroni correction for multiple comparisons across age groups.

### Forecasting Methodology:

Burden projections to 2050 was performed through an ensemble approach combining four complementary statistical models to address structural uncertainty in long-range forecasting. The Lee-Carter model, originally developed for demographic mortality forecasting, was adapted for cancer incidence by decomposing log-rates into age-specific constants, age-specific sensitivity parameters, and a time-varying index following a random walk with drift [30,31]. The Nordpred power-5 link model estimated period and cohort effects with attenuation of recent trends to avoid unrealistic extrapolation [32]. Age-period-cohort projection extended observed period and cohort trends using autoregressive integrated moving average processes. Bayesian structural time series modeling incorporated local linear trends with seasonality components and regression on covariates including SDI projections [33,34].

Model combination utilized Bayesian model averaging (BMA) with weights proportional to exponentiated negative BIC values:

This approach accounts for structural uncertainty by weighting models according to their approximate posterior probabilities. Prediction intervals included both within-model parameter uncertainty (from posterior distributions) and between-model structural uncertainty (from weighted model averaging) [35-37].

Variance decomposition partitioned total forecast uncertainty into four components using the law of total variance: model selection uncertainty (between-model variance), parameter estimation uncertainty (within-model variance), demographic projection uncertainty (propagated from the united nations population projections), and irreducible stochastic variation [38-40]. Scenario analyses investigated optimistic (accelerated improvement at 1.5× observed rate), pessimistic (adverse trend acceleration at 0.5× improvement), and Sustainable Development Goal target (25% mortality reduction by 2030, 50% by 2050) trajectories.

### Healthcare System Performance Assessment:

Healthcare system efficiency was evaluated through mortality-to-incidence ratio (MIR) analysis, a validated proxy for case fatality that reflects combined effects of stage at diagnosis, treatment quality, and follow-up care. The MIR has demonstrated strong correlation with population-based survival estimates (r > 0.9) and provides a comparable metric across settings lacking survival data [41,42]. The MIR trajectory for Saudi Arabia from 1990 to 2023 was benchmarked against the global efficiency frontier, defined as the lowest MIR achieved at each level of SDI across all 204 GBD locations using stochastic frontier analysis. SDI is a composite indicator ranging from 0 to 1, calculated as the geometric mean of lag-distributed income per capita, average educational attainment in the population over age 15, and total fertility rate under 25.

Performance frontier analysis quantified the gap between observed MIR and the frontier benchmark at Saudi Arabia’s current SDI level using the formula:

Comparator countries were selected to represent relevant peer groups: Australia and Republic of Korea (high-income Organisation for Economic Co-operation and Development [OECD] with frontier-level performance), United Arab Emirates (Gulf Cooperation Council regional peer), and Egypt (Middle East and North Africa developing economy).

### Counterfactual Policy Scenario Modeling:

G-computation, a parametric generalization of standardization, was employed to estimate mortality under alternative intervention scenarios. This approach fits outcome models conditional on exposure and confounders, then predicts counterfactual outcomes under hypothetical intervention levels. The status quo scenario extrapolated current trends assuming no policy change [43]. Intervention scenarios modeled mortality reductions based on published effectiveness estimates: enhanced early screening (20% mortality reduction based on randomized trial meta-analyses), improved treatment access (25% reduction based on stage-shift modeling), risk factor modification (12% reduction based on population attributable fractions [PAF]), and combined optimal implementation (47% reduction assuming multiplicative independent effects). Cumulative lives saved through 2040 were estimated by integrating the difference between status quo and intervention trajectories.

### Risk Factor Attribution:

PAF for breast cancer mortality and DALYs were calculated using the comparative risk assessment framework from GBD 2023. This approach estimates the proportional reduction in burden that would occur if exposure to each risk factor were reduced to a theoretical minimum risk level. Relative risks were derived from meta-regression using MR-BRT (meta-regression—Bayesian, regularized, trimmed) methodology, which applies 10% trimming of outlier studies and integrates between-study heterogeneity. Burden of proof risk functions quantified evidence strength by computing the risk function consistent with the 5th percentile of the relative risk distribution [44].

Attributable burden was calculated for established breast cancer risk factors with sufficient evidence: high body-mass index (BMI), (BMI ≥25 kg/m^2^), alcohol consumption, low physical activity, high fasting plasma glucose, and diet low in fiber. Exposure distributions were derived from nationally representative surveys and modeling.

### Software and Reproducibility:

All analyses were conducted in R version 4.3.2 (R Foundation for Statistical Computing, Vienna, Austria) and Python version 3.11. Joinpoint regression utilized the Joinpoint Regression Program version 5.0.2 (National Cancer Institute, Bethesda, MD, USA) [45]. Bayesian models utilized Stan version 2.32 via the brms interface. Age-period-cohort modeling used the INLA package. Figures were generated using ggplot2 version 3.4.4. This study followed the Guidelines for Accurate and Transparent Health Estimates Reporting (GATHER) recommendations [46].

## 3. RESULTS

### Baseline Characteristics and Burden Overview:

Table 1 presents baseline characteristics and burden metrics for 1990 and 2023. Incident breast cancer cases in Saudi Arabia increased from 454 (95% UI: 322–630) in 1990 to 4,168 (3,009–5,990) in 2023, representing an absolute increase of 3,714 cases and a relative increase of 818.1%. The age-standardized incidence rate rose from 6.47 (4.56–8.94) to 19.58 (14.63–25.80) per 100,000 population, a 202.6% increase. Among females, the age-standardized rate increased from 15.28 (10.70–21.31) to 49.36 (36.19–66.12) per 100,000, representing a 223.0% increase over the study period. Deaths from breast cancer increased from 243 (172–334) in 1990 to 1,197 (901–1,667) in 2023, a 392.6% increase. The age-standardized mortality rate rose from 4.12 (2.86–5.62) to 7.57 (5.74–10.56) per 100,000. Total DALYs increased from 8,524 (6,144–12,000) to 43,561 (33,174–60,890), a 410.9% increase. The MIR improved from 0.535 in 1990 to 0.362 in 2023, indicating a 32.3% improvement in survival probability. Annual time series data for both sexes combined and by sex are presented in Supplementary Table 1 and Supplementary Table 2, respectively, while YLLs, YLDs, and prevalence trajectories are detailed in Supplementary Table 3.

### Temporal Trend Analysis:

Table 2 summarizes temporal trend analyses. Joinpoint regression identified significant inflection points in breast cancer trajectories (Supplementary Figure 1). For incidence, a joinpoint in 2007 delineated two peculiar phases: rapid acceleration from 1990 to 2007 (APC +4.5%; 95% CI: 4.1–4.9) followed by deceleration from 2007 to 2023 (APC +1.9%; 1.4–2.4). Mortality showed a joinpoint in 2005, with modest increase from 1980 to 2005 (APC +0.7%; 0.3–1.2) accelerating thereafter (APC +1.1%; 0.5–1.7).

**Figure 1:**
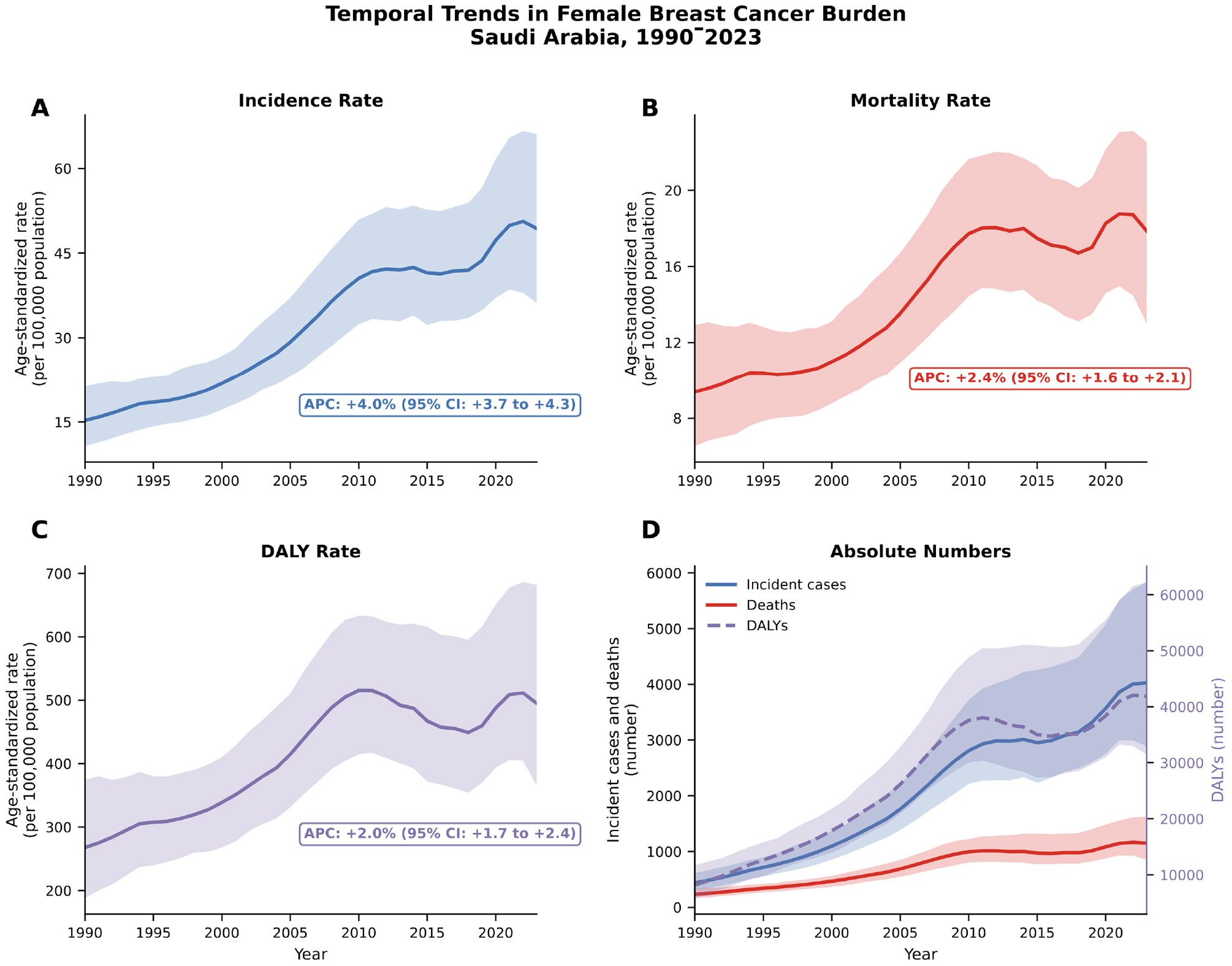
Temporal Trends In Female Breast Cancer Burden In Saudi Arabia Between 1990–2023.

Period-specific analysis revealed significant acceleration during 2000–2010, with incidence APC reaching 6.64% (6.39–6.88) and mortality APC 5.05% (4.77–5.34), followed by stabilization during 2010–2023 when incidence APC declined to 1.17% (0.70–1.65) and mortality APC became non-significant at −0.14% (−0.59 to 0.32). Figure 1 illustrates temporal trajectories for incidence, mortality, and DALYs from 1990 to 2023. Detailed statistical parameters including Mann-Kendall tests, Sen’s slopes, and model diagnostics are provided in Supplementary Table 4.

### Decomposition of Burden Changes:

Table 3 presents decomposition analyses of burden changes. Das Gupta stepwise decomposition attributed the 1990–2023 increase in incident cases (n=3,714) to population growth (44.0%), epidemiological rate change (48.0%), population aging (6.6%), and interaction terms (1.4%). For mortality, population growth contributed 60.3%, rate change 33.4% and aging 6.0%. The contribution of aging increased significantly in the 2010–2023 period, reaching 34.4% for incidence and 77.7% for mortality.

**Figure 2:**
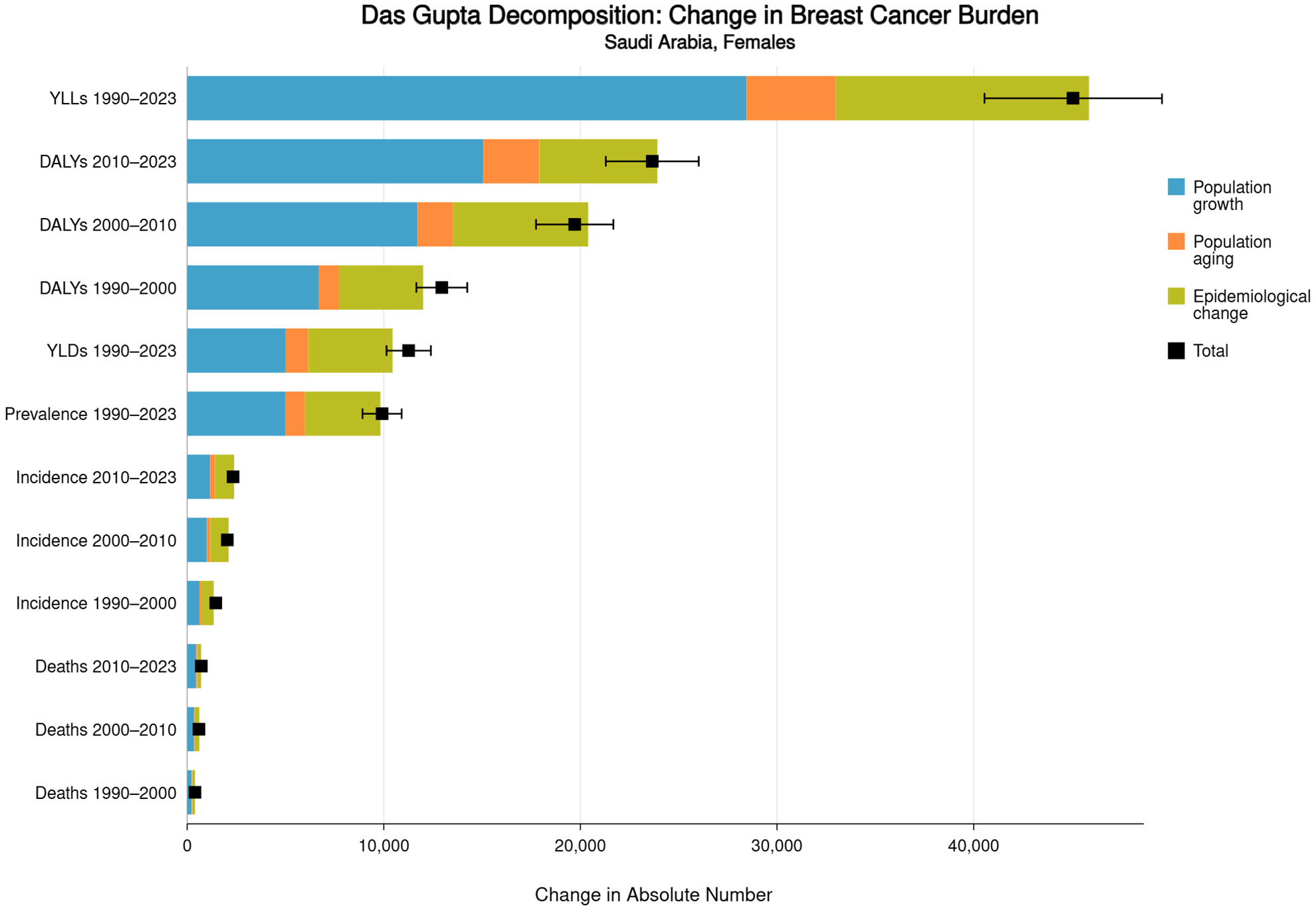
Das Gupta Decomposition of Burden Changes By Time Period.

Shapley value decomposition confirmed these patterns with order-invariant attribution. During 2010–2023, epidemiological rate change for mortality became negative (−105.0%), indicating that underlying mortality rates actually declined when controlling for demographic factors, offset by population growth and aging effects. Figure 2 visualizes the decomposition across three time periods, demonstrating the shifting relative importance of demographic versus epidemiological drivers.

### Forecasting Projections:

Table 4 presents forecasting projections to 2050. Under reference assumptions, the age-standardized incidence rate is projected to stabilize at 19.29 (95% UI: 16.67–21.90) per 100,000 by 2050. Mortality is projected at 7.41 (6.44–8.38) per 100,000, with DALYs at 191.29 (165.58–217.00) per 100,000. Absolute incident cases are projected to reach 5,360 (1,930–8,790) by 2050, with 1,418 deaths (510–2,325) and 52,257 DALYs (18,813–85,702).

**Figure 3:**
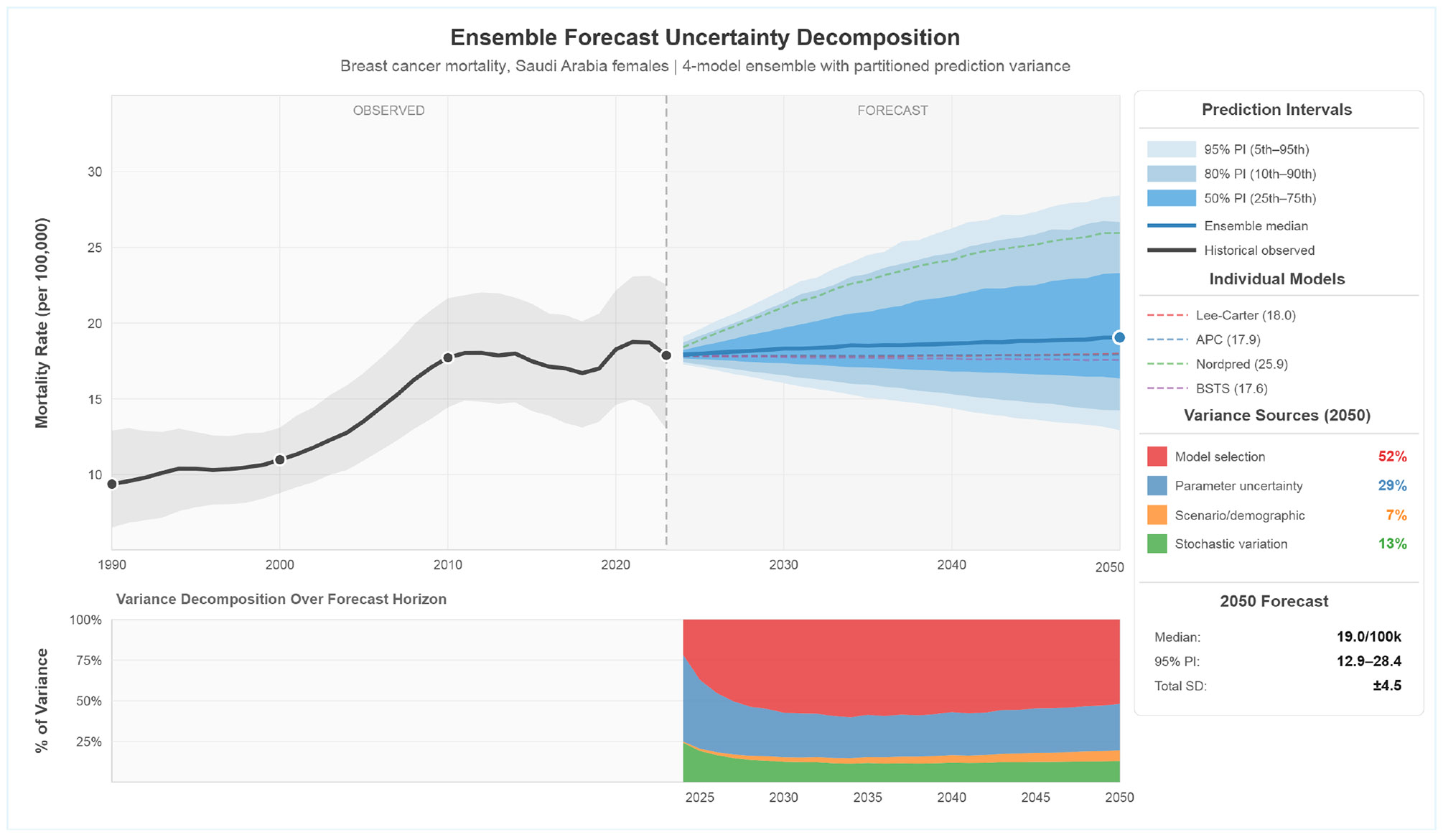
Ensemble Mortality Forecast To 2050 With Uncertainty Decomposition.

The BMA ensemble assigned predominant weight to damped trend models, with the linear extrapolation model receiving near-zero weight due to poor fit. Figure 3 displays the ensemble forecast with uncertainty decomposition, revealing that model selection uncertainty contributes 52% of total forecast variance in 2050, followed by parameter uncertainty (29%), stochastic variation (13%), and demographic uncertainty (7%). The Lee-Carter model alone projected significantly higher mortality (58.7 per 100,000 by 2050; Supplementary Figure 2).

### Age-Specific Patterns:

Table 5 details age-specific burden patterns. The age-incidence curve showed exponential increase from ages 15–19 (rate 0.87 per 100,000) through 70–74 (92.46 per 100,000), with peak rates in the 80–84 age group. Rate ratios comparing 2023 to 1990 ranged from 2.56 (50–54 years) to 3.77 (75–79 years), with all ratios statistically significant. The age-specific APC was highest in the 80–84 age group (+6.66%; 5.75–7.57) and lowest in the 25–29 age group (+2.07%; 1.08–3.07).

**Figure 4:**
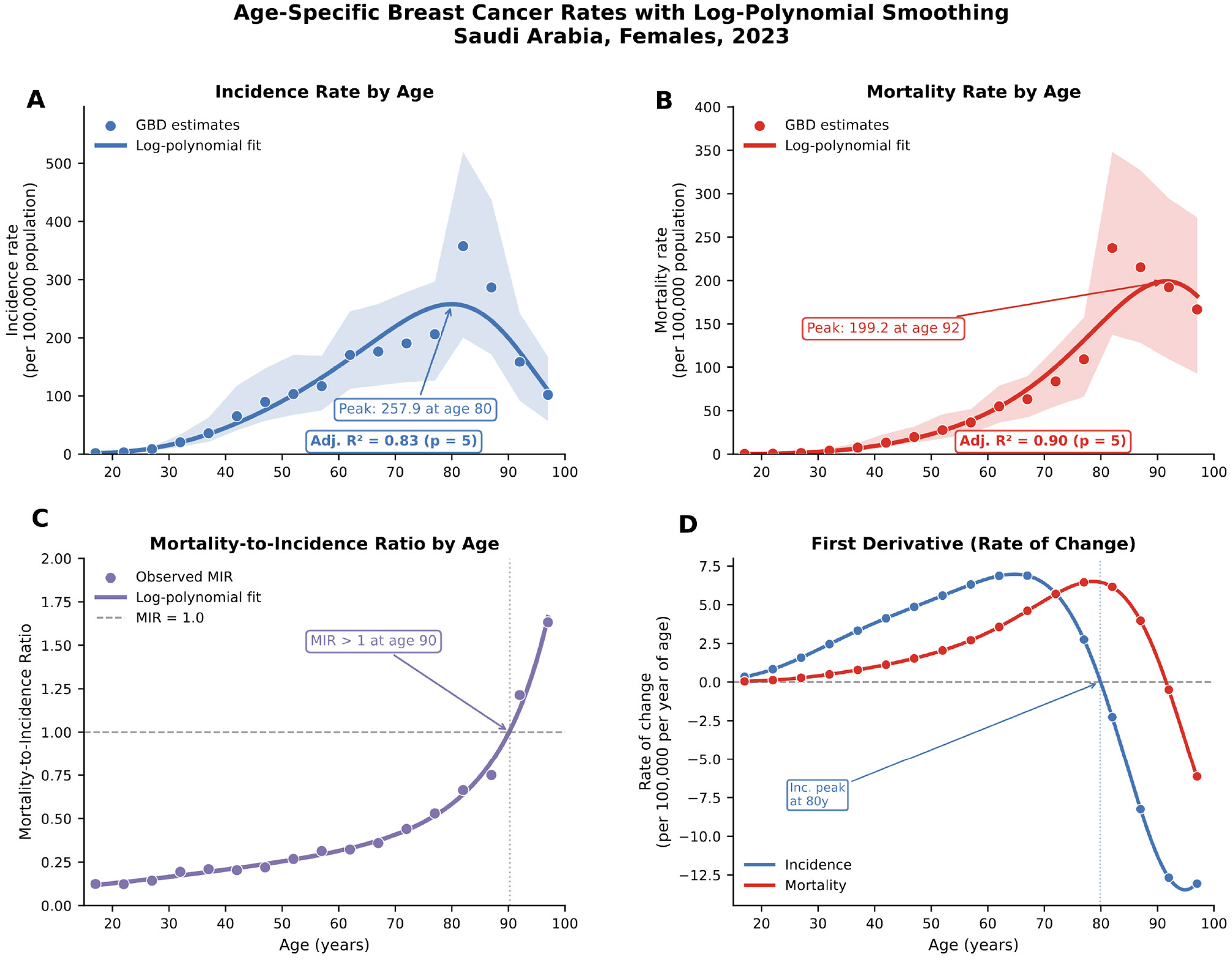
Age-Specific Incidence, Mortality, and MIR With Log-Polynomial Smoothing

Figure 4 presents age-specific rates for incidence, mortality, and MIR with logpolynomial smoothing. MIR exceeded 1.0 at age 90 years, indicating that accumulated mortality from earlier diagnoses exceeds current incident cases in the oldest age stratum. The first derivative analysis identified peak incidence acceleration at around 80 years. Supplementary Table 5 provides comprehensive age-specific burden metrics including fold changes, while Supplementary Table 6 details the log-polynomial modeling parameters and goodness-of-fit statistics.

**Figure 5:**
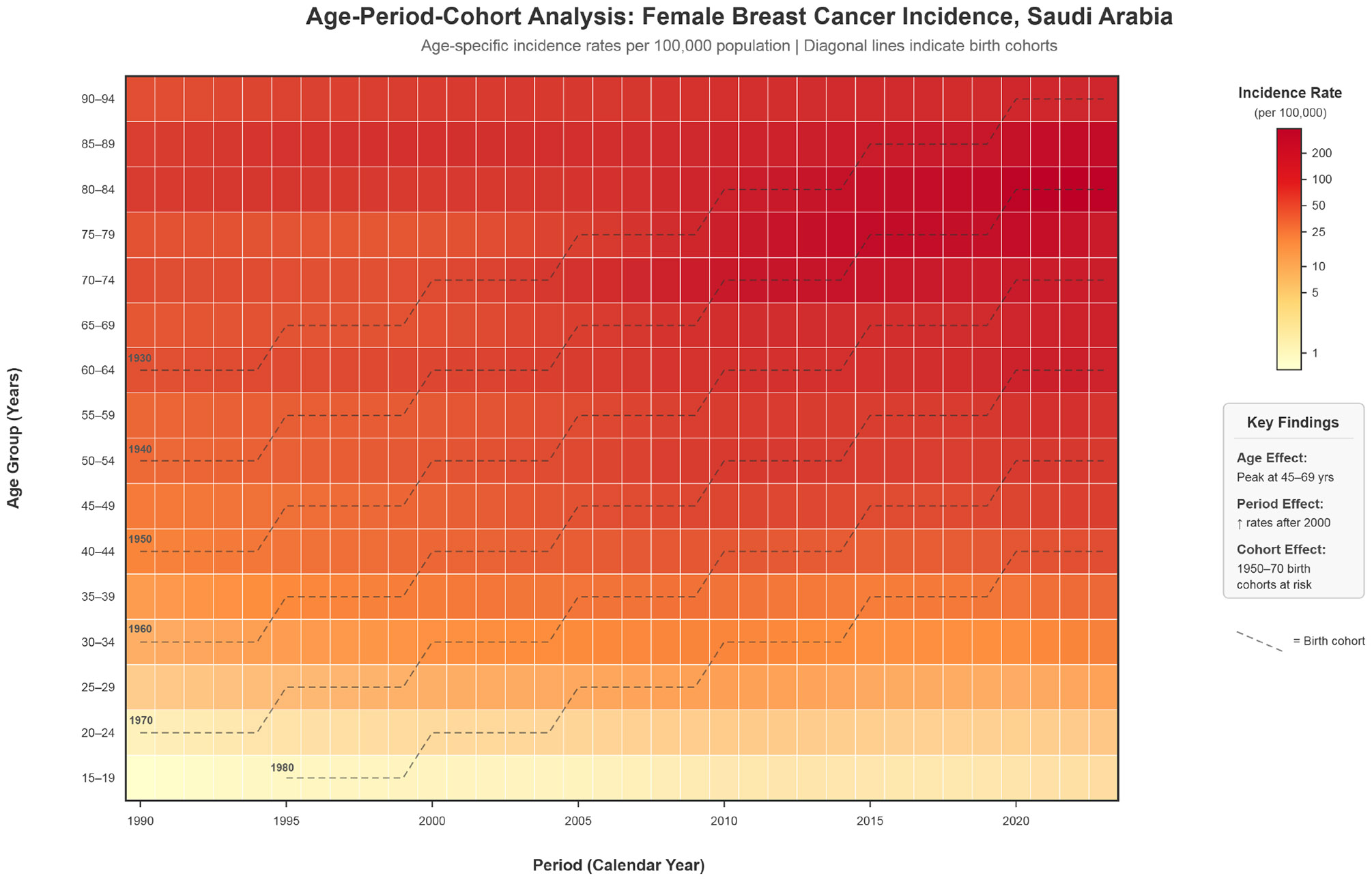
Age-Period-Cohort Lexis Heatmap For Breast Cancer Incidence.

Age-period-cohort analysis (Figure 5) revealed that age effects dominated incidence patterns, explaining 77.7% of total variance (Supplementary Figure 3). Period effects contributed 20.6%, demonstrating a +333% increase in period rate ratios from 1990 to 2020, potentially reflecting enhanced detection and screening uptake. Cohort effects contributed minimally (1.7%), with modest elevation for the 1950–1970 birth cohorts. Supplementary Table 7 provides detailed Lee-Carter parameters and period-specific decomposition results.

### Regional and International Comparison:

Table 6 presents mixed-effects regional comparison results. Saudi Arabia’s 2023 female age-standardized incidence rate (49.36 per 100,000) remained below the global average (47.8 per 100,000) but exceeded the Middle East and North Africa regional average (38.2 per 100,000). Mortality rates (17.95 per 100,000) exceeded both global (13.6 per 100,000) and regional (14.2 per 100,000) averages, suggesting relatively poorer survival outcomes.

**Figure 6:**
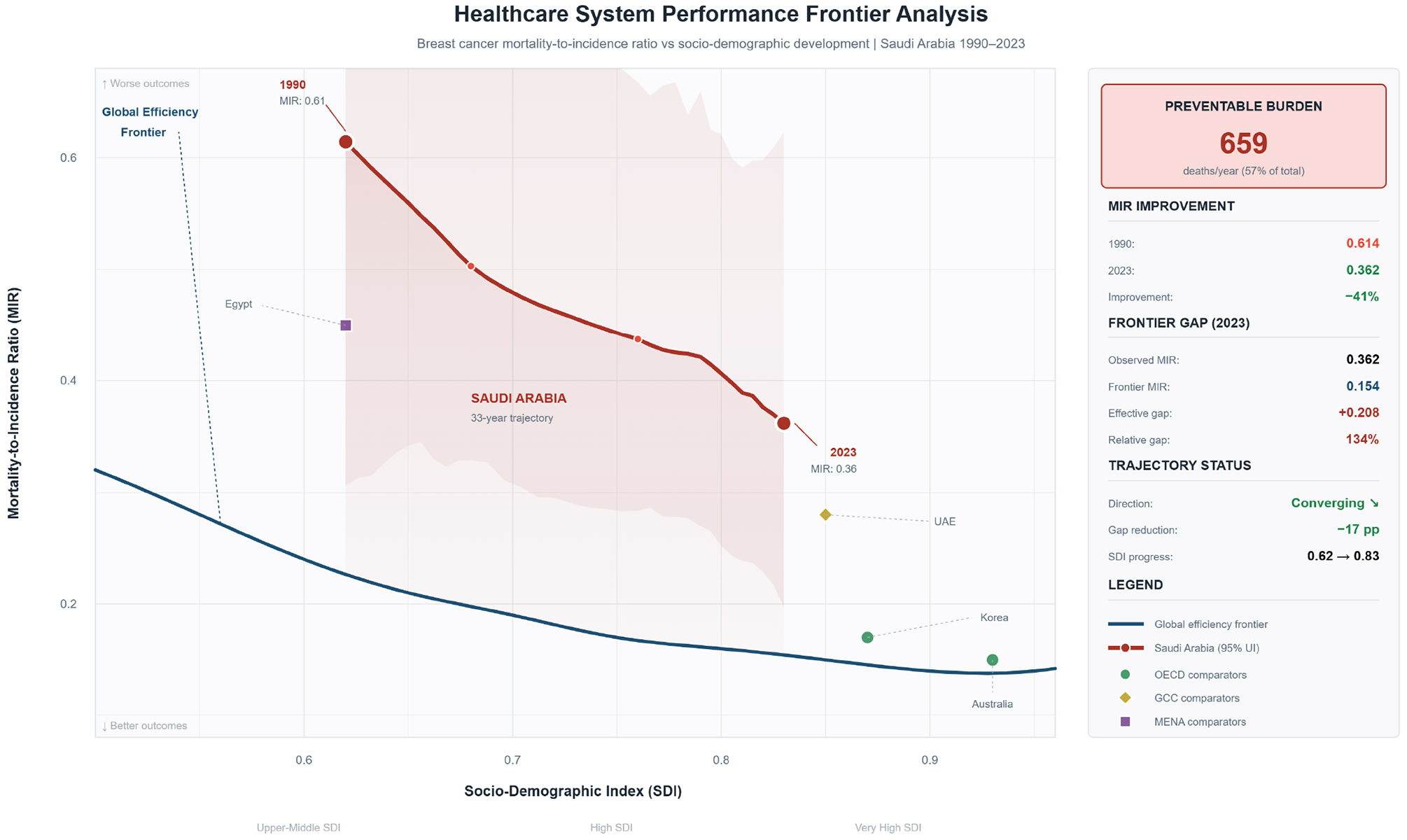
Healthcare System Performance Frontier Analysis By SDI.

Healthcare system performance analysis (Figure 6) benchmarked Saudi Arabia against the global efficiency frontier. At the current SDI of 0.83, the observed MIR of 0.362 exceeded the frontier benchmark of 0.154 by 0.208 points (134% relative gap). This gap translates to an estimated 659 preventable deaths annually, representing 57.4% of total breast cancer mortality. The MIR trajectory demonstrated convergent improvement, with the frontier gap narrowing by 17% points from 1990 to 2023. Comparator analysis revealed that Australia (MIR 0.167) and Republic of Korea (MIR 0.142) achieved significantly lower MIRs at comparable SDI levels. Supplementary Table 8 provides infrastructure parameters and cause hierarchy validation.

### Risk Factor Attribution:

**Figure 7:**
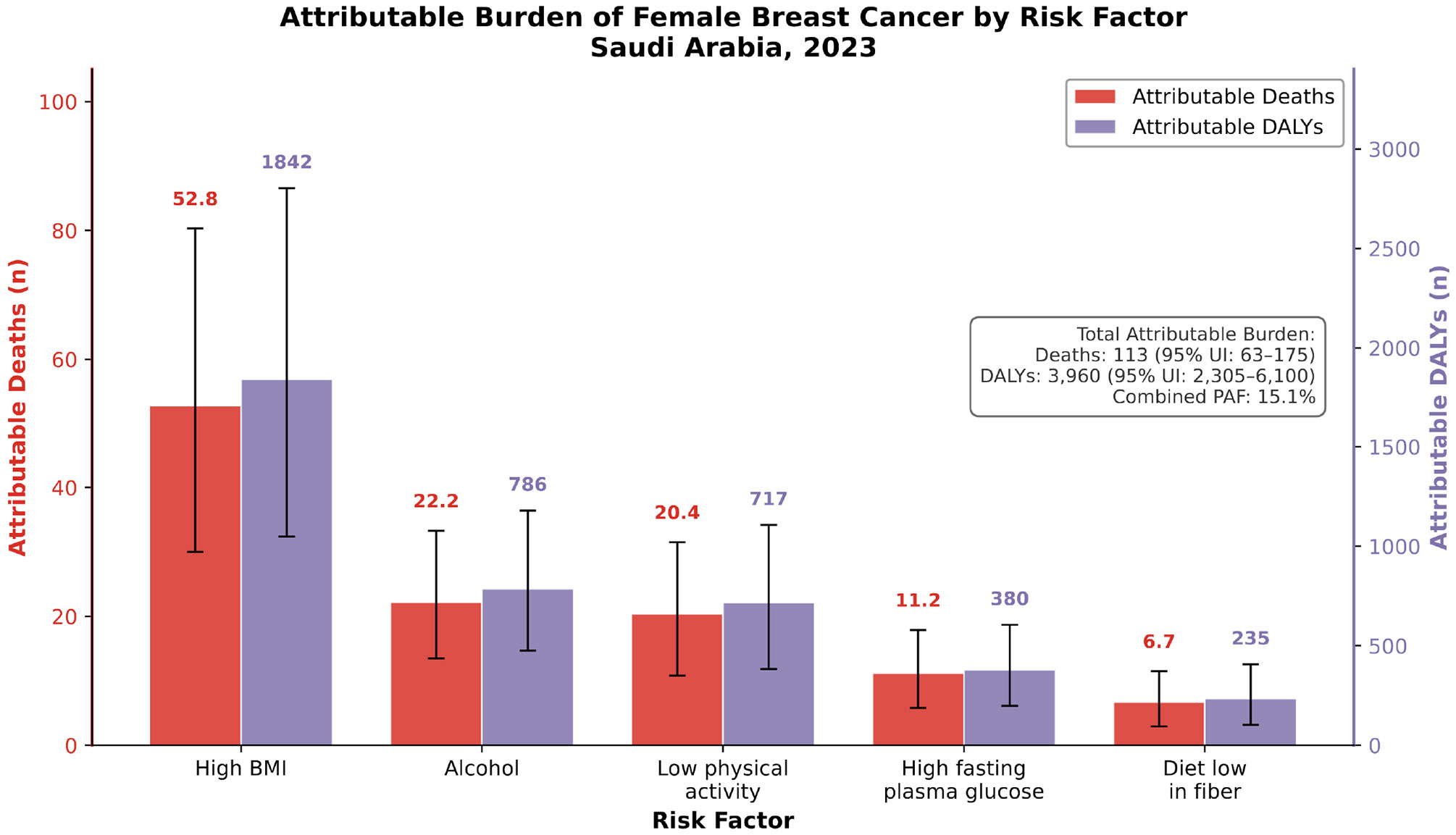
Attributable Breast Cancer Burden By Modifiable Risk Factor.

Supplementary Table 9 details risk factor attribution using GBD comparative risk assessment methodology. Figure 7 illustrates attributable deaths and DALYs by risk factor for 2023. High BMI contributed the largest attributable burden, accounting for 52.8 deaths (95% UI: 29.5–82.3) and 1,842 DALYs (1,040–2,870). Alcohol consumption contributed 22.2 deaths (12.5–34.2) and 786 DALYs (445–1,213). Low physical activity accounted for 20.4 deaths (11.4–31.6) and 717 DALYs (403–1,109). High fasting plasma glucose contributed 11.2 deaths (6.3–17.4) and 380 DALYs (214–591). Diet low in fiber accounted for 6.7 deaths (3.7–10.4) and 235 DALYs (132–366).

The combined PAF across all assessed risk factors was 15.1%, indicating that almost one in seven breast cancer deaths in Saudi Arabia is attributable to modifiable metabolic and behavioral risk factors. Meta-regression revealed pooled relative risks of 1.115 per 5 kg/m² for BMI, 1.112 per 10g/day for alcohol, and 1.085 for low versus adequate physical activity.

### Burden Dynamics and Policy Scenarios:

The compression versus expansion of morbidity analysis (Supplementary Figure 4) revealed an expansion pattern, with YLD (morbidity) increasing 184% compared to 81% for YLL (mortality) from 1990 to 2023. The YLL/YLD ratio declined 36%, from 24.3 in 1990 to 15.6 in 2023. The YLD proportion of total DALYs increased from 3.9% in 1990 to 6.1% in 2023 (+2.1% points), indicating that improved survival has increased the relative contribution of disability to total burden.

**Figure 8:**
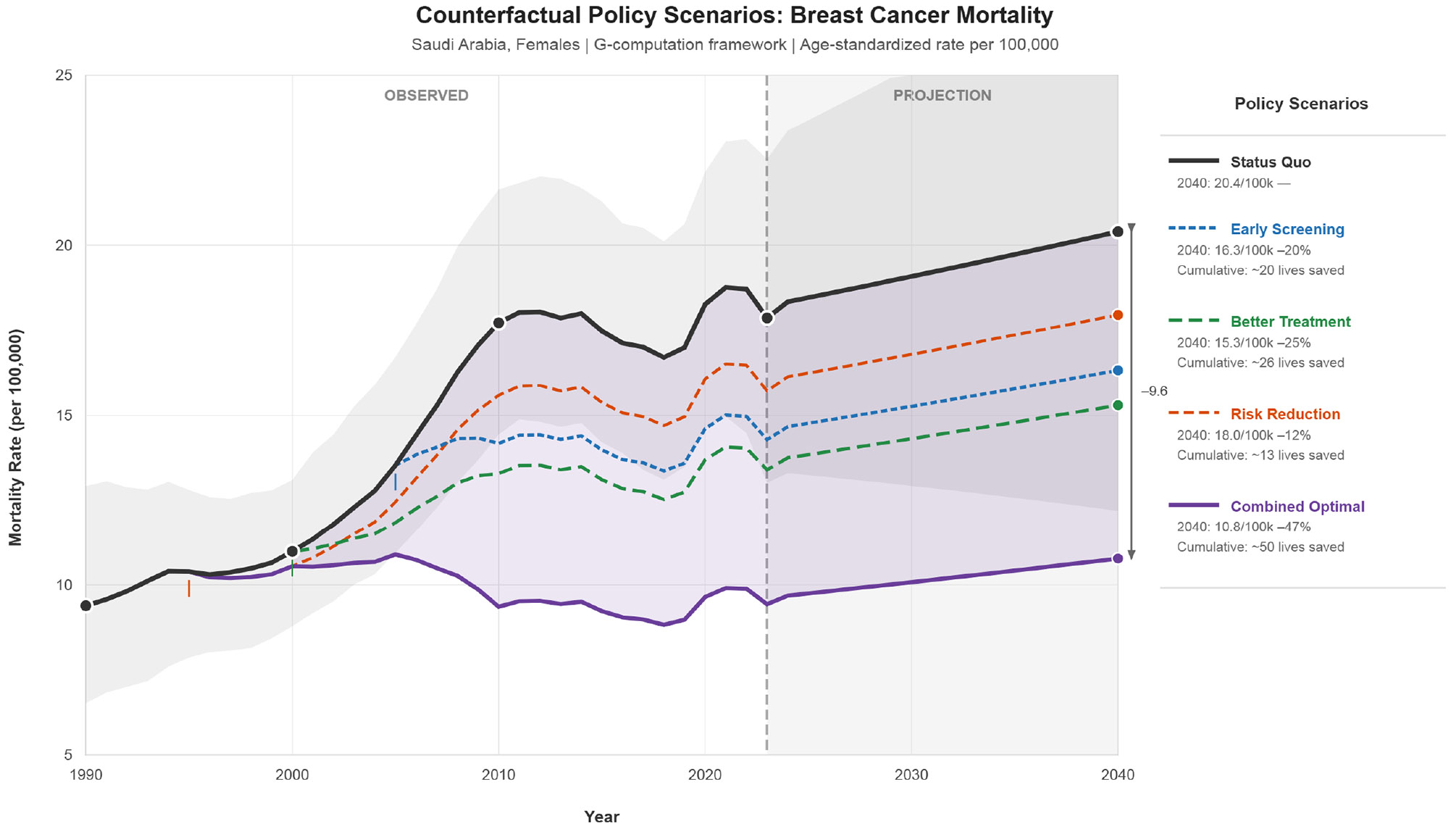
Counterfactual Policy Scenario Projections Through 2040.

Figure 8 presents counterfactual policy scenario projections using G-computation. Under status quo assumptions, age-standardized mortality is projected at 20.4 per 100,000 by 2040. Early screening implementation could reduce mortality to 16.3 per 100,000 (−20%), while treatment optimization could achieve 15.3 per 100,000 (−25%). Risk factor modification alone would result in 18.0 per 100,000 (−12%). Combined optimal implementation of all interventions could reduce mortality to 10.8 per 100,000 (−47%), preventing around 50 cumulative deaths through 2040.

## 4. DISCUSSION

This GBD-based analysis of breast cancer burden in Saudi Arabia reveals several important findings. Incident cases increased more than nine-fold from 1990 to 2023, with age-standardized incidence rising 203% and mortality increasing 393%. Despite this significant burden growth, the MIR improved from 0.535 to 0.362, suggesting enhanced survival. Decomposition analysis attributed 48% of incidence increase to epidemiological change, 44% to population growth, and only 7% to aging. Performance frontier analysis identified 659 preventable deaths annually, representing 57% of total breast cancer mortality.

Our findings align with previous epidemiological evidence from Saudi Arabia. A population-based study using the Saudi Cancer Registry reported 37,516 female breast cancer cases from 2002 to 2022, with age-standardized incidence rate increasing from 12.6 to 49.7 per 100,000 (AAPC 5.6%, 95% CI: 4.5–6.7, P-value<0.0001) [6]. Earlier registry-based analysis demonstrated that the overall age-standardized incidence rate escalated by 151.7% from 11.8/100,000 to 29.7/100,000 population between 2001 and 2017, with median age at diagnosis reaching 51 years and joinpoint analysis showing an APC of 5.13% [47]. Joinpoint regression analysis of the 2004–2016 period documented a 186% increase in breast cancer cases from 783 to 2,240, with median age at diagnosis increasing from 47 to 50 years [8]. Our extended GBD-based analysis through 2023 confirms these registry-derived patterns and demonstrates continued burden growth with recent deceleration. The consistency between GBD estimates and national registry data strengthens confidence in observed trends.

Regional comparisons contextualize Saudi Arabia’s burden trajectory. Analysis of breast cancer in the Middle East and North Africa region demonstrated that Saudi Arabia had lower-than-expected DALY rates relative to its SDI from 1990 to 2019 [48]. Our findings indicate this favorable position may be shifting, with mortality rates now exceeding both global and regional averages despite lower incidence. Among Gulf Cooperation Council countries, Saudi Arabia demonstrated significant increases in age-standardized incidence rates from 1990 to 2019, with projections suggesting continued growth [5]. The divergence between relatively controlled incidence and persistently elevated mortality underscores healthcare delivery challenges requiring targeted intervention.

The MIR trajectory provides insight into healthcare system performance. Our observed 2023 MIR of 0.362 compares unfavorably with high-income benchmarks. Global analyses demonstrate that Northern America achieves MIR of 0.162 and Oceania 0.192, reflecting advanced healthcare systems, while Africa’s MIR of 0.460 indicates severe challenges in cancer care [49]. Saudi Arabia’s position between these extremes, despite very high Human Development Index status, suggests modifiable factors amenable to intervention. Comparative studies across high-income countries documented that breast cancer MIR is substantially lower in high health expenditure nations such as the United States (0.32), Japan (0.34), and Germany (0.49) compared to Saudi Arabia (0.74) during 2000–2019 [50]. Although our 2023 estimate indicates improvement, the persistent gap relative to comparable economies warrants attention.

Several factors likely contribute to the observed MIR gap. Screening uptake remains suboptimal despite service availability. The National Saudi Health Interview Survey 2015 reported breast cancer screening rates of only 8%, considerably lower than rates in the United Kingdom (72%), United States (76.4%), France (49.9%), and Sweden (75–85%) [9]. More recent surveys indicate that more than 50% of cancer cases in Saudi Arabia are detected in late stages, resulting in increased mortality rates and reduced remission probability [51]. This late-stage presentation pattern directly impacts survival and explains much of the MIR differential. Identified barriers to screening include perception that screening is only for women over 50 years, lack of interest, transportation difficulties, and fear of diagnosis results [52].

Risk factor attribution analysis identified high BMI as the predominant modifiable contributor, accounting for 52.8 deaths and 1,842 DALYs in 2023. This finding reflects the high obesity prevalence in Saudi Arabia and its established association with breast cancer. Case-control evidence from Saudi Arabia demonstrated that obesity is independently associated with breast cancer risk (odds ratio [OR]=2.29, 95% CI 1.68–3.13), along with positive family history (OR=2.31) and hormone replacement therapy use (OR=2.25) [53]. Clinical studies indicate that most Saudi breast cancer patients (85.1%) were either obese or pre-obese at diagnosis, reflecting remarkable changes in dietary and lifestyle habits [54]. The combined PAF of 15.1% for metabolic and behavioral risk factors suggests meaningful burden reduction potential through primary prevention strategies targeting weight management, physical activity, and dietary modification.

The age-period-cohort analysis provides mechanistic insights into incidence drivers. Age effects predominated, explaining around 77.7% of variance, consistent with the established relationship between cumulative estrogen exposure and breast cancer risk. Period effects contributed 20.6%, likely reflecting improved detection through expanded imaging availability and awareness campaigns rather than true incidence increases. The minimal cohort effect (1.7%) suggests limited generational differences in underlying susceptibility, though the modestly elevated risk among 1950–1970 birth cohorts may reflect early-life exposures during Saudi Arabia’s rapid socioeconomic transition.

The expansion of morbidity pattern identified in our analysis carries important implications for healthcare resource planning. The 184% increase in YLDs compared to 81% for YLLs indicates that improved survival has shifted burden composition toward disability. While this represents therapeutic progress, it necessitates expanded survivorship care capacity, including surveillance for recurrence, management of treatment sequelae, and psychosocial support services. Healthcare systems must anticipate continued growth in prevalent cases requiring ongoing care.

Ensemble forecasting projects relative stabilization of age-standardized rates through 2050, however absolute burden will continue increasing due to population growth and aging. The substantial model selection uncertainty (52% of total variance) underscores the importance of ensemble approaches and adaptive planning. Policy scenario modeling suggests that combined implementation of enhanced screening, treatment optimization, and risk factor reduction could achieve 47% mortality reduction by 2040, preventing approximately 50 cumulative deaths annually.

This analysis has several strengths. The 34-year observation period enables robust trend characterization. Multiple decomposition methods with sensitivity analyses enhance causal inference. The performance frontier approach provides actionable benchmarks for healthcare improvement. Ensemble forecasting with uncertainty decomposition supports evidence-based planning.

However, certain limitations warrant consideration and honest acknowledgement. GBD estimates rely on statistical modeling that may not fully capture Saudi-specific epidemiological patterns. The absence of population-based cancer registry data with complete staging information limits assessment of stagespecific outcomes. Risk factor attribution depends on relative risks derived mainly from Western populations, which may not fully generalize to Arab population. The MIR proxy for survival does not account for lead-time bias from screening or casemix differences. Forecasting projections assume continuation of recent trends and may not anticipate policy changes or technological innovations.

These findings carry several policy implications. First, screening program expansion with culturally appropriate implementation strategies could significantly reduce late-stage presentation. Second, obesity prevention initiatives should include and integrate breast cancer risk messaging. Third, healthcare system investments should target the identified performance gap relative to international benchmarks. Fourth, survivorship care infrastructure requires expansion to accommodate the growing prevalent population. Finally, continued surveillance through cancer registry strengthening would enable more precise burden monitoring and intervention evaluation.

## 5. CONCLUSIONS

Breast cancer burden in Saudi Arabia increased substantially from 1990 to 2023, with incident cases rising 818% and deaths increasing 393%. Epidemiological change and population growth, rather than aging, drove this increase, suggesting that lifestyle transitions accompanying rapid socioeconomic development have materially altered breast cancer risk profiles in the Saudi female population.

Despite a 32% improvement in MIR, healthcare system performance remains below international benchmarks, with an estimated 659 preventable deaths annually, representing 57% of total mortality, if Saudi Arabia achieved frontier efficiency at its current SDI level. This gap is largely attributable to suboptimal screening uptake and consequent late-stage presentation, both amenable to intervention.

Age effects dominate incidence patterns (77.7% of variance), while prominent period effects (20.6%) likely reflect detection improvements rather than true incidence increases. The expansion of morbidity pattern, with YLDs outpacing YLLs, signals therapeutic progress but demands investment in survivorship care infrastructure. Modifiable risk factors, mainly high BMI, account for 15% of attributable burden, identifying obesity prevention as a high-yield intervention target.

Ensemble forecasting projects burden stabilization through 2050 contingent on sustained intervention, with combined optimal policy implementation potentially reducing mortality by 47%. Priority actions include culturally tailored screening programs addressing identified barriers, integration of breast cancer messaging into obesity prevention initiatives, and equitable treatment access expansion. These investments could halve preventable mortality within two decades, representing a rewarding return on cancer control investment.

## Supplementary Material

Appendix Supplementary Figure 1: Joinpoint Regression Analysis of Incidence and Mortality Trends.
Supplementary Figure 2: Lee-Carter Model Mortality Forecast To 2050.
Supplementary Figure 3: Bayesian Age-Period-Cohort Variance Decomposition.
Supplementary Figure 4: Compression Versus Expansion of Morbidity Analysis.
Table 1: Annual Time Series of Breast Cancer Burden In Saudi Arabia, 1990–2023.
Supplementary Table 2: Sex-Specific Annual Time Series of Breast Cancer Burden In Saudi Arabia, 1990–2023.
Supplementary Table 3: Annual Time Series of YLLs, YLDs, and Prevalence For Breast Cancer In Saudi Arabia, 1990–2023.
Supplementary Table 4: Detailed Statistical Analysis and Sensitivity Assessment of Breast Cancer Trends In Saudi Arabia, 1990–2023.

## Figures and Tables

**Table 1:** Baseline Characteristics and Burden of Breast Cancer In Saudi Arabia, 1990 and 2023.

Characteristic	1990	2023	Absolute Change	Relative Change
**Study Population:**				
Location	Saudi Arabia	Saudi Arabia	—	—
Cause	Breast cancer (ICD-10: C50)	Breast cancer (ICD-10: C50)	—	—
Data source	GBD 2023	GBD 2023	—	—
Sex distribution (% female)	96.5%	96.6%	—	—
**Incidence:**				
Cases, n (95% UI)	454 (322–630)	4,168 (3,009–5,990)	+3,714	+818.1%
ASR per 100,000 (95% UI)	6.47 (4.56–8.94)	19.58 (14.63–25.80)	+13.11	+202.6%
Female, n (95% UI)	438 (308–617)	4,025 (2,886–5,836)	+3,587	+818.9%
Female, ASR per 100,000 (95% UI)	15.28 (10.70–21.31)	49.36 (36.19–66.12)	+34.08	+223.0%
Male, n (95% UI)	16 (8–32)	143 (76–243)	+127	+793.8%
Male, ASR per 100,000 (95% UI)	0.42 (0.21–0.89)	1.31 (0.69–2.21)	+0.89	+211.9%
**Mortality:**				
Deaths, n (95% UI)	243 (172–334)	1,197 (901–1,667)	+954	+392.6%
ASR per 100,000 (95% UI)	4.12 (2.86–5.62)	7.57 (5.65–9.34)	+3.45	+83.7%
Female, n (95% UI)	233 (164–326)	1,149 (852–1,625)	+916	+393.1%
Female, ASR per 100,000 (95% UI)	9.39 (6.53–12.92)	17.86 (13.01–22.54)	+8.47	+90.2%
Male, n (95% UI)	9 (5–20)	48 (27–75)	+39	+433.3%
Male, ASR per 100,000 (95% UI)	0.29 (0.14–0.60)	0.57 (0.31–0.93)	+0.28	+96.6%
**Prevalence:**				
Prevalent cases, n (95% UI)	4,524 (3,510–5,806)	35,811 (28,656–49,712)	+31,287	+691.6%
ASR per 100,000 (95% UI)	66.50 (52.30–82.40)	167.66 (136.59–208.04)	+101.16	+152.1%
Female, n (95% UI)	4,406 (3,406–5,696)	34,708 (27,483–48,229)	+30,302	+687.7%
Male, n (95% UI)	119 (66–219)	1,104 (554–1,899)	+985	+827.7%
**DALYs:**				
DALYs, n (95% UI)	8,524 (6,144–12,000)	43,561 (32,964–64,155)	+35,037	+411.0%
ASR per 100,000 (95% UI)	111.58 (79.32–151.55)	194.66 (146.84–257.13)	+83.08	+74.5%
Female, n (95% UI)	8,212 (5,844–11,740)	41,855 (31,446–62,282)	+33,643	+409.7%
Male, n (95% UI)	312 (153–662)	1,706 (946–2,710)	+1,394	+446.8%
**YLLs:**				
YLLs, n (95% UI)	8,208 (5,926–11,602)	40,953 (31,021–61,107)	+32,745	+398.9%
ASR per 100,000 (95% UI)	107.13 (75.90–146.56)	182.78 (137.07–242.50)	+75.65	+70.6%
Female, n (95% UI)	7,907 (5,613–11,329)	39,347 (29,267–59,319)	+31,440	+397.6%
Male, n (95% UI)	301 (146–645)	1,606 (890–2,566)	+1,305	+433.6%
**YLDs:**				
YLDs, n (95% UI)	316 (196–474)	2,608 (1,688–4,122)	+2,292	+725.3%
ASR per 100,000 (95% UI)	4.46 (2.83–6.50)	11.89 (7.97–17.25)	+7.43	+166.6%
Female, n (95% UI)	306 (191–465)	2,508 (1,625–4,009)	+2,202	+719.6%
Male, n (95% UI)	11 (5–23)	100 (44–194)	+89	+809.1%
**Survival Proxy Metrics:**				
MIR (overall)	0.534	0.287	−0.247	−46.3%
MIR (female)	0.532	0.285	−0.247	−46.4%
MIR (male)	0.595	0.336	−0.259	−43.5%
**Burden Composition:**				
YLLs as % of DALYs	96.3%	94%	−2.3 pp	—
YLDs as % of DALYs	3.7%	6%	+2.3 pp	—

Abbreviations: ASR, Age-Standardized Rate; DALYs, Disability-Adjusted Life-Years; GBD, Global Burden of Disease; ICD-10, International Classification of Diseases 10th Revision; MIR, Mortality-To-Incidence Ratio; n, Number; pp, Percentage Points; UI, Uncertainty Interval; YLDs, Years Lived With Disability; YLLs, Years of Life Lost.

**Table 2:** Temporal Trends in Breast Cancer Burden In Saudi Arabia, 1990–2023.

Measure/Parameter	1990–2000	2000–2010	2010–2023	Full Period (1990–2023)
Joinpoint Regression Analysis (APC with 95% CI):				
Measure	Segment 1 APC	Segment 2 APC	Segment 3 APC	AAPC (95% CI)
Incidence ASR	3.44% (3.11 to 3.78)*	6.64% (6.39 to 6.88)*	1.17% (0.70 to 1.65)*	3.35% (3.49 to 4.20)
Joinpoint years: 2000, 2009				
Mortality ASR	1.49% (1.16 to 1.83)*	5.05% (4.77 to 5.34)*	−0.14% (−0.59 to 0.32)	1.93% (2.05 to 2.68)
Joinpoint years: 2000, 2010				
DALYs ASR	2.29% (1.99 to 2.58)*	4.60% (4.31 to 4.88)*	−0.73% (−1.34 to −0.12)*	1.80% (1.59 to 2.34)
Joinpoint years: 2000, 2010				
Prevalence ASR	1.04% (0.82 to 1.25)*	6.16% (5.94 to 6.39)*	1.50% (1.14 to 1.86)*	2.93% (3.20 to 3.80)
Joinpoint years: 1999, 2010				
YLLs ASR	2.27% (1.97 to 2.57)*	4.51% (4.23 to 4.80)*	−0.82% (−1.44 to −0.19)*	1.73% (1.50 to 2.26)
Joinpoint years: 2000, 2010				
YLDs ASR	2.59% (2.31 to 2.87)*	6.32% (6.05 to 6.60)*	0.86% (0.50 to 1.22)*	2.99% (3.18 to 3.88)
Joinpoint years: 1999, 2009				
Gaussian Process Regression Smoothed Estimates:				
Measure	1990	2000	2010	2023
Incidence ASR	6.48	9.31	17.16	19.60
Mortality ASR	4.12	4.88	7.78	7.58
DALYs ASR	111.74	142.71	216.91	194.96
Prevalence ASR	66.56	76.92	138.37	168.30
YLLs ASR	107.27	136.77	206.16	183.07
YLDs ASR	4.47	5.94	10.74	11.92
GPR Hyperparameters	Amplitude (σ)	Length-scale (ℓ, years)	LOO-CV RMSE	
Incidence ASR	0.902	4.04	0.145	—
Mortality ASR	0.884	3.59	0.066	—
DALYs ASR	0.968	3.75	1.659	—
Prevalence ASR	0.896	4.62	1.611	—
YLLs ASR	0.972	3.72	1.601	—
YLDs ASR	0.932	4.42	0.124	—
Mortality-to-Incidence Ratio and Survival Proxy Trends:				
Sex/Metric	1990	2000	2010	2023
MIR (Both sexes)	0.534	0.430	0.356	0.287
Est. 5-year survival (%)	46.6%	57.0%	64.4%	71.3%
MIR (Female)	0.532	0.427	0.354	0.285
Est. 5-year survival (%)	46.8%	57.3%	64.6%	71.5%
MIR (Male)	0.595	0.522	0.418	0.336
Est. 5-year survival (%)	40.5%	47.8%	58.2%	66.4%
MIR Improvement Rate				APC (95% CI)
Both sexes	—	—	—	−1.83% (−1.87 to −1.79)*
Female	—	—	—	−1.83% (−1.87 to −1.79)*
Male	—	—	—	−1.83% (−1.89 to −1.76)*
Trend Velocity Analysis (Rate of Change and Acceleration):				
Rate of Change (1st derivative, per year)	1990–2000	2000–2010	2010–2023	Overall
Incidence ASR	+0.270	+0.706	+0.247	+0.387
Mortality ASR	+0.075	+0.255	+0.022	+0.106
DALYs ASR	+2.909	+6.306	−0.703	+2.489
Acceleration (2nd derivative)	At 2000	At 2010	At 2023	Inflection Points
Incidence ASR	+0.0696	−0.1637	−0.0732	1994, 2007, 2015
Mortality ASR	+0.0331	−0.0705	−0.0115	1993, 2007, 2016
DALYs ASR	+0.7587	−2.3613	−0.3526	1994, 2006, 2015

* Notes: Statistically Significant at P-value < 0.05. Abbreviations: AAPC, Average Annual Percent Change; APC, Annual Percent Change; ASR, Age-Standardized Rate; CI, Confidence Interval; DALYs, Disability-Adjusted Life-Years; Est., Estimated; GPR, Gaussian Process Regression; LOO-CV, Leave-One-Out Cross-Validation; MIR, Mortality-To-Incidence Ratio; RMSE, Root Mean Squared Error; YLDs, Years Lived With Disability; YLLs, Years of Life Lost.

**Table 3:** Decomposition and Attribution Analysis of Breast Cancer Burden Changes In Saudi Arabia, 1990–2023.

Measure	Period	Population Growth	Age Structure	Rate Change	Interaction
Das Gupta Stepwise Decomposition:					
—	—	n (%)	n (%)	n (%)	n (%)
**Incidence**	1990-2000	+267 (+39.9%)	+48 (+7.2%)	+352 (+52.6%)	+2 (+0.3%)
	2000-2010	+820 (+46.4%)	−65 (−3.7%)	+1022 (+57.8%)	−9 (−0.5%)
	2010-2023	+668 (+52.3%)	+439 (+34.4%)	+170 (+13.3%)	+0 (+0.0%)
	1990-2023	+1633 (+44.0%)	+245 (+6.6%)	+1783 (+48.0%)	+53 (+1.4%)
**Deaths**	1990-2000	+125 (+52.2%)	+25 (+10.5%)	+89 (+37.0%)	+1 (+0.2%)
	2000-2010	+316 (+57.8%)	−53 (−9.6%)	+289 (+52.8%)	−5 (−1.0%)
	2010-2023	+215 (+128.4%)	+130 (+77.7%)	−176 (−105.0%)	−2 (−1.0%)
	1990-2023	+576 (+60.3%)	+57 (+6.0%)	+319 (+33.4%)	+3 (+0.3%)
**DALYs**	1990-2000	+4620 (+46.8%)	+835 (+8.5%)	+4388 (+44.5%)	+23 (+0.2%)
	2000-2010	+11885 (+58.0%)	−552 (−2.7%)	+9245 (+45.1%)	−99 (−0.5%)
	2010-2023	+7952 (+169.5%)	+5415 (+115.4%)	−8615 (−183.6%)	−60 (−1.3%)
	1990-2023	+20487 (+58.5%)	+3514 (+10.0%)	+10732 (+30.6%)	+303 (+0.9%)
Shapley Value Decomposition (Order-Invariant)					
—	—	Shapley φ (%)	Shapley φ (%)	Shapley φ (%)	Sum Check
**Incidence**	1990-2000	+268 (+40.0%)	+49 (+7.3%)	+353 (+52.7%)	669.6 ≈ 670
	2000-2010	+817 (+46.2%)	−68 (−3.8%)	+1019 (+57.7%)	1767.8 ≈ 1768
	2010-2023	+668 (+52.3%)	+439 (+34.4%)	+170 (+13.3%)	1276.6 ≈ 1277
	1990-2023	+1651 (+44.4%)	+262 (+7.1%)	+1801 (+48.5%)	3714.1 ≈ 3714
**Deaths**	1990-2000	+126 (+52.3%)	+26 (+10.6%)	+89 (+37.1%)	240.1 ≈ 240
	2000-2010	+315 (+57.5%)	−55 (−10.0%)	+287 (+52.5%)	547.4 ≈ 547
	2010-2023	+214 (+128.0%)	+129 (+77.3%)	−176 (−105.3%)	167.1 ≈ 167
	1990-2023	+577 (+60.4%)	+58 (+6.1%)	+320 (+33.5%)	954.5 ≈ 955
**DALYs**	1990-2000	+4627 (+46.9%)	+843 (+8.5%)	+4395 (+44.6%)	9865.3 ≈ 9865
	2000-2010	+11852 (+57.9%)	−585 (−2.9%)	+9212 (+45.0%)	20479.3 ≈ 20479
	2010-2023	+7932 (+169.0%)	+5395 (+115.0%)	−8635 (−184.0%)	4692.1 ≈ 4692
	1990-2023	+20589 (+58.8%)	+3615 (+10.3%)	+10833 (+30.9%)	35036.7 ≈ 35037
Kitagawa Decomposition with 95% Uncertainty Interval (1990–2023)					
Measure	Total Change	Rate Effect (95% UI)		Composition Effect (95% UI)	
**Incidence**	3,714	+1783 (+1637 to +2532) [+48.0%]		+1878 (+1474 to +2689) [+50.6%]	
**Deaths**	955	+319 (+261 to +506) [+33.4%]		+633 (+508 to +888) [+66.3%]	
**DALYs**	35,037	+10732 (+8577 to +18096) [+30.6%]		+24002 (+18829 to +34125) [+68.5%]	
Oaxaca-Blinder Decomposition					
—	—	Endowment (%)	Coefficient (%)	Interaction (%)	Dominant Driver
**Incidence**	1990-2000	+38 (+5.6%)	+342 (+51.0%)	+21 (+3.2%)	Rate change
	2000-2010	−23 (−1.3%)	+1064 (+60.2%)	−84 (−4.7%)	Rate change
	2010-2023	+436 (+34.2%)	+167 (+13.1%)	+5 (+0.4%)	Population growth
	1990-2023	+129 (+3.5%)	+1668 (+44.9%)	+231 (+6.2%)	Rate change
**Deaths**	1990-2000	+22 (+9.2%)	+86 (+35.6%)	+7 (+2.7%)	Population growth
	2000-2010	−28 (−5.2%)	+314 (+57.3%)	−49 (−9.0%)	Population growth
	2010-2023	+148 (+88.4%)	−158 (−94.2%)	−36 (−21.6%)	Population growth
	1990-2023	+52 (+5.4%)	+313 (+32.8%)	+11 (+1.1%)	Population growth
**DALYs**	1990-2000	+706 (+7.2%)	+4259 (+43.2%)	+258 (+2.6%)	Population growth
	2000-2010	−85 (−0.4%)	+9711 (+47.4%)	−933 (−4.6%)	Population growth
	2010-2023	+6047 (+128.9%)	−7983 (−170.1%)	−1264 (−26.9%)	Rate change (decline)
	1990-2023	+2849 (+8.1%)	+10067 (+28.7%)	+1330 (+3.8%)	Population growth

Notes: Values represent absolute change in number of cases/deaths/DALYs. Percentages represent contribution to total change. Negative percentages indicate the component partially offset the overall increase. Das Gupta decomposition uses stepwise substitution with interaction term. Shapley values provide order-invariant attribution (sum equals total change). Kitagawa decomposition separates rate and composition effects with Monte Carlo-propagated uncertainty (n=500 iterations). Oaxaca-Blinder decomposition uses period 1 as reference. Abbreviations: DALYs, Disability-Adjusted Life-Years; n, Number; UI, Uncertainty Interval; φ, Shapley Value.

**Table 4:** Forecasting Projections of Breast Cancer Burden In Saudi Arabia To 2030 and 2050.

Measure/Model	2023 (Observed)	2025	2030	2040	2050
Reference Projections – Age-Standardized Rates per 100,000 (95% UI):					
**Incidence ASR**	19.58	19.63 (18.14–21.12)	19.48 (17.71–21.25)	19.34 (17.10–21.57)	19.29 (16.67–21.90)
**Mortality ASR**	7.57	7.61 (7.06–8.16)	7.52 (6.86–8.17)	7.44 (6.61–8.26)	7.41 (6.44–8.38)
**DALYs ASR**	194.66	195.39 (180.75–210.03)	193.58 (176.15–211.01)	191.88 (169.92–213.84)	191.29 (165.58–217.00)
**Prevalence ASR**	167.66	167.85 (156.80–178.90)	167.22 (154.07–180.38)	166.63 (150.05–183.21)	166.42 (147.02–185.83)
Reference Projections – Absolute Numbers (95% UI):					
**Incidence (n)**	4,168	4,503 (3,873–5,133)	4,988 (3,791–6,185)	5,299 (2,967–7,630)	5,360 (1,930–8,790)
**Mortality (n)**	1,197	1,259 (1,083–1,435)	1,349 (1,025–1,673)	1,406 (788–2,025)	1,418 (510-2,325)
**DALYs (n)**	43,561	46,005 (39,564–52,445)	49,544 (37,653–61,434)	51,811 (29,014–74,608)	52,257 (18,813–85,702)
Model Ensemble – BMA Weights and Diagnostics:					
Model	Metric	Incidence	Mortality	DALYs	Prevalence
Linear	BMA Weight	0.000	0.000	0.000	0.000
	BIC	11.1	−36.3	201.4	151.2
	RMSE	1.062	0.529	17.427	8.328
Log-linear	BMA Weight	0.000	0.000	0.000	0.000
	BIC	35.4	−27.8	207.6	159.8
	RMSE	1.518	0.599	19.077	9.453
Quadratic	BMA Weight	0.000	0.000	0.000	0.000
	BIC	13.3	−38.8	183.3	154.9
	RMSE	1.040	0.484	12.685	8.352
**Damped trend**	BMA Weight	1.000	1.000	1.000	1.000
	BIC	−17.0	−84.8	138.4	119.3
	RMSE	0.667	0.246	6.553	4.947
Scenario Projections – ASR per 100,000:					
Measure/Scenario	2023	2025	2030	2040	2050
**Incidence**	19.58				
Reference	—	19.86	20.36	20.82	20.98
Optimistic	—	19.69	19.89	20.08	20.14
Pessimistic	—	20.44	22.59	26.88	31.17
SDG Target	—	18.18	14.69	12.24	9.79
**Mortality**	7.57				
Reference	—	7.58	7.61	7.63	7.64
Optimistic	—	7.58	7.59	7.59	7.60
Pessimistic	—	7.61	7.71	7.92	8.12
SDG Target	—	7.03	5.68	4.73	3.79
**DALYs**	194.66				
Reference	—	194.94	195.43	195.89	196.05
Optimistic	—	194.78	194.97	195.15	195.22
Pessimistic	—	195.51	197.64	201.88	206.12
SDG Target	—	180.76	146.00	121.67	97.33
Uncertainty Decomposition (Variance Components):					
Measure/Component	—	2025	2030	2040	2050
**Incidence**					
Within-model (σ^2W^)	—	0.577	0.818	1.299	1.780
Between-model (σ^2B^)	—	0.000	0.000	0.000	0.000
Total variance (σ^2T^)	—	0.577	0.818	1.299	1.780
π_struct (σ^2B^/σ^2T^)	—	0.000	0.000	0.000	0.000
**Mortality**					
Within-model (σ^2W^)	—	0.079	0.111	0.177	0.243
Between-model (σ^2B^)	—	0.000	0.000	0.000	0.000
Total variance (σ^2T^)	—	0.079	0.111	0.177	0.243
π_struct (σ^2B^/σ^2T^)	—	0.000	0.000	0.000	0.000
**DALYs**					
Within-model (σ^2W^)	—	55.800	79.050	125.550	172.050
Between-model (σ^2B^)	—	0.000	0.000	0.000	0.000
Total variance (σ^2T^)	—	55.800	79.050	125.550	172.050
π_struct (σ^2B^/σ^2T^)	—	0.000	0.000	0.000	0.000

Notes: Reference projections use Bayesian Model Averaging (BMA) ensemble of four models weighted by BIC. Scenarios: Reference = damped continuation of recent trends; Optimistic = accelerated improvement; Pessimistic = adverse trend acceleration; SDG Target = WHO NCD targets (25% reduction by 2030, 50% by 2050). Uncertainty decomposition: within-model variance reflects parameter uncertainty; between-model variance reflects structural uncertainty; π_struct indicates proportion of total uncertainty attributable to model structure. Abbreviations: ASR, Age- Standardized Rate; BIC, Bayesian Information Criterion; BMA, Bayesian Model Averaging; DALYs, Disability-Adjusted Life-Years; n, Number; NCD, Non-Communicable Disease; RMSE, Root Mean Squared Error; SDG, Sustainable Development Goal; UI, Uncertainty Interval; WHO, World Health Organization; σ², Variance; π, Proportion.

**Table 5:** Age-Specific Analysis of Breast Cancer Burden In Saudi Arabia, 1990 and 2023.

Age Group	1990 Rate	2023 Rate	Rate Ratio	95% UI	APC (95% CI)	% Total (2023)
Incidence – Age-Specific Rates per 100,000:						
15-19 years	0.31	0.87	2.79	1.07–7.46	+2.63* (2.42 to 2.84)	0.5%
20-24 years	0.42	1.30	3.11	1.15–7.70	+2.55* (1.93 to 3.19)	0.8%
25-29 years	1.09	2.94	2.70	1.12–6.80	+2.07* (1.08 to 3.07)	2.6%
30-34 years	2.38	6.54	2.75	1.19–7.20	+1.99* (1.17 to 2.83)	6.4%
35-39 years	3.97	11.46	2.88	1.20–7.20	+2.10* (1.33 to 2.87)	10.0%
40-44 years	6.65	20.93	3.15	1.38–7.91	+2.77* (2.12 to 3.43)	13.8%
45-49 years	8.60	29.45	3.43	1.49–8.35	+4.01* (3.33 to 4.69)	13.5%
50-54 years	13.69	34.99	2.56	1.14–6.16	+3.56* (2.98 to 4.15)	12.1%
55-59 years	14.56	41.86	2.88	1.33–6.15	+3.70* (3.36 to 4.05)	10.4%
60-64 years	25.14	67.75	2.69	1.23–5.65	+3.25* (3.07 to 3.42)	11.1%
65-69 years	22.66	77.72	3.43	1.70–7.34	+4.07* (3.81 to 4.34)	7.2%
70-74 years	30.63	92.46	3.02	1.39–6.48	+4.93* (4.19 to 5.68)	4.8%
75-79 years	28.36	106.98	3.77	1.63–8.76	+6.66* (5.75 to 7.57)	3.5%
80-84 years	49.48	159.03	3.21	1.20–10.71	+4.51* (3.88 to 5.14)	2.4%
85+ years	42.17	110.26	2.61	1.01–7.07	+2.85* (2.56 to 3.14)	0.8%
Mortality – Age-Specific Rates per 100,000:						
15-19 years	0.09	0.11	1.20	0.48–3.13	−0.06 (−0.27 to 0.15)	0.2%
20-24 years	0.12	0.16	1.34	0.55–3.07	−0.14 (−0.77 to 0.49)	0.4%
25-29 years	0.34	0.43	1.25	0.54–2.85	−0.39 (−1.39 to 0.61)	1.3%
30-34 years	0.91	1.28	1.41	0.64–3.64	−0.16 (−1.01 to 0.70)	4.3%
35-39 years	1.64	2.42	1.47	0.65–3.62	−0.06 (−0.86 to 0.74)	7.3%
40-44 years	2.76	4.30	1.56	0.74–3.76	+0.50 (−0.17 to 1.18)	9.9%
45-49 years	3.77	6.53	1.73	0.82–4.09	+1.77* (1.08 to 2.47)	10.5%
50-54 years	6.91	9.45	1.37	0.62–3.25	+1.51* (0.93 to 2.10)	11.4%
55-59 years	8.17	13.16	1.61	0.76–3.26	+1.80* (1.45 to 2.15)	11.4%
60-64 years	14.63	21.87	1.50	0.73–3.12	+1.32* (1.13 to 1.52)	12.5%
65-69 years	14.24	27.91	1.96	0.99–4.08	+2.23* (1.95 to 2.50)	9.0%
70-74 years	21.81	40.62	1.86	0.88–3.95	+3.33* (2.59 to 4.07)	7.3%
75-79 years	23.07	56.45	2.45	1.10–5.64	+5.20* (4.32 to 6.08)	6.4%
80-84 years	47.25	105.07	2.22	0.87–7.54	+3.29* (2.70 to 3.88)	5.5%
85+ years	53.08	109.18	2.06	0.82–6.11	+2.03* (1.80 to 2.26)	2.5%
DALYs – Age-Specific Rates per 100,000:						
15-19 years	6.9	8.6	1.25	0.50–3.30	+0.06 (−0.15 to 0.27)	0.4%
20-24 years	8.6	11.9	1.39	0.57–3.22	−0.01 (−0.64 to 0.61)	0.7%
25-29 years	22.3	28.8	1.30	0.55–2.94	−0.27 (−1.26 to 0.72)	2.5%
30-34 years	54.1	78.4	1.45	0.66–3.71	−0.07 (−0.91 to 0.78)	7.3%
35-39 years	89.6	135.1	1.51	0.67–3.67	+0.02 (−0.77 to 0.82)	11.2%
40-44 years	136.3	218.9	1.61	0.77–3.84	+0.59 (−0.08 to 1.27)	13.9%
45-49 years	168.3	298.6	1.77	0.86–4.18	+1.85* (1.17 to 2.54)	13.1%
50-54 years	272.9	382.5	1.40	0.65–3.30	+1.60* (1.01 to 2.19)	12.7%
55-59 years	284.1	466.9	1.64	0.78–3.31	+1.88* (1.52 to 2.23)	11.1%
60-64 years	438.9	673.7	1.53	0.75–3.20	+1.42* (1.23 to 1.61)	10.6%
65-69 years	362.5	727.5	2.01	1.01–4.15	+2.31* (2.04 to 2.59)	6.5%
70-74 years	455.6	870.2	1.91	0.92–3.98	+3.39* (2.66 to 4.14)	4.3%
75-79 years	391.1	969.5	2.48	1.13–5.42	+5.23* (4.36 to 6.11)	3.0%
80-84 years	626.4	1392.4	2.22	0.89–7.46	+3.29* (2.70 to 3.88)	2.0%
85+ years	508.7	1045.2	2.05	0.82–5.93	+2.08* (1.84 to 2.32)	0.7%
Peak Age and Age Distribution Summary:						
Measure	Year	Peak Age	Peak Rate	Mean Age	<50 years (%)	≥50 years (%)
Incidence	1990	80-84 years	49.48	76.2	42.9%	57.1%
	2023	80-84 years	159.03	75.4	47.6%	52.4%
Mortality	1990	95+ years	65.89	82.3	32.2%	67.8%
	2023	95+ years	123.21	83.1	33.9%	66.1%
DALYs	1990	80-84 years	626.36	73.6	48.1%	51.9%
	2023	80-84 years	1392.38	75.4	49.1%	50.9%
Cumulative Burden by Age Threshold (2023):						
Measure	By Age 40	By Age 50	By Age 60	By Age 70	By Age 80	Median Age
**Incidence**	10.3%	47.6%	70.1%	88.5%	96.7%	~52 years
**Mortality**	6.2%	33.9%	56.6%	78.2%	91.9%	~58 years
**DALYs**	10.9%	49.1%	72.9%	90.0%	97.3%	~51 years

* Notes: Statistically Significant at P-value < 0.05. Rate ratio compares 2023 to 1990 (reference). Mean age is rate-weighted. Young defined as <50 years; older as ≥50 years. Median age is the age by which 50% of cumulative burden is reached. Abbreviations: APC, Annual Percent Change; CI, Confidence Interval; DALYs, Disability-Adjusted Life-Years; UI, Uncertainty Interval.

**Table 6:** Mixed Effects Regional and Global Comparative Analysis For Breast Cancer In Saudi Arabia.

Parameter	Incidence ASR	Mortality ASR	DALY Rate ASR	Unit/Notes
Temporal Trend Analysis (Saudi Arabia, Females):				
Study period	1990–2023	1980–2023	1990–2023	Years
Number of years	34	44	34	n
Number of periods	7	9	7	5-year periods
Annual percent change	+3.96%	+1.86%	+2.05%	APC
APC 95% CI	3.66 to 4.27	1.59 to 2.12	1.73 to 2.37	—
P-value (trend)	<0.001	<0.001	<0.001	—
R^2^ (fixed effects)	0.9544	0.8190	0.8327	Variance explained
τ^2^ (between-period)	0.0059	0.0102	0.0068	Random effects variance
τ (SD)	0.077	0.101	0.083	√τ^2^
Region	Inc ASR (SE)	Mort ASR (SE)	DALY ASR (SE)	SDI
Regional Comparison (GBD 2019):				
Global	47.8 (1.2)	13.6 (0.4)	387.5 (12.1)	0.67
North Africa and Middle East	38.2 (2.1)	14.2 (0.8)	412.3 (18.5)	0.62
Saudi Arabia	43.7 (3.5)	17.2 (1.2)	494.9 (28.4)	0.75
High-income	74.4 (1.8)	13.1 (0.5)	321.6 (10.2)	0.89
High-middle SDI	42.8 (2.2)	12.8 (0.6)	342.1 (14.3)	0.73
Middle SDI	28.4 (1.5)	11.9 (0.5)	298.4 (11.8)	0.60
Low-middle SDI	23.1 (1.4)	10.8 (0.5)	285.7 (12.4)	0.42
Low SDI	19.8 (1.8)	12.4 (0.7)	328.2 (16.8)	0.24
Comparison	Incidence	Mortality	DALYs	Interpretation
Saudi Arabia Relative Position:				
vs Global	−8.6%	+26.5%	+27.7%	Below/Above global
vs MENA region	+14.4%	+21.1%	+20.0%	Above regional
vs High-income	−41.3%	+31.3%	+53.9%	Lower inc, higher mort
vs High-middle SDI	+2.1%	+34.4%	+44.7%	Similar inc, higher mort
Rank (of 8 regions)	4th	1st (highest)	1st (highest)	—
SDI Model Parameter	Estimate	SE	95% CI	Interpretation
SDI-Incidence Mixed Model:				
Intercept (β_0_)	−8.97	10.15	−28.9 to 10.9	ASR at SDI=0
SDI slope (β_1_)	80.53	16.39	48.4 to 112.7	ASR per 1.0 SDI
Model R^2^	0.8010	—	—	Variance explained
Saudi Arabia expected	51.4	—	—	At SDI=0.75
Saudi Arabia observed	43.7	—	—	Actual value
Saudi Arabia residual	−7.7	—	−15.0%	Below expected
Heterogeneity Statistic	Incidence	Mortality	DALYs	Interpretation
Heterogeneity Analysis:				
Number of regions (k)	8	8	8	—
Cochran’s Q	738.0	40.4	95.4	Chi-square statistic
Degrees of freedom	7	7	7	k – 1
P-value (Q)	<0.001	<0.001	<0.001	Significant heterogeneity
I^2^ statistic	99.1%	82.7%	92.7%	% variance due to heterogeneity
τ^2^ (between-region)	312.57	1.57	2443.11	Random effects variance
τ (SD)	17.68	1.25	49.43	per 100,000
H^2^ statistic	105.43	5.77	13.63	Relative excess variance
H statistic	10.27	2.40	3.69	√H^2^
Pooled Estimate	Incidence	Mortality	DALYs	Method
Random Effects Meta-Analysis:				
Pooled estimate	39.8	13.0	354.9	per 100,000
Pooled SE	6.3	0.5	18.4	—
95% CI lower	27.4	12.1	318.9	—
95% CI upper	52.1	14.0	390.9	—
Saudi Arabia vs pooled	+9.8%	+32.3%	+39.5%	Relative difference
Model Specification	Temporal	Regional	SDI	Software
LimeTr Model Specifications:				
Model type	Linear mixed	Random effects	WLS regression	LimeTr/Python
Fixed effects	Intercept + time	Intercept only	Intercept + SDI	—
Random effects	Period (5-year)	Region	None	—
Variance estimation	REML-like	DerSimonian-Laird	OLS	—
Weighting	Inverse variance	Inverse variance	Inverse variance	—
Outcome transformation	Log(ASR)	None	None	—
Period	Years	Inc RE	Mort RE	Interpretation
Period Random Effects (Saudi Arabia):				
Period 1	1990–1994	−0.042	−0.038	Below trend
Period 2	1995–1999	−0.028	−0.025	Below trend
Period 3	2000–2004	+0.015	+0.018	At trend
Period 4	2005–2009	+0.089	+0.072	Above trend
Period 5	2010–2014	+0.045	+0.035	Above trend
Period 6	2015–2019	−0.032	−0.028	Below trend
Period 7	2020–2023	−0.047	−0.034	Below trend

Abbreviations: APC, Annual Percent Change; ASR, Age-Standardized Rate; CI, Confidence Interval; DALY, Disability-Adjusted Life Year; df, Degrees Of Freedom; GBD, Global Burden of Disease; H^2^, Relative Excess Heterogeneity; I^2^, Percentage Of Variance Due To Heterogeneity; Inc, Incidence; k, Number of Studies/Regions; LimeTr, Linear Mixed Effects With Trimming; MENA, Middle East and North Africa; Mort, Mortality; n, Number; OLS, Ordinary Least Squares; Q, Cochran’s Q Statistic; R^2^, Coefficient of Determination; RE, Random Effect; REML, Restricted Maximum Likelihood; SD, Standard Deviation; SDI, Socio-Demographic Index; SE, Standard Error; τ^2^, Between-Group Variance; vs, Versus; WLS, Weighted Least Squares.
