# Supplementary material for: National Burden of Breast Cancer in Saudi Arabia, 1990–2023, With Forecasts to 2050: A Systematic Analysis for the Global Burden of Disease Study 2023
Source: Evidance Health Sci. Author manuscript; Available in PMC 2026 May 7. (PMC13148422; doi:10.65416/ehealthsci.2026.117757)
Supplement: Appendix — Supplementary Figure 1: Joinpoint Regression Analysis of Incidence and Mortality Trends. Supplementary Figure 2: Lee-Carter Model Mortality Forecast To 2050. Supplementary Figure 3: Bayesian Age-Period-Cohort Variance Decomposition. Supplementary Figure 4: Compression Versus Expansion of Morbidity Analysis. Table 1: Annual Time Series of Breast Cancer Burden In Saudi Arabia, 1990–2023. Supplementary Table 2: Sex-Specific Annual Time Series of Breast Cancer Burden In Saudi Arabia, 1990–2023. Supplementary Table 3: Annual Time Series of YLLs, YLDs, and Prevalence For Breast Cancer In Saudi Arabia, 1990–2023. Supplementary Table 4: Detailed Statistical Analysis and Sensitivity Assessment of Breast Cancer Trends In Saudi Arabia, 1990–2023. [file NIHMS2163534-supplement-Appendix.zip › Supplementary Table 9.docx]

**Supplementary Table 9:** Risk Factor Attribution, Burden of Proof, and Meta-Regression.

| **Risk Factor** | **GBD ID** | **Category** | **Exposure Unit** | **Value** |
| --- | --- | --- | --- | --- |
| **MR-BRT Meta-Regression:** | | | | |
| High BMI (≥25 kg/m²) | rei_id(108) | Metabolic | per 5 kg/m² | — |
| Pooled log(RR) | — | — | — | 0.1085 |
| Pooled RR | — | — | — | 1.115 |
| Number of studies | — | — | — | 4 |
| Between-study τ² | — | — | — | <0.001 |
| Alcohol use | rei_id(102) | Behavioral | per 10g/day | — |
| Pooled log(RR) | — | — | — | 0.1060 |
| Pooled RR | — | — | — | 1.112 |
| Number of studies | — | — | — | 4 |
| Low physical activity | rei_id(125) | Behavioral | low vs adequate | — |
| Pooled log(RR) | — | — | — | 0.0811 |
| Pooled RR | — | — | — | 1.085 |
| Number of studies | — | — | — | 4 |
| High fasting plasma glucose | rei_id(105) | Metabolic | per mmol/L | — |
| Pooled log(RR) | — | — | — | 0.0500 |
| Pooled RR | — | — | — | 1.051 |
| Number of studies | — | — | — | 3 |
| Tobacco/Smoking | rei_id(99) | Behavioral | ever vs never | — |
| Pooled log(RR) | — | — | — | 0.0826 |
| Pooled RR | — | — | — | 1.086 |
| Number of studies | — | — | — | 4 |
| **Risk Factor** | **RR (95% CI)** | **BPRF** | **RBS** | **Evidence Rating** |
| **Burden of Proof Analysis:** | | | | |
| High BMI (≥25 kg/m²) | 1.115 (1.082–1.148) | 1.087 | 7.23 | ★★★★★ |
| Alcohol use | 1.112 (1.080–1.145) | 1.085 | 7.07 | ★★★★★ |
| Low physical activity | 1.084 (1.043–1.128) | 1.049 | 4.06 | ★★★★ |
| High fasting plasma glucose | 1.051 (1.021–1.083) | 1.026 | 3.33 | ★★★ |
| Tobacco/Smoking | 1.086 (1.048–1.125) | 1.054 | 4.59 | ★★★★ |
| **Evidence Rating Scale:** | | | | |
| ★★★★★ (5 stars) | RBS ≥ 5 | — | Very strong | Convincing evidence |
| ★★★★ (4 stars) | RBS ≥ 4 | — | Strong | Highly probable |
| ★★★ (3 stars) | RBS ≥ 3 | — | Moderate | Probable association |
| ★★ (2 stars) | RBS ≥ 2 | — | Weak | Possible association |
| ★ (1 star) | RBS ≥ 1 | — | Very weak | Suggestive only |
| **Risk Factor** | **Prevalence** | **RR** | **PAF** | **Attributable %** |
| **Population Attributable Fraction (Saudi Arabia):** | | | | |
| High BMI (≥25 kg/m²) | 0.70 | 1.115 | 0.0743 | 7.43% |
| Alcohol use | 0.02 | 1.112 | 0.0022 | 0.22% |
| Low physical activity | 0.45 | 1.084 | 0.0366 | 3.66% |
| High fasting plasma glucose | 0.25 | 1.051 | 0.0127 | 1.27% |
| Tobacco/Smoking | 0.05 | 1.086 | 0.0043 | 0.43% |
| Combined (all risk factors) | — | — | 0.1252 | 12.52% |
| **Risk Factor** | **Attrib. Incidence** | **Attrib. Deaths** | **Attrib. DALYs** | **Contribution %** |
| **Attributable Burden, Females 2023:** | | | | |
| High BMI (≥25 kg/m²) | 299 | 85 | 3,110 | 59.3% |
| Alcohol use | 9 | 3 | 92 | 1.8% |
| Low physical activity | 147 | 42 | 1,532 | 29.2% |
| High fasting plasma glucose | 51 | 15 | 531 | 10.1% |
| Tobacco/Smoking | 17 | 5 | 180 | 3.4% |
| Combined (non-additive) | 504 | 144 | 5,240 | — |
| **Reference: Total Burden 2023:** | | | | |
| Total incidence, n | 4,025 | — | — | — |
| Total deaths, n | 1,149 | — | — | — |
| Total DALYs | 41,855 | — | — | — |
| Attributable fraction | 12.52% | — | — | Preventable |
| **Model Parameter** | **Specification** | **Value** | **Method** | **Reference** |
| **MR-BRT Model Specifications:** | | | | |
| Model type | MR-BRT | — | Meta-regression | IHME/mrtool |
| Covariate model | LinearCovModel | intercept | Random effects | — |
| Prior distribution | Gaussian | — | Regularized | — |
| Trimming | None applied | — | All studies included | — |
| Heterogeneity model | Random effects | τ² estimated | DerSimonian-Laird | — |
| **BPRF Methodology:** | | | | |
| BPRF calculation | exp(log_RR − 1.645 × SE) | — | One-sided 95% CI | bopforge |
| RBS calculation | log_RR / SE | — | Standardized effect | GBD CRA |
| Evidence threshold | BPRF > 1.0 | — | Harmful effect | — |
| Star rating system | RBS-based | 1–5 stars | GBD convention | — |
| **PAF Methodology:** | | | | |
| PAF formula | p(RR−1) / [p(RR−1)+1] | — | Levin's formula | — |
| Combined PAF | 1 − Π(1−PAFᵢ) | — | Multiplicative | — |
| Exposure data source | GBD 2019 | Saudi Arabia | Population prevalence | — |
| Uncertainty propagation | Not applied | — | Point estimates | — |
| **Risk Factor** | **Prevalence** | **Year** | **Population** | **Source** |
| **Exposure Prevalence (Saudi Arabia):** | | | | |
| Overweight/obesity (BMI ≥25) | 70% | 2019 | Adult females | GBD 2019 |
| Alcohol consumption | 2% | 2019 | Adult females | GBD 2019 |
| Low physical activity | 45% | 2019 | Adult females | GBD 2019 |
| High FPG (≥5.6 mmol/L) | 25% | 2019 | Adult females | GBD 2019 |
| Current/former smoking | 5% | 2019 | Adult females | GBD 2019 |

***Abbreviations:*** *BMI, body-mass index; BPRF, burden of proof risk function; CI, confidence interval; CRA, Comparative Risk Assessment; DALY, disability-adjusted life year; FPG, fasting plasma glucose; GBD, Global Burden of Disease; IHME, Institute for Health Metrics and Evaluation; MR-BRT, meta-regression—Bayesian, regularized, trimmed; n, number; PAF, population attributable fraction; RBS, risk-burden score; RR, relative risk; SE, standard error; τ², between-study variance.*
