# Supplementary material for: National Burden of Breast Cancer in Saudi Arabia, 1990–2023, With Forecasts to 2050: A Systematic Analysis for the Global Burden of Disease Study 2023
Source: Evidance Health Sci. Author manuscript; Available in PMC 2026 May 7. (PMC13148422; doi:10.65416/ehealthsci.2026.117757)
Supplement: Appendix — Supplementary Figure 1: Joinpoint Regression Analysis of Incidence and Mortality Trends. Supplementary Figure 2: Lee-Carter Model Mortality Forecast To 2050. Supplementary Figure 3: Bayesian Age-Period-Cohort Variance Decomposition. Supplementary Figure 4: Compression Versus Expansion of Morbidity Analysis. Table 1: Annual Time Series of Breast Cancer Burden In Saudi Arabia, 1990–2023. Supplementary Table 2: Sex-Specific Annual Time Series of Breast Cancer Burden In Saudi Arabia, 1990–2023. Supplementary Table 3: Annual Time Series of YLLs, YLDs, and Prevalence For Breast Cancer In Saudi Arabia, 1990–2023. Supplementary Table 4: Detailed Statistical Analysis and Sensitivity Assessment of Breast Cancer Trends In Saudi Arabia, 1990–2023. [file NIHMS2163534-supplement-Appendix.zip › Supplementary Figure 1.pdf]

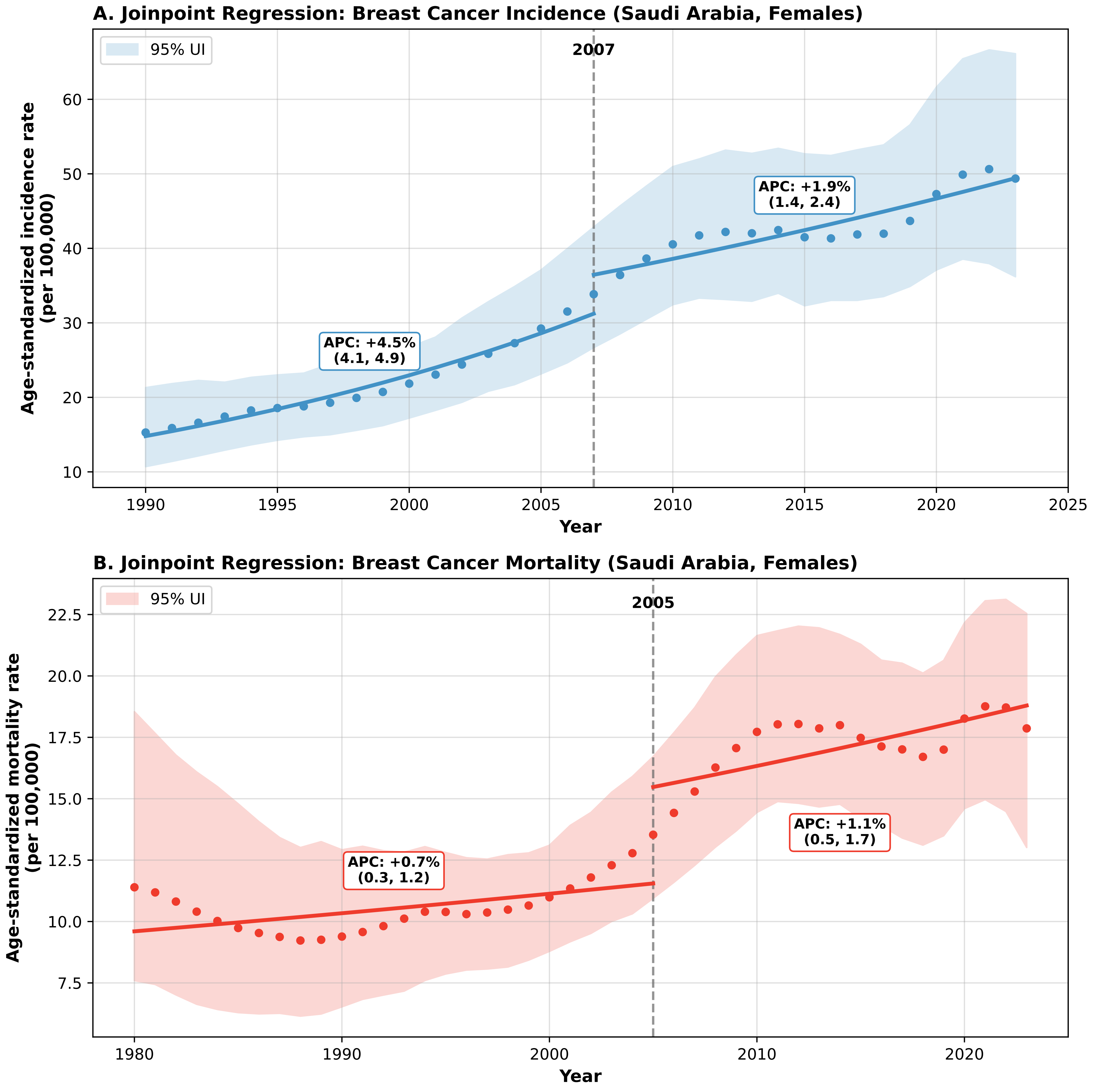
A. Joinpoint Regression: Breast Cancer Incidence (Saudi Arabia, Females)
95% UI
2007
APC: +4.5%
(4.1, 4.9)
APC: +1.9%
(1.4, 2.4)
Age-standardized incidence rate
(per 100,000)
Year
10
20
30
40
50
60
1990
1995
2000
2005
2010
2015
2020
2025
B. Joinpoint Regression: Breast Cancer Mortality (Saudi Arabia, Females)
95% UI
2005
APC: +0.7%
(0.3, 1.2)
APC: +1.1%
(0.5, 1.7)
Age-standardized mortality rate
(per 100,000)
Year
7.5
10.0
12.5
15.0
17.5
20.0
22.5
1980
1990
2000
2010
2020
