# Supplementary material for: National Burden of Breast Cancer in Saudi Arabia, 1990–2023, With Forecasts to 2050: A Systematic Analysis for the Global Burden of Disease Study 2023
Source: Evidance Health Sci. Author manuscript; Available in PMC 2026 May 7. (PMC13148422; doi:10.65416/ehealthsci.2026.117757)
Supplement: Appendix — Supplementary Figure 1: Joinpoint Regression Analysis of Incidence and Mortality Trends. Supplementary Figure 2: Lee-Carter Model Mortality Forecast To 2050. Supplementary Figure 3: Bayesian Age-Period-Cohort Variance Decomposition. Supplementary Figure 4: Compression Versus Expansion of Morbidity Analysis. Table 1: Annual Time Series of Breast Cancer Burden In Saudi Arabia, 1990–2023. Supplementary Table 2: Sex-Specific Annual Time Series of Breast Cancer Burden In Saudi Arabia, 1990–2023. Supplementary Table 3: Annual Time Series of YLLs, YLDs, and Prevalence For Breast Cancer In Saudi Arabia, 1990–2023. Supplementary Table 4: Detailed Statistical Analysis and Sensitivity Assessment of Breast Cancer Trends In Saudi Arabia, 1990–2023. [file NIHMS2163534-supplement-Appendix.zip › Supplementary Figure 2.pdf]

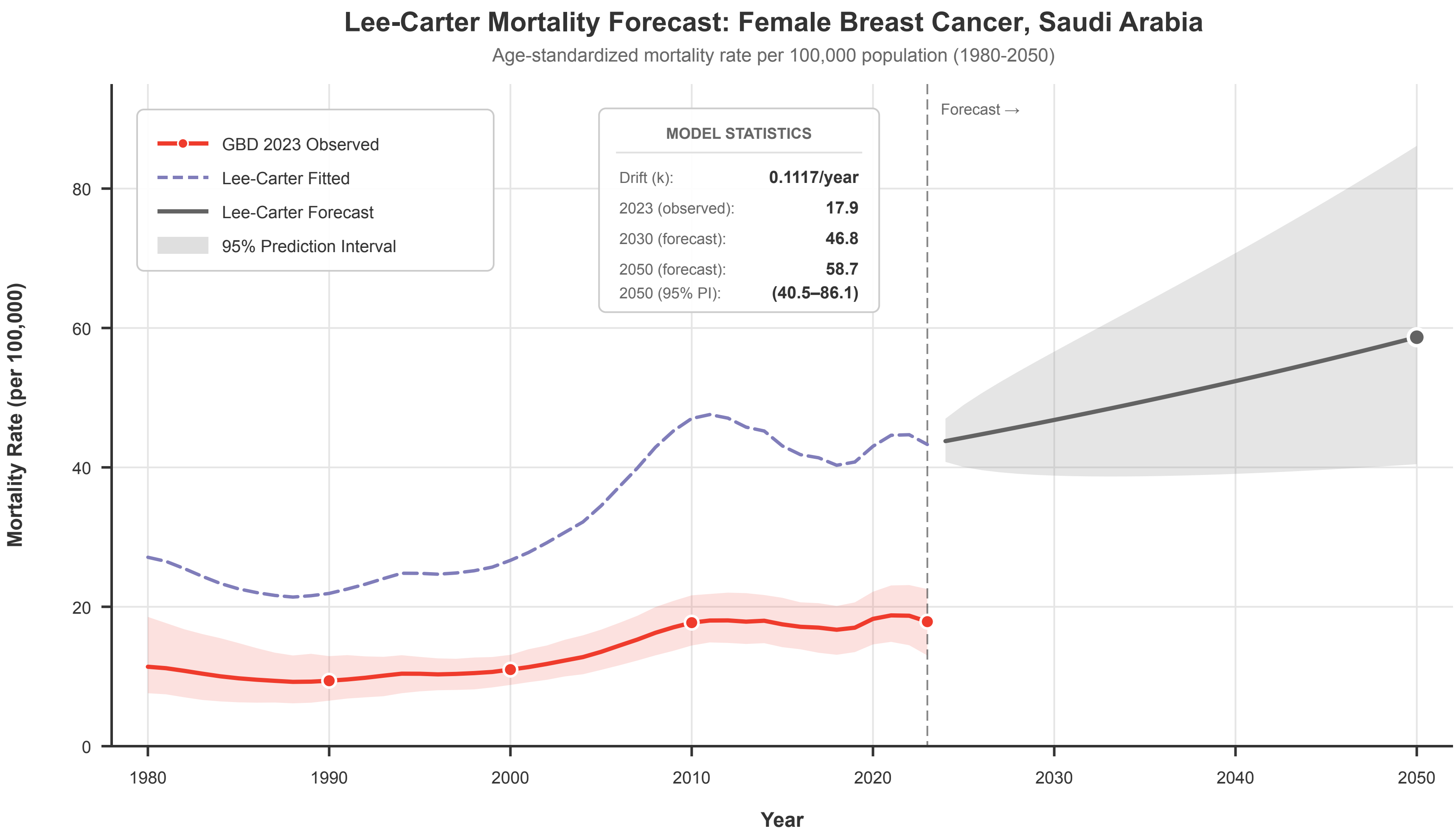

Lee-Carter Mortality Forecast: Female Breast Cancer, Saudi Arabia
Age-standardized mortality rate per 100,000 population (1980-2050)
GBD 2023 Observed
Lee-Carter Fitted
Lee-Carter Forecast
95% Prediction Interval
MODEL STATISTICS
Drift (k): 0.1117/year
2023 (observed): 17.9
2030 (forecast): 46.8
2050 (forecast): 58.7
2050 (95% PI): (40.5–86.1)
Forecast →
Mortality Rate (per 100,000)
0
20
40
60
80
1980
1990
2000
2010
2020
2030
2040
2050
Year
