# Supplementary material for: National Burden of Breast Cancer in Saudi Arabia, 1990–2023, With Forecasts to 2050: A Systematic Analysis for the Global Burden of Disease Study 2023
Source: Evidance Health Sci. Author manuscript; Available in PMC 2026 May 7. (PMC13148422; doi:10.65416/ehealthsci.2026.117757)
Supplement: Appendix — Supplementary Figure 1: Joinpoint Regression Analysis of Incidence and Mortality Trends. Supplementary Figure 2: Lee-Carter Model Mortality Forecast To 2050. Supplementary Figure 3: Bayesian Age-Period-Cohort Variance Decomposition. Supplementary Figure 4: Compression Versus Expansion of Morbidity Analysis. Table 1: Annual Time Series of Breast Cancer Burden In Saudi Arabia, 1990–2023. Supplementary Table 2: Sex-Specific Annual Time Series of Breast Cancer Burden In Saudi Arabia, 1990–2023. Supplementary Table 3: Annual Time Series of YLLs, YLDs, and Prevalence For Breast Cancer In Saudi Arabia, 1990–2023. Supplementary Table 4: Detailed Statistical Analysis and Sensitivity Assessment of Breast Cancer Trends In Saudi Arabia, 1990–2023. [file NIHMS2163534-supplement-Appendix.zip › Supplementary Figure 3.pdf]

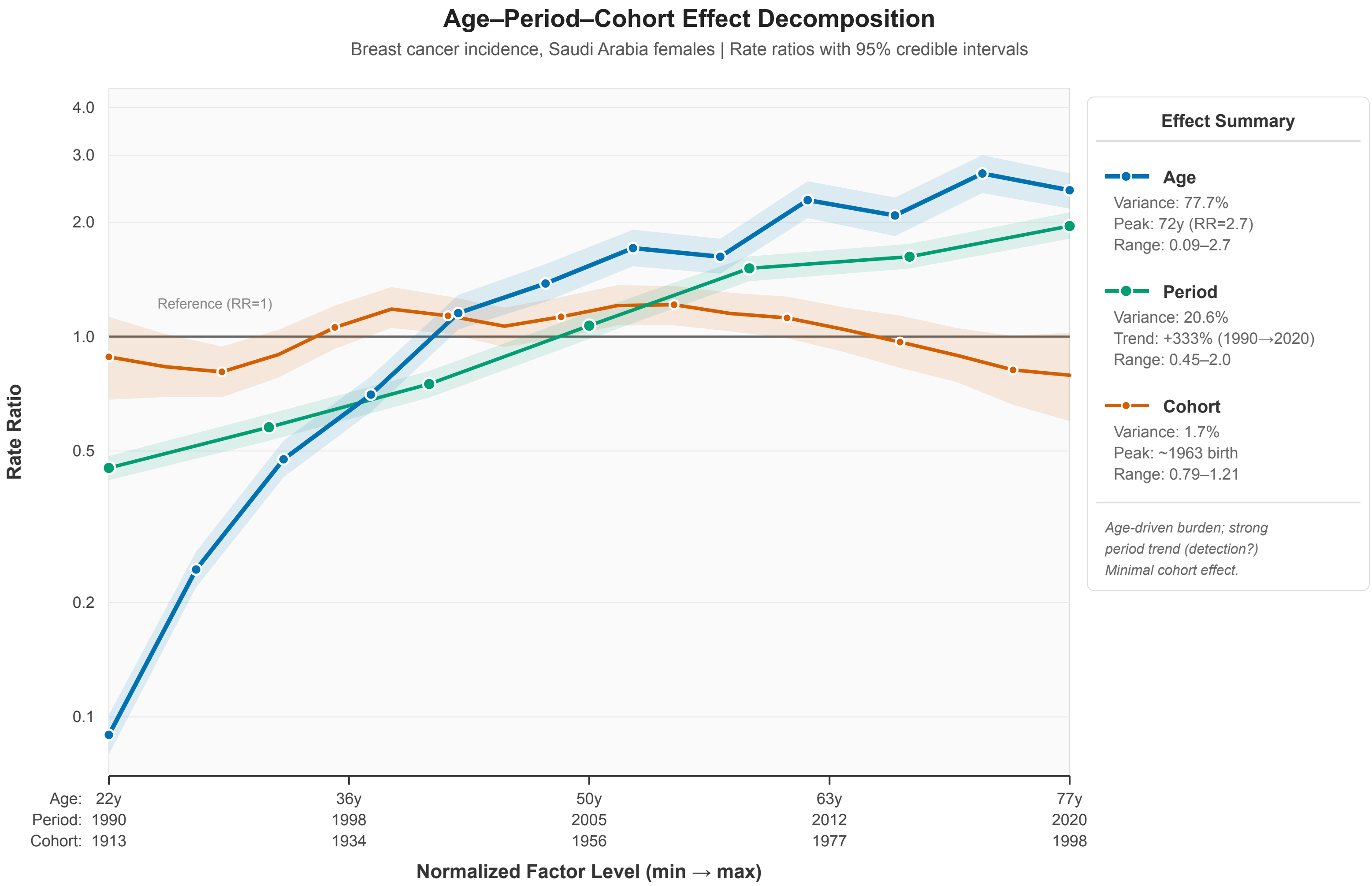
Age–Period–Cohort Effect Decomposition
Breast cancer incidence, Saudi Arabia females | Rate ratios with 95% credible intervals
Rate Ratio
4.0
3.0
2.0
1.0
0.5
0.2
0.1
Reference (RR=1)
Age: 22y
Period: 1990
Cohort: 1913
36y
1998
1934
50y
2005
1956
63y
2012
1977
77y
2020
1998
Normalized Factor Level (min → max)
Effect Summary
Age
Variance: 77.7%
Peak: 72y (RR=2.7)
Range: 0.09–2.7
Period
Variance: 20.6%
Trend: +333% (1990→2020)
Range: 0.45–2.0
Cohort
Variance: 1.7%
Peak: ~1963 birth
Range: 0.79–1.21
Age-driven burden; strong
period trend (detection?)
Minimal cohort effect.
