# Supplementary material for: National Burden of Breast Cancer in Saudi Arabia, 1990–2023, With Forecasts to 2050: A Systematic Analysis for the Global Burden of Disease Study 2023
Source: Evidance Health Sci. Author manuscript; Available in PMC 2026 May 7. (PMC13148422; doi:10.65416/ehealthsci.2026.117757)
Supplement: Appendix — Supplementary Figure 1: Joinpoint Regression Analysis of Incidence and Mortality Trends. Supplementary Figure 2: Lee-Carter Model Mortality Forecast To 2050. Supplementary Figure 3: Bayesian Age-Period-Cohort Variance Decomposition. Supplementary Figure 4: Compression Versus Expansion of Morbidity Analysis. Table 1: Annual Time Series of Breast Cancer Burden In Saudi Arabia, 1990–2023. Supplementary Table 2: Sex-Specific Annual Time Series of Breast Cancer Burden In Saudi Arabia, 1990–2023. Supplementary Table 3: Annual Time Series of YLLs, YLDs, and Prevalence For Breast Cancer In Saudi Arabia, 1990–2023. Supplementary Table 4: Detailed Statistical Analysis and Sensitivity Assessment of Breast Cancer Trends In Saudi Arabia, 1990–2023. [file NIHMS2163534-supplement-Appendix.zip › Supplementary Figure 4.pdf]

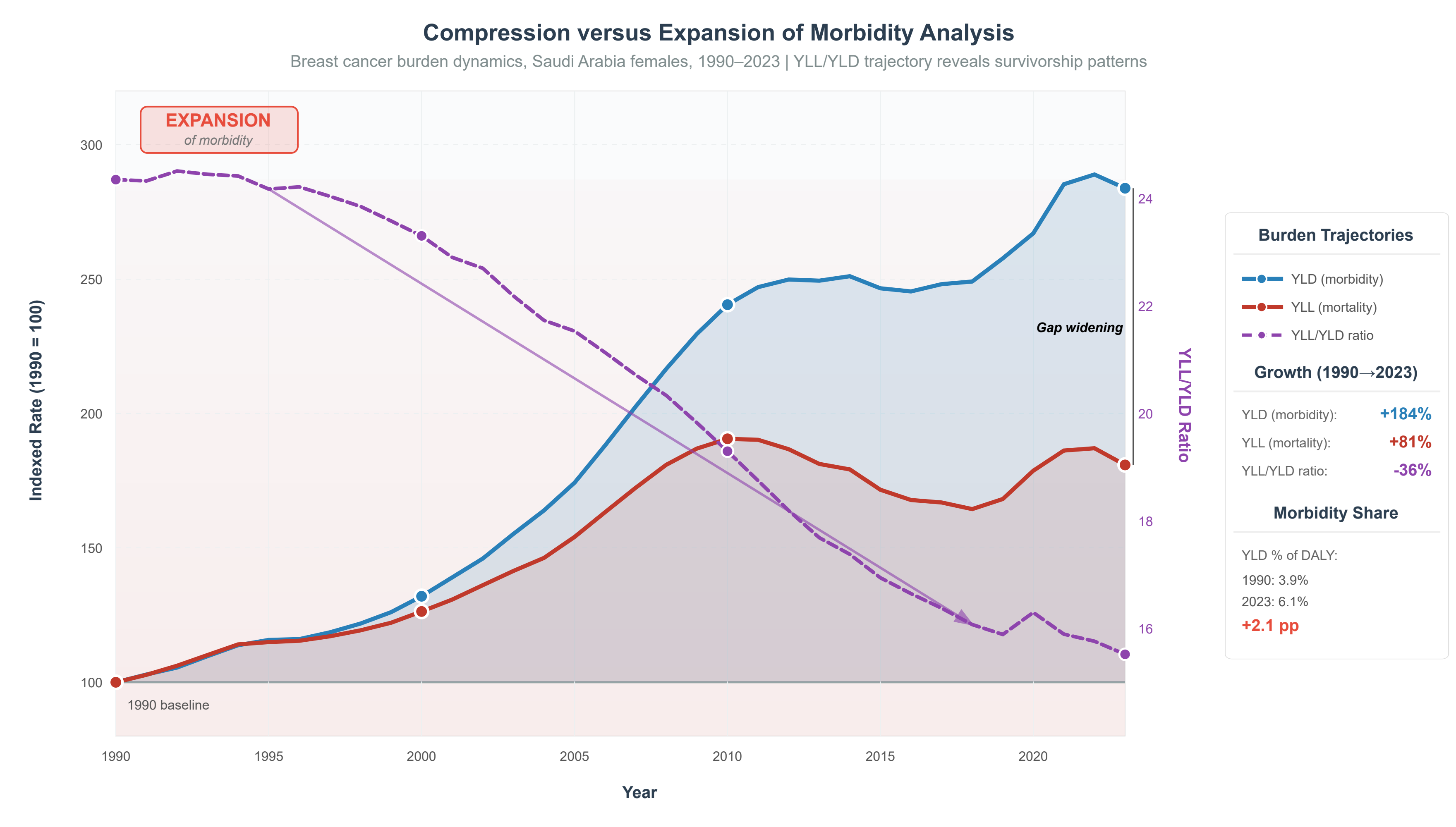

Compression versus Expansion of Morbidity Analysis
Breast cancer burden dynamics, Saudi Arabia females, 1990–2023 | YLL/YLD trajectory reveals survivorship patterns
EXPANSION
of morbidity
Indexed Rate (1990 = 100)
300
250
200
150
100
1990 baseline
Gap widening
24
22
20
18
16
YLL/YLD Ratio
1990
1995
2000
2005
2010
2015
2020
Year
Burden Trajectories
YLD (morbidity)
YLL (mortality)
YLL/YLD ratio
Growth (1990→2023)
YLD (morbidity): +184%
YLL (mortality): +81%
YLL/YLD ratio: -36%
Morbidity Share
YLD % of DALY:
1990: 3.9%
2023: 6.1%
+2.1 pp
