# Supplementary material for: National Burden of Breast Cancer in Saudi Arabia, 1990–2023, With Forecasts to 2050: A Systematic Analysis for the Global Burden of Disease Study 2023
Source: Evidance Health Sci. Author manuscript; Available in PMC 2026 May 7. (PMC13148422; doi:10.65416/ehealthsci.2026.117757)
Supplement: Appendix — Supplementary Figure 1: Joinpoint Regression Analysis of Incidence and Mortality Trends. Supplementary Figure 2: Lee-Carter Model Mortality Forecast To 2050. Supplementary Figure 3: Bayesian Age-Period-Cohort Variance Decomposition. Supplementary Figure 4: Compression Versus Expansion of Morbidity Analysis. Table 1: Annual Time Series of Breast Cancer Burden In Saudi Arabia, 1990–2023. Supplementary Table 2: Sex-Specific Annual Time Series of Breast Cancer Burden In Saudi Arabia, 1990–2023. Supplementary Table 3: Annual Time Series of YLLs, YLDs, and Prevalence For Breast Cancer In Saudi Arabia, 1990–2023. Supplementary Table 4: Detailed Statistical Analysis and Sensitivity Assessment of Breast Cancer Trends In Saudi Arabia, 1990–2023. [file NIHMS2163534-supplement-Appendix.zip › Supplementary Table 1.docx]

**Supplementary Table 1:** Annual Time Series of Breast Cancer Burden In Saudi Arabia, 1990–2023.

| **Year** | **Incidence** | | **Mortality** | | **DALYs** | | **MIR** |
| --- | --- | --- | --- | --- | --- | --- | --- |
|  | **Cases (95% UI)** | **ASR (95% UI)** | **Deaths (95% UI)** | **ASR (95% UI)** | **Number (95% UI)** | **ASR (95% UI)** |  |
| **1990** | 454 (322–630) | 6.47 (4.56–8.94) | 243 (172–334) | 4.12 (2.86–5.62) | 8,524 (6,144–12,000) | 111.58 (79.32–151.55) | 0.535 |
| 1991 | 500 (361–680) | 6.71 (4.82–9.09) | 261 (191–357) | 4.19 (3.02–5.63) | 9,282 (6,882–12,650) | 114.53 (83.66–154.87) | 0.522 |
| 1992 | 554 (405–735) | 7.01 (5.14–9.19) | 282 (211–369) | 4.30 (3.10–5.56) | 10,150 (7,657–13,230) | 118.21 (88.12–153.73) | 0.509 |
| 1993 | 616 (464–797) | 7.36 (5.40–9.30) | 307 (234–390) | 4.43 (3.17–5.58) | 11,147 (8,527–14,382) | 122.73 (94.05–155.78) | 0.498 |
| 1994 | 683 (517–861) | 7.71 (5.78–9.60) | 334 (259–414) | 4.56 (3.38–5.69) | 12,215 (9,463–15,339) | 127.18 (98.20–159.06) | 0.489 |
| 1995 | 737 (565–914) | 7.85 (5.98–9.63) | 352 (275–433) | 4.57 (3.47–5.62) | 13,041 (10,167–16,245) | 128.32 (100.93–158.35) | 0.478 |
| 1996 | 793 (629–988) | 7.95 (6.24–9.77) | 371 (295–455) | 4.54 (3.55–5.53) | 13,898 (11,039–17,113) | 128.98 (101.89–157.09) | 0.468 |
| 1997 | 859 (676–1,106) | 8.18 (6.44–10.20) | 394 (317–481) | 4.58 (3.60–5.54) | 14,860 (12,086–18,341) | 131.32 (105.89–158.71) | 0.459 |
| 1998 | 935 (731–1,190) | 8.48 (6.68–10.67) | 420 (340–507) | 4.65 (3.61–5.61) | 15,903 (13,095–19,593) | 134.21 (109.35–161.20) | 0.449 |
| 1999 | 1,022 (798–1,296) | 8.83 (7.02–10.85) | 448 (354–540) | 4.73 (3.73–5.63) | 17,063 (13,690–20,738) | 137.65 (111.49–165.59) | 0.438 |
| 2000 | 1,124 (872–1,407) | 9.30 (7.38–11.39) | 483 (384–579) | 4.88 (3.89–5.79) | 18,390 (14,505–22,382) | 142.45 (114.14–170.72) | 0.430 |
| 2001 | 1,236 (978–1,522) | 9.84 (7.86–11.99) | 520 (415–635) | 5.05 (4.11–6.12) | 19,811 (15,819–24,065) | 148.10 (120.04–179.78) | 0.421 |
| 2002 | 1,361 (1,070–1,717) | 10.44 (8.32–13.05) | 562 (456–692) | 5.27 (4.28–6.45) | 21,435 (17,263–26,470) | 154.73 (125.90–190.34) | 0.413 |
| 2003 | 1,492 (1,182–1,914) | 11.08 (8.92–14.13) | 607 (491–751) | 5.50 (4.50–6.79) | 23,051 (18,567–28,695) | 161.35 (132.26–198.94) | 0.407 |
| 2004 | 1,628 (1,282–2,076) | 11.71 (9.38–14.93) | 651 (522–803) | 5.72 (4.68–7.09) | 24,678 (19,742–30,835) | 167.36 (135.22–206.31) | 0.400 |
| 2005 | 1,810 (1,438–2,254) | 12.53 (10.03–15.61) | 711 (572–869) | 6.04 (4.93–7.44) | 26,996 (21,623–33,754) | 176.14 (141.46–216.08) | 0.393 |
| 2006 | 2,023 (1,598–2,516) | 13.50 (10.60–16.88) | 782 (629–964) | 6.43 (5.23–7.91) | 29,703 (23,756–36,949) | 186.82 (149.87–229.20) | 0.387 |
| 2007 | 2,250 (1,782–2,853) | 14.47 (11.47–18.31) | 854 (683–1,049) | 6.80 (5.52–8.34) | 32,518 (25,929–40,418) | 197.05 (158.80–242.09) | 0.380 |
| 2008 | 2,488 (1,945–3,174) | 15.53 (12.24–19.58) | 925 (734–1,152) | 7.21 (5.83–8.89) | 35,147 (27,959–44,305) | 206.64 (167.09–254.57) | 0.372 |
| 2009 | 2,706 (2,110–3,526) | 16.43 (13.07–20.54) | 985 (785–1,226) | 7.54 (6.08–9.21) | 37,340 (29,534–47,665) | 213.33 (171.46–263.29) | 0.364 |
| 2010 | 2,891 (2,291–3,833) | 17.17 (13.78–21.33) | 1,030 (835–1,266) | 7.79 (6.41–9.52) | 38,869 (31,223–50,369) | 217.07 (176.57–266.29) | 0.356 |
| 2011 | 3,018 (2,341–4,016) | 17.68 (14.19–21.70) | 1,051 (852–1,307) | 7.93 (6.55–9.69) | 39,406 (31,503–51,885) | 216.97 (177.69–260.58) | 0.348 |
| 2012 | 3,077 (2,349–4,127) | 17.88 (14.21–22.00) | 1,051 (853–1,317) | 7.95 (6.52–9.65) | 39,077 (30,889–51,875) | 213.34 (174.38–257.63) | 0.342 |
| 2013 | 3,076 (2,342–4,250) | 17.81 (14.02–22.12) | 1,036 (839–1,330) | 7.89 (6.45–9.70) | 38,125 (30,227–51,938) | 207.44 (171.72–253.52) | 0.337 |
| 2014 | 3,113 (2,404–4,337) | 17.99 (14.53–22.20) | 1,041 (839–1,353) | 7.96 (6.48–9.61) | 37,828 (29,978–52,723) | 205.34 (168.45–253.45) | 0.334 |
| 2015 | 3,056 (2,330–4,409) | 17.52 (13.77–21.80) | 1,011 (814–1,358) | 7.70 (6.33–9.23) | 36,435 (28,730–52,683) | 196.23 (159.06–248.00) | 0.331 |
| 2016 | 3,093 (2,411–4,434) | 17.41 (13.92–21.61) | 1,005 (802–1,347) | 7.55 (6.15–9.17) | 36,164 (28,802–51,950) | 191.45 (153.78–244.14) | 0.325 |
| 2017 | 3,189 (2,522–4,461) | 17.56 (14.03–22.15) | 1,017 (826–1,363) | 7.49 (6.01–8.98) | 36,573 (29,594–52,185) | 189.81 (152.82–243.17) | 0.319 |
| 2018 | 3,249 (2,577–4,597) | 17.52 (14.02–21.82) | 1,018 (829–1,369) | 7.34 (5.88–8.81) | 36,558 (29,915–52,487) | 186.18 (149.21–238.59) | 0.313 |
| 2019 | 3,435 (2,708–4,900) | 18.10 (14.42–22.74) | 1,057 (873–1,440) | 7.43 (5.91–9.01) | 37,898 (31,374–54,755) | 189.04 (152.50–244.16) | 0.308 |
| 2020 | 3,694 (2,904–5,219) | 19.46 (15.28–24.29) | 1,129 (927–1,528) | 7.93 (6.35–9.57) | 39,925 (32,716–57,276) | 199.16 (161.64–254.86) | 0.306 |
| 2021 | 3,992 (3,096–5,648) | 20.27 (15.72–25.87) | 1,191 (973–1,606) | 8.07 (6.44–9.67) | 42,583 (34,710–60,566) | 205.09 (165.83–266.71) | 0.298 |
| 2022 | 4,139 (3,128–5,845) | 20.32 (15.38–26.03) | 1,213 (978–1,651) | 7.99 (6.14–9.89) | 43,656 (34,929–63,470) | 203.48 (161.74–264.00) | 0.293 |
| **2023** | 4,168 (3,009–5,990) | 19.58 (14.63–25.80) | 1,197 (901–1,667) | 7.57 (5.65–9.34) | 43,561 (32,964–64,155) | 194.66 (146.84–257.13) | 0.287 |

***Abbreviations:*** *ASR, age-standardized rate; DALYs, disability-adjusted life-years; GBD, Global Burden of Disease; MIR, mortality-to-incidence ratio; UI, uncertainty interval. All estimates are for both sexes combined. ASR = age-standardized rate per 100,000 population. MIR = mortality-to-incidence ratio. 95% UI = 95% uncertainty interval.*
