# Supplementary material for: National Burden of Breast Cancer in Saudi Arabia, 1990–2023, With Forecasts to 2050: A Systematic Analysis for the Global Burden of Disease Study 2023
Source: Evidance Health Sci. Author manuscript; Available in PMC 2026 May 7. (PMC13148422; doi:10.65416/ehealthsci.2026.117757)
Supplement: Appendix — Supplementary Figure 1: Joinpoint Regression Analysis of Incidence and Mortality Trends. Supplementary Figure 2: Lee-Carter Model Mortality Forecast To 2050. Supplementary Figure 3: Bayesian Age-Period-Cohort Variance Decomposition. Supplementary Figure 4: Compression Versus Expansion of Morbidity Analysis. Table 1: Annual Time Series of Breast Cancer Burden In Saudi Arabia, 1990–2023. Supplementary Table 2: Sex-Specific Annual Time Series of Breast Cancer Burden In Saudi Arabia, 1990–2023. Supplementary Table 3: Annual Time Series of YLLs, YLDs, and Prevalence For Breast Cancer In Saudi Arabia, 1990–2023. Supplementary Table 4: Detailed Statistical Analysis and Sensitivity Assessment of Breast Cancer Trends In Saudi Arabia, 1990–2023. [file NIHMS2163534-supplement-Appendix.zip › Supplementary Table 2.docx]

**Supplementary Table 2:** Sex-Specific Annual Time Series of Breast Cancer Burden In Saudi Arabia, 1990–2023.

| **Year** | **Incidence** | | **Mortality** | | **DALYs** | |
| --- | --- | --- | --- | --- | --- | --- |
|  | **Cases (95% UI)** | **ASR (95% UI)** | **Deaths (95% UI)** | **ASR (95% UI)** | **Number (95% UI)** | **ASR (95% UI)** |
| **FEMALE** | | | | | | |
| **1990** | 438 (308–617) | 15.28 (10.70–21.31) | 233 (164–326) | 9.39 (6.53–12.92) | 8,212 (5,844–11,740) | 267.60 (188.02–374.19) |
| 1991 | 483 (348–667) | 15.88 (11.40–21.84) | 251 (183–349) | 9.57 (6.83–13.06) | 8,955 (6,516–12,390) | 275.03 (200.11–379.91) |
| 1992 | 536 (389–723) | 16.60 (12.14–22.27) | 272 (202–361) | 9.81 (7.00–12.88) | 9,811 (7,391–13,010) | 284.06 (209.98–374.02) |
| 1993 | 597 (445–779) | 17.42 (12.91–22.03) | 297 (223–384) | 10.12 (7.17–12.82) | 10,792 (8,315–14,025) | 294.74 (223.58–378.58) |
| 1994 | 664 (501–847) | 18.25 (13.63–22.68) | 323 (248–404) | 10.40 (7.60–13.05) | 11,845 (9,161–14,982) | 305.23 (237.28–386.57) |
| 1995 | 716 (552–895) | 18.56 (14.24–23.01) | 341 (265–419) | 10.39 (7.86–12.81) | 12,645 (9,771–15,826) | 307.63 (239.77–380.15) |
| 1996 | 772 (609–967) | 18.79 (14.71–23.25) | 359 (286–442) | 10.30 (8.02–12.60) | 13,501 (10,736–16,509) | 308.95 (245.09–379.38) |
| 1997 | 837 (654–1,081) | 19.27 (14.98–24.47) | 382 (304–469) | 10.37 (8.07–12.54) | 14,442 (11,721–17,961) | 313.50 (251.24–385.08) |
| 1998 | 911 (709–1,168) | 19.93 (15.57–25.23) | 407 (329–496) | 10.48 (8.15–12.72) | 15,466 (12,552–19,241) | 319.63 (260.12–389.93) |
| 1999 | 997 (773–1,268) | 20.73 (16.21–25.68) | 435 (343–528) | 10.65 (8.43–12.79) | 16,600 (13,195–20,338) | 327.25 (261.06–398.49) |
| 2000 | 1,096 (856–1,372) | 21.84 (17.22–26.75) | 468 (369–564) | 10.99 (8.78–13.10) | 17,890 (13,929–21,811) | 338.68 (267.85–409.95) |
| 2001 | 1,205 (944–1,481) | 23.05 (18.26–28.09) | 504 (397–619) | 11.35 (9.17–13.91) | 19,270 (15,107–23,373) | 350.84 (277.36–428.33) |
| 2002 | 1,327 (1,040–1,684) | 24.40 (19.33–30.65) | 545 (440–669) | 11.79 (9.52–14.44) | 20,833 (16,581–25,841) | 365.33 (293.83–450.84) |
| 2003 | 1,454 (1,150–1,861) | 25.85 (20.82–32.83) | 588 (474–728) | 12.29 (10.00–15.27) | 22,378 (17,815–27,826) | 379.93 (304.83–469.02) |
| 2004 | 1,585 (1,250–2,032) | 27.27 (21.71–34.88) | 630 (502–783) | 12.78 (10.31–15.90) | 23,922 (19,102–30,058) | 393.33 (313.83–489.88) |
| 2005 | 1,761 (1,387–2,204) | 29.24 (23.14–37.10) | 688 (547–850) | 13.53 (10.94–16.72) | 26,151 (20,900–32,810) | 414.48 (331.39–509.91) |
| 2006 | 1,966 (1,544–2,474) | 31.52 (24.64–39.96) | 756 (600–935) | 14.42 (11.59–17.69) | 28,759 (22,935–35,806) | 439.71 (351.55–546.04) |
| 2007 | 2,187 (1,712–2,777) | 33.84 (26.68–42.86) | 825 (655–1,017) | 15.29 (12.29–18.71) | 31,475 (25,131–39,189) | 464.41 (371.21–576.19) |
| 2008 | 2,419 (1,875–3,097) | 36.42 (28.51–45.69) | 894 (705–1,119) | 16.27 (13.03–19.96) | 34,019 (26,934–43,184) | 487.89 (391.06–605.70) |
| 2009 | 2,630 (2,040–3,415) | 38.63 (30.48–48.37) | 952 (757–1,188) | 17.06 (13.69–20.85) | 36,128 (28,582–46,361) | 504.82 (404.04–626.59) |
| 2010 | 2,807 (2,215–3,720) | 40.54 (32.43–50.97) | 995 (800–1,224) | 17.72 (14.44–21.64) | 37,567 (30,008–48,802) | 515.28 (414.49–633.11) |
| 2011 | 2,928 (2,268–3,921) | 41.74 (33.32–51.99) | 1,014 (816–1,270) | 18.03 (14.89–21.84) | 38,048 (30,346–50,461) | 514.96 (416.77–632.05) |
| 2012 | 2,983 (2,280–4,017) | 42.20 (33.14–53.18) | 1,013 (818–1,281) | 18.04 (14.81–22.03) | 37,686 (29,707–50,391) | 506.25 (408.63–623.72) |
| 2013 | 2,978 (2,276–4,100) | 42.02 (32.90–52.75) | 998 (802–1,290) | 17.86 (14.67–21.96) | 36,719 (28,894–50,690) | 492.13 (400.28–619.21) |
| 2014 | 3,010 (2,331–4,223) | 42.45 (33.97–53.42) | 1,001 (799–1,317) | 17.99 (14.78–21.69) | 36,375 (28,368–51,067) | 487.20 (392.61–620.50) |
| 2015 | 2,951 (2,232–4,256) | 41.49 (32.29–52.68) | 971 (771–1,321) | 17.48 (14.20–21.29) | 34,993 (27,328–50,951) | 467.15 (371.69–615.69) |
| 2016 | 2,987 (2,321–4,308) | 41.33 (33.02–52.46) | 965 (774–1,310) | 17.13 (13.90–20.64) | 34,740 (27,414–50,690) | 457.18 (366.84–602.97) |
| 2017 | 3,080 (2,425–4,388) | 41.86 (33.02–53.23) | 977 (782–1,326) | 17.01 (13.40–20.52) | 35,151 (28,130–50,687) | 455.23 (360.86–600.77) |
| 2018 | 3,137 (2,488–4,481) | 41.96 (33.53–53.88) | 977 (784–1,331) | 16.70 (13.11–20.12) | 35,126 (28,499–51,119) | 448.98 (354.06–595.21) |
| 2019 | 3,316 (2,599–4,771) | 43.68 (34.89–56.58) | 1,014 (823–1,401) | 17.00 (13.49–20.63) | 36,406 (29,687–53,284) | 459.43 (369.50–615.63) |
| 2020 | 3,567 (2,751–5,077) | 47.28 (37.10–61.63) | 1,084 (877–1,482) | 18.26 (14.60–22.16) | 38,370 (30,962–55,547) | 487.54 (393.49–650.84) |
| 2021 | 3,861 (2,996–5,519) | 49.89 (38.55–65.44) | 1,145 (932–1,555) | 18.76 (14.96–23.06) | 41,000 (33,229–58,964) | 508.85 (405.60–677.29) |
| 2022 | 4,005 (2,993–5,706) | 50.63 (37.96–66.63) | 1,167 (927–1,615) | 18.72 (14.48–23.12) | 42,047 (32,986–61,569) | 511.30 (404.49–686.34) |
| **2023** | 4,025 (2,886–5,836) | 49.36 (36.19–66.12) | 1,149 (852–1,625) | 17.86 (13.01–22.54) | 41,855 (31,446–62,282) | 494.92 (365.40–681.99) |
| **MALE** | | | | | | |
| **1990** | 16 (8–32) | 0.42 (0.21–0.89) | 9 (5–20) | 0.29 (0.14–0.60) | 312 (153–662) | 7.36 (3.58–15.44) |
| 1991 | 17 (8–34) | 0.43 (0.21–0.90) | 10 (5–20) | 0.29 (0.14–0.59) | 326 (158–683) | 7.34 (3.51–15.23) |
| 1992 | 17 (8–37) | 0.43 (0.21–0.91) | 10 (5–21) | 0.29 (0.14–0.60) | 340 (162–702) | 7.28 (3.53–15.09) |
| 1993 | 18 (9–40) | 0.43 (0.22–0.91) | 11 (5–21) | 0.29 (0.14–0.59) | 355 (174–726) | 7.28 (3.54–14.98) |
| 1994 | 19 (9–41) | 0.43 (0.21–0.90) | 11 (5–22) | 0.29 (0.14–0.59) | 370 (180–752) | 7.25 (3.55–14.85) |
| 1995 | 21 (10–43) | 0.45 (0.22–0.89) | 12 (6–23) | 0.29 (0.14–0.59) | 396 (193–801) | 7.41 (3.70–15.09) |
| 1996 | 21 (10–42) | 0.43 (0.20–0.86) | 12 (6–24) | 0.28 (0.14–0.57) | 398 (192–821) | 7.15 (3.48–14.57) |
| 1997 | 22 (10–42) | 0.44 (0.21–0.83) | 12 (6–24) | 0.28 (0.14–0.56) | 418 (207–836) | 7.20 (3.62–14.15) |
| 1998 | 24 (12–43) | 0.45 (0.22–0.82) | 13 (7–25) | 0.29 (0.15–0.56) | 437 (226–873) | 7.25 (3.79–14.17) |
| 1999 | 25 (12–48) | 0.46 (0.23–0.87) | 13 (7–26) | 0.29 (0.16–0.55) | 462 (241–908) | 7.35 (3.92–14.21) |
| 2000 | 28 (14–52) | 0.48 (0.25–0.90) | 14 (8–27) | 0.30 (0.17–0.55) | 499 (275–946) | 7.62 (4.25–14.36) |
| 2001 | 30 (16–56) | 0.51 (0.28–0.92) | 16 (9–29) | 0.31 (0.18–0.57) | 541 (310–998) | 7.96 (4.60–14.76) |
| 2002 | 34 (19–61) | 0.55 (0.30–0.97) | 17 (10–31) | 0.33 (0.20–0.59) | 602 (360–1,062) | 8.51 (5.16–15.14) |
| 2003 | 38 (22–68) | 0.60 (0.34–1.02) | 19 (12–32) | 0.35 (0.22–0.60) | 673 (421–1,139) | 9.12 (5.65–15.34) |
| 2004 | 44 (26–77) | 0.65 (0.39–1.12) | 21 (13–35) | 0.37 (0.24–0.60) | 756 (467–1,236) | 9.79 (6.10–15.92) |
| 2005 | 50 (29–86) | 0.70 (0.42–1.20) | 24 (15–38) | 0.40 (0.25–0.64) | 845 (517–1,386) | 10.44 (6.48–16.91) |
| 2006 | 56 (31–96) | 0.76 (0.44–1.28) | 26 (16–43) | 0.42 (0.26–0.69) | 944 (578–1,578) | 11.15 (6.86–18.41) |
| 2007 | 63 (34–107) | 0.82 (0.45–1.35) | 29 (17–47) | 0.45 (0.27–0.72) | 1,043 (620–1,734) | 11.78 (7.04–19.35) |
| 2008 | 70 (36–119) | 0.86 (0.46–1.44) | 31 (18–51) | 0.46 (0.27–0.76) | 1,129 (665–1,889) | 12.17 (7.13–20.20) |
| 2009 | 76 (40–128) | 0.91 (0.48–1.51) | 33 (20–55) | 0.48 (0.29–0.79) | 1,213 (713–2,008) | 12.55 (7.51–20.68) |
| 2010 | 84 (43–140) | 0.97 (0.50–1.62) | 35 (21–59) | 0.49 (0.29–0.83) | 1,303 (775–2,168) | 13.00 (7.78–21.36) |
| 2011 | 90 (47–158) | 1.01 (0.53–1.77) | 37 (21–60) | 0.51 (0.30–0.82) | 1,358 (811–2,211) | 13.18 (7.84–21.41) |
| 2012 | 95 (52–172) | 1.04 (0.58–1.85) | 37 (22–62) | 0.51 (0.30–0.84) | 1,391 (830–2,254) | 13.19 (7.89–21.55) |
| 2013 | 98 (54–175) | 1.06 (0.59–1.89) | 38 (23–63) | 0.52 (0.31–0.86) | 1,406 (845–2,306) | 13.13 (7.86–21.69) |
| 2014 | 104 (54–189) | 1.12 (0.60–1.99) | 40 (23–65) | 0.54 (0.31–0.88) | 1,453 (853–2,388) | 13.48 (7.92–22.01) |
| 2015 | 105 (53–195) | 1.12 (0.59–2.02) | 40 (22–65) | 0.54 (0.30–0.88) | 1,442 (814–2,369) | 13.24 (7.50–21.64) |
| 2016 | 106 (55–199) | 1.13 (0.59–2.04) | 40 (23–63) | 0.54 (0.30–0.86) | 1,424 (798–2,279) | 13.05 (7.37–20.99) |
| 2017 | 109 (52–197) | 1.15 (0.58–2.02) | 40 (22–65) | 0.54 (0.29–0.87) | 1,422 (782–2,297) | 12.97 (7.22–21.06) |
| 2018 | 112 (55–194) | 1.18 (0.57–2.06) | 41 (22–67) | 0.55 (0.29–0.88) | 1,432 (786–2,347) | 12.96 (7.08–20.70) |
| 2019 | 119 (61–197) | 1.23 (0.62–2.12) | 43 (23–68) | 0.56 (0.30–0.90) | 1,492 (831–2,392) | 13.33 (7.34–21.27) |
| 2020 | 127 (66–213) | 1.33 (0.69–2.21) | 45 (25–71) | 0.61 (0.34–0.95) | 1,555 (877–2,429) | 14.02 (7.87–21.72) |
| 2021 | 131 (69–226) | 1.32 (0.70–2.26) | 46 (26–72) | 0.59 (0.33–0.92) | 1,583 (888–2,492) | 13.78 (7.72–21.45) |
| 2022 | 134 (70–229) | 1.31 (0.69–2.20) | 46 (26–75) | 0.58 (0.32–0.95) | 1,609 (887–2,576) | 13.49 (7.59–21.71) |
| **2023** | 143 (76–243) | 1.31 (0.69–2.21) | 48 (27–75) | 0.57 (0.31–0.93) | 1,706 (946–2,710) | 13.47 (7.45–21.27) |

***Abbreviations:*** *ASR, Age-Standardized Rate; DALYs, Disability-Adjusted Life-Years; GBD, Global Burden of Disease; UI, Uncertainty Interval. ASR = Age-Standardized Rate Per 100,000 Population. Female:Male Incidence Ratio In 2023 = 28.2:1. Female Cases Represent 96.6% of Total Incidence Burden.*
