# Supplementary material for: National Burden of Breast Cancer in Saudi Arabia, 1990–2023, With Forecasts to 2050: A Systematic Analysis for the Global Burden of Disease Study 2023
Source: Evidance Health Sci. Author manuscript; Available in PMC 2026 May 7. (PMC13148422; doi:10.65416/ehealthsci.2026.117757)
Supplement: Appendix — Supplementary Figure 1: Joinpoint Regression Analysis of Incidence and Mortality Trends. Supplementary Figure 2: Lee-Carter Model Mortality Forecast To 2050. Supplementary Figure 3: Bayesian Age-Period-Cohort Variance Decomposition. Supplementary Figure 4: Compression Versus Expansion of Morbidity Analysis. Table 1: Annual Time Series of Breast Cancer Burden In Saudi Arabia, 1990–2023. Supplementary Table 2: Sex-Specific Annual Time Series of Breast Cancer Burden In Saudi Arabia, 1990–2023. Supplementary Table 3: Annual Time Series of YLLs, YLDs, and Prevalence For Breast Cancer In Saudi Arabia, 1990–2023. Supplementary Table 4: Detailed Statistical Analysis and Sensitivity Assessment of Breast Cancer Trends In Saudi Arabia, 1990–2023. [file NIHMS2163534-supplement-Appendix.zip › Supplementary Table 3.docx]

**Supplementary Table 3:** Annual Time Series of YLLs, YLDs, and Prevalence For Breast Cancer In Saudi Arabia, 1990–2023.

| **Year** | **YLLs** | | **YLDs** | | **Prevalence** | | **DALYs Composition** | |
| --- | --- | --- | --- | --- | --- | --- | --- | --- |
|  | **Number (95% UI)** | **ASR (95% UI)** | **Number (95% UI)** | **ASR (95% UI)** | **Number (95% UI)** | **ASR (95% UI)** | **YLL%** | **YLD%** |
| **1990** | 8,208 (5,926–11,602) | 107.13 (75.90–146.56) | 316 (196–474) | 4.46 (2.83–6.50) | 4,524 (3,510–5,806) | 66.50 (52.30–82.40) | 96.3 | 3.7 |
| 1991 | 8,935 (6,585–12,197) | 109.95 (80.24–149.57) | 346 (210–535) | 4.58 (2.81–6.82) | 4,841 (3,839–6,165) | 66.63 (53.03–81.97) | 96.3 | 3.7 |
| 1992 | 9,773 (7,320–12,774) | 113.52 (84.38–148.09) | 377 (232–560) | 4.69 (2.98–6.88) | 5,212 (4,165–6,613) | 67.19 (54.27–81.83) | 96.3 | 3.7 |
| 1993 | 10,730 (8,219–13,817) | 117.85 (89.27–149.21) | 417 (256–613) | 4.88 (3.12–7.04) | 5,643 (4,572–7,022) | 68.19 (55.66–83.55) | 96.3 | 3.7 |
| 1994 | 11,756 (9,103–14,764) | 122.11 (94.57–152.20) | 459 (297–672) | 5.06 (3.35–7.36) | 6,120 (5,012–7,605) | 69.41 (57.19–85.17) | 96.2 | 3.8 |
| 1995 | 12,545 (9,737–15,555) | 123.17 (96.55–151.72) | 496 (329–715) | 5.15 (3.45–7.28) | 6,516 (5,350–8,111) | 69.52 (57.76–84.43) | 96.2 | 3.8 |
| 1996 | 13,370 (10,642–16,538) | 123.80 (98.27–151.75) | 528 (359–745) | 5.18 (3.59–7.23) | 6,928 (5,726–8,502) | 69.52 (57.86–84.27) | 96.2 | 3.8 |
| 1997 | 14,289 (11,564–17,656) | 126.01 (101.31–153.64) | 571 (388–818) | 5.31 (3.70–7.47) | 7,400 (6,082–8,949) | 70.29 (58.45–84.18) | 96.2 | 3.8 |
| 1998 | 15,287 (12,436–18,854) | 128.75 (104.44–155.15) | 616 (403–870) | 5.47 (3.71–7.68) | 7,945 (6,433–9,547) | 71.61 (59.81–85.57) | 96.1 | 3.9 |
| 1999 | 16,393 (13,025–19,899) | 131.98 (105.09–158.81) | 670 (445–924) | 5.67 (3.83–7.78) | 8,584 (7,008–10,250) | 73.58 (61.20–87.54) | 96.1 | 3.9 |
| 2000 | 17,657 (13,890–21,553) | 136.52 (109.28–163.35) | 732 (496–1,038) | 5.93 (4.01–8.19) | 9,377 (7,552–11,393) | 76.81 (63.04–91.75) | 96.0 | 4.0 |
| 2001 | 19,009 (15,119–22,986) | 141.83 (114.51–172.90) | 802 (531–1,147) | 6.27 (4.31–8.69) | 10,247 (8,071–12,587) | 80.89 (65.13–97.81) | 96.0 | 4.0 |
| 2002 | 20,562 (16,588–25,523) | 148.12 (120.39–182.16) | 873 (585–1,243) | 6.61 (4.51–9.15) | 11,207 (8,821–13,710) | 85.47 (68.03–103.28) | 95.9 | 4.1 |
| 2003 | 22,091 (17,727–27,570) | 154.31 (125.00–190.23) | 960 (654–1,377) | 7.04 (4.83–9.91) | 12,319 (9,625–15,397) | 90.78 (72.56–111.95) | 95.8 | 4.2 |
| 2004 | 23,626 (18,868–29,523) | 159.91 (128.08–195.92) | 1,052 (703–1,531) | 7.45 (5.10–10.83) | 13,499 (10,518–16,968) | 96.07 (76.62–117.99) | 95.7 | 4.3 |
| 2005 | 25,834 (20,680–32,082) | 168.24 (135.40–205.29) | 1,162 (772–1,675) | 7.90 (5.34–10.88) | 14,916 (11,756–18,563) | 102.02 (81.79–125.78) | 95.7 | 4.3 |
| 2006 | 28,402 (22,706–35,326) | 178.29 (143.28–219.64) | 1,301 (898–1,839) | 8.52 (5.95–11.47) | 16,615 (13,028–20,456) | 109.28 (87.30–133.17) | 95.6 | 4.4 |
| 2007 | 31,065 (24,751–38,854) | 187.89 (150.18–230.66) | 1,453 (992–2,064) | 9.15 (6.28–12.31) | 18,484 (14,644–22,902) | 117.01 (94.16–142.69) | 95.5 | 4.5 |
| 2008 | 33,549 (26,659–42,315) | 196.88 (156.37–242.75) | 1,598 (1,099–2,303) | 9.76 (6.89–13.28) | 20,464 (16,245–25,694) | 125.34 (101.83–151.69) | 95.5 | 4.5 |
| 2009 | 35,597 (28,211–45,167) | 203.02 (162.23–250.17) | 1,743 (1,150–2,529) | 10.31 (7.08–14.31) | 22,284 (17,537–28,273) | 132.44 (105.98–160.49) | 95.3 | 4.7 |
| 2010 | 37,007 (29,556–47,702) | 206.31 (167.86–251.41) | 1,862 (1,286–2,571) | 10.75 (7.76–14.73) | 23,900 (18,627–30,599) | 138.35 (109.82–167.14) | 95.2 | 4.8 |
| 2011 | 37,462 (29,712–49,415) | 205.94 (168.07–248.51) | 1,944 (1,310–2,737) | 11.04 (7.72–15.56) | 25,083 (19,827–32,634) | 142.99 (114.86–173.30) | 95.1 | 4.9 |
| 2012 | 37,089 (29,553–49,565) | 202.18 (165.39–244.79) | 1,988 (1,267–2,865) | 11.17 (7.57–15.73) | 25,815 (20,211–34,039) | 145.70 (117.79–178.83) | 94.9 | 5.1 |
| 2013 | 36,130 (28,769–49,377) | 196.30 (160.56–241.39) | 1,995 (1,248–2,935) | 11.15 (7.47–15.43) | 26,065 (20,257–35,148) | 146.41 (118.75–178.96) | 94.8 | 5.2 |
| 2014 | 35,810 (28,384–50,251) | 194.12 (157.35–242.91) | 2,018 (1,269–3,067) | 11.21 (7.24–15.51) | 26,540 (20,335–36,008) | 148.56 (119.68–181.78) | 94.7 | 5.3 |
| 2015 | 34,446 (27,268–50,189) | 185.26 (149.97–235.02) | 1,989 (1,269–3,016) | 10.97 (7.31–15.26) | 26,303 (20,447–36,818) | 146.31 (118.33–181.74) | 94.5 | 5.5 |
| 2016 | 34,157 (27,000–49,447) | 180.55 (145.76–230.28) | 2,007 (1,336–3,113) | 10.89 (7.40–15.15) | 26,820 (21,357–37,713) | 147.00 (119.84–182.78) | 94.4 | 5.6 |
| 2017 | 34,514 (27,910–49,259) | 178.84 (143.60–228.70) | 2,058 (1,355–3,148) | 10.98 (7.44–15.50) | 27,624 (22,205–38,711) | 149.19 (123.68–187.94) | 94.4 | 5.6 |
| 2018 | 34,457 (28,280–49,779) | 175.22 (140.85–223.85) | 2,102 (1,419–3,238) | 10.96 (7.62–15.33) | 28,324 (22,839–39,767) | 150.44 (123.26–191.98) | 94.3 | 5.7 |
| 2019 | 35,686 (29,697–51,901) | 177.79 (144.45–228.45) | 2,212 (1,452–3,396) | 11.25 (7.66–15.96) | 29,951 (24,392–41,504) | 155.64 (129.10–198.26) | 94.2 | 5.8 |
| 2020 | 37,626 (31,045–54,274) | 187.58 (152.26–239.85) | 2,299 (1,476–3,495) | 11.58 (7.90–16.29) | 31,133 (25,741–42,166) | 159.83 (133.12–201.00) | 94.2 | 5.8 |
| 2021 | 40,074 (33,081–57,001) | 192.88 (155.85–251.52) | 2,509 (1,588–3,845) | 12.21 (8.27–17.45) | 34,100 (27,959–46,560) | 169.42 (141.22–213.70) | 94.1 | 5.9 |
| 2022 | 41,062 (32,929–60,152) | 191.27 (152.14–247.17) | 2,594 (1,662–3,939) | 12.22 (8.17–17.91) | 35,421 (28,440–49,168) | 170.95 (139.28–215.66) | 94.1 | 5.9 |
| **2023** | 40,953 (31,021–61,107) | 182.78 (137.07–242.50) | 2,608 (1,688–4,122) | 11.89 (7.97–17.25) | 35,811 (28,656–49,712) | 167.66 (136.59–208.04) | 94.0 | 6.0 |

***Notes:*** *YLL% and YLD% represent the proportion of total DALYs attributable to premature mortality (YLLs) and disability (YLDs), respectively. DALYs = YLLs + YLDs.* ***Abbreviations:*** *ASR, Age-Standardized Rate; DALYs, Disability-Adjusted Life-Years; GBD, Global Burden of Disease; UI, Uncertainty Interval; YLDs, Years Lived With Disability; YLLs, Years of Life Lost. All Estimates Are For Both Sexes Combined. ASR = Age-Standardized Rate Per 100,000 Population.*
