# Supplementary material for: National Burden of Breast Cancer in Saudi Arabia, 1990–2023, With Forecasts to 2050: A Systematic Analysis for the Global Burden of Disease Study 2023
Source: Evidance Health Sci. Author manuscript; Available in PMC 2026 May 7. (PMC13148422; doi:10.65416/ehealthsci.2026.117757)
Supplement: Appendix — Supplementary Figure 1: Joinpoint Regression Analysis of Incidence and Mortality Trends. Supplementary Figure 2: Lee-Carter Model Mortality Forecast To 2050. Supplementary Figure 3: Bayesian Age-Period-Cohort Variance Decomposition. Supplementary Figure 4: Compression Versus Expansion of Morbidity Analysis. Table 1: Annual Time Series of Breast Cancer Burden In Saudi Arabia, 1990–2023. Supplementary Table 2: Sex-Specific Annual Time Series of Breast Cancer Burden In Saudi Arabia, 1990–2023. Supplementary Table 3: Annual Time Series of YLLs, YLDs, and Prevalence For Breast Cancer In Saudi Arabia, 1990–2023. Supplementary Table 4: Detailed Statistical Analysis and Sensitivity Assessment of Breast Cancer Trends In Saudi Arabia, 1990–2023. [file NIHMS2163534-supplement-Appendix.zip › Supplementary Table 4.docx]

**Supplementary Table 4:** Detailed Statistical Analysis and Sensitivity Assessment of Breast Cancer Trends In Saudi Arabia, 1990–2023.

| **Period-Specific Annual Percent Change (APC) with 95% CI:** | | | | | | |
| --- | --- | --- | --- | --- | --- | --- |
| **Measure** | **1990–2000** | **2000–2010** | **2010–2023** | **Overall (1990–2023)** | **P-value** | **Trend Pattern** |
| **Incidence** | +3.44 (+3.11, +3.78) | +6.55 (+6.33, +6.78) | +1.11 (+0.56, +1.66) | +3.85 (+3.49, +4.20) | <0.0001* | Acceleration→Plateau |
| **Mortality** | +1.49 (+1.16, +1.83) | +5.05 (+4.77, +5.34) | -0.14 (-0.59, +0.32) | +2.36 (+2.05, +2.68) | <0.0001* | Acceleration→Stabilization |
| **DALYs** | +2.29 (+1.99, +2.58) | +4.60 (+4.31, +4.88) | -0.73 (-1.34, -0.12) | +1.96 (+1.59, +2.34) | <0.0001* | Acceleration→Decline |
| **Prevalence** | +1.25 (+0.92, +1.59) | +6.28 (+6.08, +6.48) | +1.50 (+1.14, +1.86) | +3.50 (+3.20, +3.80) | <0.0001* | Sustained Increase |
| **YLLs** | +2.27 (+1.97, +2.57) | +4.51 (+4.23, +4.80) | -0.82 (-1.44, -0.19) | +1.88 (+1.50, +2.26) | <0.0001* | Acceleration→Decline |
| **YLDs** | +2.70 (+2.44, +2.97) | +6.37 (+6.14, +6.61) | +0.78 (+0.37, +1.18) | +3.53 (+3.18, +3.88) | <0.0001* | Sustained Increase |
| **Non-Parametric Trend Tests (Mann-Kendall and Sen's Slope Estimator):** | | | | | | |
| **Measure** | **Mann-Kendall S** | **Mann-Kendall Z** | **MK P-value** | **Sen's Slope** | **Sen's 95% CI** | **Trend** |
| **Incidence** | 519 | +7.68 | <0.0001*** | +0.458 | (-0.060, +0.928) | Increasing |
| **Mortality** | 453 | +6.70 | <0.0001*** | +0.135 | (-0.105, +0.351) | Increasing |
| **DALYs** | 367 | +5.43 | <0.0001*** | +3.006 | (-4.765, +8.839) | Increasing |
| **Prevalence** | 551 | +8.15 | <0.0001*** | +3.683 | (+0.345, +7.115) | Increasing |
| **YLLs** | 359 | +5.31 | <0.0001*** | +2.766 | (-4.702, +8.331) | Increasing |
| **YLDs** | 517 | +7.65 | <0.0001*** | +0.267 | (-0.035, +0.557) | Increasing |
| **Structural Break Analysis (Chow Test and Pettitt Test):** | | | | | | |
| **Measure** | **Chow Break Year** | **Chow F-stat** | **Chow P-value** | **Pettitt Break Year** | **Pettitt K** | **Pettitt P-value** |
| **Incidence** | 2007 | 26.51 | <0.0001*** | 2006 | 289.0 | <0.0001*** |
| **Mortality** | 2008 | 42.35 | <0.0001*** | 2006 | 289.0 | <0.0001*** |
| **DALYs** | 2007 | 75.71 | <0.0001*** | 2005 | 288.0 | <0.0001*** |
| **Prevalence** | 2006 | 25.27 | <0.0001*** | 2006 | 289.0 | <0.0001*** |
| **YLLs** | 2007 | 78.54 | <0.0001*** | 2005 | 288.0 | <0.0001*** |
| **YLDs** | 2007 | 35.66 | <0.0001*** | 2006 | 289.0 | <0.0001*** |
| **Joinpoint Regression Analysis - Trend Segmentation:** | | | | | | |
| **Measure** | **Joinpoint Year** | **Segment 1 Period** | **Segment 1 APC** | **Segment 2 Period** | **Segment 2 APC** | **Interpretation** |
| **Incidence** | 2007 | 1990–2007 | +4.60% | 2007–2023 | +1.52% | Growth deceleration |
| **Mortality** | 2007 | 1990–2007 | +2.68% | 2007–2023 | +0.35% | Near stabilization |
| **DALYs** | 2007 | 1990–2007 | +3.15% | 2007–2023 | -0.51% | Trend reversal |
| **Prevalence** | 2005 | 1990–2005 | +2.72% | 2005–2023 | +2.38% | Consistent growth |
| **YLLs** | 2007 | 1990–2007 | +3.10% | 2007–2023 | -0.61% | Trend reversal |
| **YLDs** | 2007 | 1990–2007 | +4.11% | 2007–2023 | +1.24% | Growth deceleration |
| **Model Selection by Multiple Criteria (BIC Weights):** | | | | | | |
| **Measure** | **Linear (%)** | **Quadratic (%)** | **Cubic (%)** | **Log-linear (%)** | **Best Model** | **Confidence** |
| **Incidence** | 0.0 | 0.0 | 99.9 | 0.0 | Cubic | 99.9% |
| **Mortality** | 0.0 | 0.0 | 100.0 | 0.0 | Cubic | 100.0% |
| **DALYs** | 0.0 | 1.7 | 98.3 | 0.0 | Cubic | 98.3% |
| **Prevalence** | 0.0 | 0.0 | 100.0 | 0.0 | Cubic | 100.0% |
| **YLLs** | 0.0 | 2.4 | 97.6 | 0.0 | Cubic | 97.6% |
| **YLDs** | 0.0 | 0.0 | 100.0 | 0.0 | Cubic | 100.0% |
| **Monte Carlo Uncertainty Propagation (n=10,000 simulations):** | | | | | | |
| **Measure** | **MC Mean APC** | **MC Median APC** | **MC 95% CI** | **MC Standard Deviation** | **Robustness** | |
| **Incidence** | +3.853% | +3.853% | (+3.356, +4.343) | 0.251 | Confirmed | |
| **Mortality** | +2.370% | +2.367% | (+1.939, +2.820) | 0.223 | Confirmed | |
| **DALYs** | +1.964% | +1.963% | (+1.509, +2.433) | 0.236 | Confirmed | |
| **Prevalence** | +3.495% | +3.496% | (+3.102, +3.889) | 0.199 | Confirmed | |
| **YLLs** | +1.884% | +1.883% | (+1.421, +2.354) | 0.238 | Confirmed | |
| **YLDs** | +3.542% | +3.537% | (+2.820, +4.281) | 0.377 | Confirmed | |
| **Effect Size Analysis (Cohen's d) Between Periods:** | | | | | | |
| **Measure** | **1990-2000 vs 2000-2010** | **Effect** | **2000-2010 vs 2010-2023** | **Effect** | **Pre-break vs Post-break** | **Effect** |
| **Incidence** | d = +2.91 | Large | d = +2.74 | Large | d = +4.73 | Large |
| **Mortality** | d = +2.65 | Large | d = +2.14 | Large | d = +5.19 | Large |
| **DALYs** | d = +3.01 | Large | d = +0.95 | Large | d = +3.59 | Large |
| **Prevalence** | d = +2.71 | Large | d = +3.05 | Large | d = +4.93 | Large |
| **YLLs** | d = +3.02 | Large | d = +0.83 | Large | d = +3.44 | Large |
| **YLDs** | d = +2.86 | Large | d = +2.72 | Large | d = +5.01 | Large |
| **Cross-Validation and Forecast Accuracy Metrics:** | | | | | | |
| **Measure** | **LOOCV MAE** | **LOOCV MAPE (%)** | **Out-of-Sample MAE** | **Out-of-Sample MAPE (%)** | **Theil's U** | **Direction Accuracy** |
| **Incidence** | 0.903 | 7.03 | 1.301 | 6.74 | 0.660 | 75.0% |
| **Mortality** | 0.439 | 6.67 | 0.970 | 12.53 | 1.935 | 50.0% |
| **DALYs** | 14.900 | 8.32 | 33.979 | 17.21 | 2.568 | 50.0% |
| **Prevalence** | 7.384 | 7.80 | 4.430 | 2.72 | 0.346 | 75.0% |
| **YLLs** | 14.442 | 8.50 | 32.882 | 17.71 | 2.668 | 50.0% |
| **YLDs** | 0.600 | 7.29 | 1.096 | 9.30 | 1.197 | 75.0% |
| **Trend Velocity and Acceleration Analysis:** | | | | | | |
| **Measure** | **Year** | **ASR Value** | **Velocity** | **Acceleration** | **Phase** | |
| **Incidence** | 1990 | 6.47 | -0.004 | +0.082 | Decelerating decline | |
|  | 2000 | 9.30 | +0.550 | +0.029 | Accelerating growth | |
|  | 2010 | 17.17 | +0.567 | -0.025 | Decelerating growth | |
|  | 2023 | 19.58 | -0.213 | -0.095 | Accelerating decline | |
| **Mortality** | 1990 | 4.12 | -0.073 | +0.041 | Decelerating decline | |
|  | 2010 | 7.79 | +0.185 | -0.015 | Decelerating growth | |
|  | 2023 | 7.57 | -0.244 | -0.051 | Accelerating decline | |
| **DALYs** | 1990 | 111.58 | +1.949 | +0.599 | Accelerating growth | |
|  | 2010 | 217.07 | +3.343 | -0.460 | Decelerating growth | |
|  | 2023 | 194.66 | -7.106 | -1.148 | Accelerating decline | |
| **Variance Decomposition Analysis:** | | | | | | |
| **Measure** | **Total Variance** | **Trend Component (%)** | **Cyclical Component (%)** | **Irregular Component (%)** | **Dominant Pattern** | |
| **Incidence** | 22.72 | 96.0% | 3.6% | 0.4% | Trend-dominated | |
| **Mortality** | 2.20 | 89.5% | 9.5% | 1.1% | Trend-dominated | |
| **DALYs** | 1247.85 | 79.1% | 19.7% | 1.2% | Trend-dominated | |
| **Prevalence** | 1432.14 | 96.2% | 3.5% | 0.3% | Trend-dominated | |
| **YLLs** | 1068.11 | 77.2% | 21.5% | 1.3% | Trend-dominated | |
| **YLDs** | 7.82 | 95.0% | 4.6% | 0.4% | Trend-dominated | |
| **Statistical Power Analysis:** | | | | | | |
| **Measure** | **Effect Size** | **Achieved Power (%)** | **N for 80% Power** | **N for 90% Power** | **Power Status** | |
| **Incidence** | 0.976 | 100.0% | 11 | 14 | Adequately powered | |
| **Mortality** | 0.936 | 100.0% | 12 | 15 | Adequately powered | |
| **DALYs** | 0.874 | 99.9% | 13 | 16 | Adequately powered | |
| **Prevalence** | 0.976 | 100.0% | 11 | 14 | Adequately powered | |
| **YLLs** | 0.862 | 99.8% | 13 | 17 | Adequately powered | |
| **YLDs** | 0.970 | 100.0% | 11 | 14 | Adequately powered | |
| **Regression Diagnostics and Residual Analysis:** | | | | | | |
| **Measure** | **Durbin-Watson** | **Autocorrelation** | **Shapiro-Wilk P** | **Normality** | **ACF(1)** | **Spearman ρ** |
| **Incidence** | 0.176 | Positive | 0.050 | Borderline | 0.911 | 0.979 |
| **Mortality** | 0.143 | Positive | 0.043 | Non-normal | 0.928 | 0.918 |
| **DALYs** | 0.094 | Positive | 0.003 | Non-normal | 0.952 | 0.801 |
| **Prevalence** | 0.142 | Positive | 0.106 | Normal | 0.929 | 0.998 |
| **YLLs** | 0.093 | Positive | 0.003 | Non-normal | 0.953 | 0.791 |
| **YLDs** | 0.129 | Positive | 0.019 | Non-normal | 0.936 | 0.978 |
| **Heterogeneity Tests Across Measures:** | | | | | | |
| **Test** | | **Statistic** | **DF** | **P-value** | **Result** | |
| Cochran's Q (APC heterogeneity) | | 128.04 | 5 | <0.0001 | I²=96.1% (High heterogeneity) | |
| Z-test (1990-2007 vs 2007-2023) | | 10.39 | — | <0.0001 | Significant trend change | |
| F-test (period slopes equality) | | 42.35 | 30 | <0.0001 | Slopes differ significantly | |
| **Change Summary (1990→2023):** | | | | | | |
| **Measure** | **1990 ASR** | **2023 ASR** | **Absolute Change** | **Relative Change (%)** | **CAGR (%)** | **CI Overlap** |
| **Incidence** | 6.47 | 19.58 | +13.11 | +202.7% | +3.41% | No (significant) |
| **Mortality** | 4.12 | 7.57 | +3.46 | +84.0% | +1.86% | No (significant) |
| **DALYs** | 111.58 | 194.66 | +83.08 | +74.5% | +1.70% | Minimal (5%) |
| **Prevalence** | 66.50 | 167.66 | +101.16 | +152.1% | +2.84% | No (significant) |
| **YLLs** | 107.13 | 182.78 | +75.65 | +70.6% | +1.63% | Minimal (8%) |
| **YLDs** | 4.46 | 11.89 | +7.43 | +166.8% | +3.02% | No (significant) |

***Notes:*** **P-value<0.05, **P-value<0.01, ***P-value<0.001. APC calculated via log-linear regression. Mann-Kendall: non-parametric monotonic trend test. Sen's slope: robust non-parametric trend magnitude. Chow test: structural break detection. Pettitt test: non-parametric change point. BIC weights: posterior model probabilities. Monte Carlo: 10,000 simulations including GBD uncertainty intervals. Cohen's d: standardized effect size (small<0.2, medium<0.5, large≥0.8). Theil's U<1 indicates model outperforms naïve forecast. Velocity: first derivative of fitted polynomial; Acceleration: second derivative.* ***Abbreviations:*** *ACF, Autocorrelation Function; AIC, Akaike Information Criterion; APC, Annual Percent Change; ASR, Age-Standardized Rate Per 100,000; BIC, Bayesian Information Criterion; CAGR, Compound Annual Growth Rate; CI, Confidence Interval; DALYs, Disability-Adjusted Life-Years; DF, Degrees of Freedom; GBD, Global Burden of Disease; LOOCV, Leave-One-Out Cross-Validation; MAE, Mean Absolute Error; MAPE, Mean Absolute Percentage Error; MC, Monte Carlo; MK, Mann-Kendall; RMSE, Root Mean Squared Error; ρ, Spearman Correlation Coefficient; UI, Uncertainty Interval; YLDs, Years Lived With Disability; YLLs, Years of Life Lost.*
