# Supplementary material for: National Burden of Breast Cancer in Saudi Arabia, 1990–2023, With Forecasts to 2050: A Systematic Analysis for the Global Burden of Disease Study 2023
Source: Evidance Health Sci. Author manuscript; Available in PMC 2026 May 7. (PMC13148422; doi:10.65416/ehealthsci.2026.117757)
Supplement: Appendix — Supplementary Figure 1: Joinpoint Regression Analysis of Incidence and Mortality Trends. Supplementary Figure 2: Lee-Carter Model Mortality Forecast To 2050. Supplementary Figure 3: Bayesian Age-Period-Cohort Variance Decomposition. Supplementary Figure 4: Compression Versus Expansion of Morbidity Analysis. Table 1: Annual Time Series of Breast Cancer Burden In Saudi Arabia, 1990–2023. Supplementary Table 2: Sex-Specific Annual Time Series of Breast Cancer Burden In Saudi Arabia, 1990–2023. Supplementary Table 3: Annual Time Series of YLLs, YLDs, and Prevalence For Breast Cancer In Saudi Arabia, 1990–2023. Supplementary Table 4: Detailed Statistical Analysis and Sensitivity Assessment of Breast Cancer Trends In Saudi Arabia, 1990–2023. [file NIHMS2163534-supplement-Appendix.zip › Supplementary Table 5.docx]

**Supplementary Table 5:** Age-Specific Burden of Breast Cancer in Saudi Arabia: Comprehensive Analysis by Age Group, 1990–2023

| **Incidence by Age Group – Number of New Cases:** | | | | | | | | | |
| --- | --- | --- | --- | --- | --- | --- | --- | --- | --- |
| **Age Group** | **1990 Cases** | **95% UI** | **2023 Cases** | **95% UI** | **% of Total (2023)** | **Absolute Change** | **Relative Change (%)** | **Fold Change** | **Trend** |
| **15-19** | 5 | (2–9) | 20 | (12–30) | 0.5% | +15 | +282% | 3.8× | ↑↑ |
| **20-24** | 6 | (3–12) | 34 | (19–50) | 0.8% | +27 | +435% | 5.4× | ↑↑↑ |
| **25-29** | 15 | (8–26) | 110 | (68–163) | 2.6% | +95 | +644% | 7.4× | ↑↑↑ |
| **30-34** | 28 | (15–48) | 265 | (165–431) | 6.4% | +237 | +836% | 9.4× | ↑↑↑↑ |
| **35-39** | 38 | (21–66) | 415 | (247–723) | 10.0% | +377 | +993% | 10.9× | ↑↑↑↑ |
| **40-44** | 54 | (32–92) | 577 | (363–1,028) | 13.8% | +523 | +970% | 10.7× | ↑↑↑↑ |
| **45-49** | 48 | (29–81) | 564 | (363–915) | 13.5% | +516 | +1,067% | 11.7× | ↑↑↑↑ |
| **50-54** | 54 | (32–90) | 504 | (333–823) | 12.1% | +450 | +830% | 9.3× | ↑↑↑↑ |
| **55-59** | 41 | (24–68) | 433 | (281–619) | 10.4% | +393 | +967% | 10.7× | ↑↑↑↑ |
| **60-64** | 56 | (33–90) | 464 | (305–655) | 11.1% | +409 | +734% | 8.3× | ↑↑↑ |
| **65-69** | 34 | (20–58) | 301 | (203–436) | 7.2% | +267 | +775% | 8.7× | ↑↑↑ |
| **70-74** | 35 | (20–58) | 198 | (133–288) | 4.8% | +164 | +474% | 5.7× | ↑↑↑ |
| **75-79** | 20 | (11–35) | 146 | (92–209) | 3.5% | +126 | +641% | 7.4× | ↑↑↑ |
| **80-84** | 15 | (8–27) | 99 | (56–143) | 2.4% | +84 | +560% | 6.6× | ↑↑↑ |
| **85-89** | 4 | (2–8) | 29 | (18–44) | 0.7% | +25 | +597% | 7.0× | ↑↑↑ |
| **90-94** | 1 | (0–1) | 6 | (4–9) | 0.1% | +6 | +978% | 10.8× | ↑↑↑↑ |
| **95+** | 0.1 | (0–0.2) | 1 | (1–2) | 0.0% | +1 | +1,975% | 20.7× | ↑↑↑↑ |
| **TOTAL** | **454** | **(308–649)** | **4,168** | **(2,962–6,079)** | **100.0%** | **+3,714** | **+819%** | **9.2×** | **↑↑↑↑** |
| **Deaths by Age Group – Number of Deaths:** | | | | | | | | | |
| **Age Group** | **1990 Deaths** | **95% UI** | **2023 Deaths** | **95% UI** | **% of Total (2023)** | **Absolute Change** | **Relative Change (%)** | **Fold Change** | **MIR (2023)** |
| **15-19** | 2 | (1–3) | 3 | (2–4) | 0.2% | +1 | +65% | 1.6× | 0.13 |
| **20-24** | 2 | (1–3) | 4 | (3–6) | 0.4% | +2 | +131% | 2.3× | 0.13 |
| **25-29** | 5 | (2–8) | 16 | (10–22) | 1.3% | +11 | +245% | 3.4× | 0.15 |
| **30-34** | 11 | (6–18) | 52 | (34–84) | 4.3% | +41 | +380% | 4.8× | 0.20 |
| **35-39** | 16 | (9–27) | 88 | (55–150) | 7.3% | +72 | +458% | 5.6× | 0.21 |
| **40-44** | 22 | (13–38) | 119 | (80–207) | 9.9% | +96 | +431% | 5.3× | 0.21 |
| **45-49** | 21 | (12–35) | 125 | (85–198) | 10.5% | +104 | +490% | 5.9× | 0.22 |
| **50-54** | 27 | (16–45) | 136 | (90–223) | 11.4% | +109 | +398% | 5.0× | 0.27 |
| **55-59** | 23 | (13–38) | 136 | (90–189) | 11.4% | +113 | +498% | 6.0× | 0.31 |
| **60-64** | 32 | (19–52) | 150 | (100–211) | 12.5% | +118 | +363% | 4.6× | 0.32 |
| **65-69** | 22 | (12–36) | 108 | (74–151) | 9.0% | +87 | +400% | 5.0× | 0.36 |
| **70-74** | 25 | (14–41) | 87 | (60–125) | 7.3% | +63 | +254% | 3.5× | 0.44 |
| **75-79** | 16 | (9–28) | 77 | (49–110) | 6.4% | +61 | +381% | 4.8× | 0.53 |
| **80-84** | 14 | (7–26) | 66 | (39–96) | 5.5% | +51 | +357% | 4.6× | 0.66 |
| **85-89** | 4 | (2–9) | 22 | (13–32) | 1.8% | +17 | +390% | 4.9× | 0.74 |
| **90-94** | 1 | (0–2) | 7 | (4–11) | 0.6% | +7 | +810% | 9.1× | 1.21 |
| **95+** | 0.1 | (0–0.2) | 2 | (1–3) | 0.1% | +2 | +1,828% | 19.3× | 1.63 |
| **TOTAL** | **242** | **(169–346)** | **1,197** | **(879–1,700)** | **100.0%** | **+955** | **+393%** | **4.9×** | **0.29** |
| **DALYs by Age Group – Disability-Adjusted Life Years:** | | | | | | | | | |
| **Age Group** | **1990 DALYs** | **95% UI** | **2023 DALYs** | **95% UI** | **% of Total (2023)** | **Absolute Change** | **Relative Change (%)** | **Fold Change** | **YLL% of DALYs** |
| **15-19** | 115 | (66–187) | 196 | (120–297) | 0.4% | +81 | +71% | 1.7× | 93.6% |
| **20-24** | 128 | (74–210) | 307 | (191–442) | 0.7% | +179 | +140% | 2.4× | 92.7% |
| **25-29** | 302 | (178–485) | 1,075 | (692–1,505) | 2.5% | +774 | +257% | 3.6× | 92.7% |
| **30-34** | 645 | (387–1,057) | 3,178 | (2,075–5,084) | 7.3% | +2,533 | +393% | 4.9× | 94.2% |
| **35-39** | 856 | (516–1,408) | 4,895 | (3,093–8,308) | 11.2% | +4,038 | +472% | 5.7× | 94.4% |
| **40-44** | 1,105 | (676–1,800) | 6,034 | (4,120–10,496) | 13.9% | +4,929 | +446% | 5.5× | 94.1% |
| **45-49** | 946 | (574–1,536) | 5,717 | (3,919–9,118) | 13.1% | +4,771 | +504% | 6.0× | 94.1% |
| **50-54** | 1,081 | (657–1,748) | 5,515 | (3,732–8,909) | 12.7% | +4,434 | +410% | 5.1× | 94.3% |
| **55-59** | 792 | (473–1,294) | 4,832 | (3,204–6,725) | 11.1% | +4,040 | +510% | 6.1× | 94.3% |
| **60-64** | 972 | (585–1,560) | 4,616 | (3,108–6,504) | 10.6% | +3,644 | +375% | 4.7× | 93.7% |
| **65-69** | 551 | (326–903) | 2,819 | (1,921–3,960) | 6.5% | +2,268 | +412% | 5.1× | 93.4% |
| **70-74** | 514 | (304–845) | 1,867 | (1,289–2,666) | 4.3% | +1,353 | +263% | 3.6× | 93.5% |
| **75-79** | 272 | (156–461) | 1,325 | (864–1,836) | 3.0% | +1,052 | +387% | 4.9× | 93.0% |
| **80-84** | 191 | (104–337) | 870 | (522–1,274) | 2.0% | +679 | +357% | 4.6× | 94.7% |
| **85-89** | 47 | (25–88) | 233 | (145–342) | 0.5% | +186 | +397% | 5.0× | 93.2% |
| **90-94** | 7 | (4–15) | 68 | (40–100) | 0.2% | +60 | +810% | 9.1× | 95.0% |
| **95+** | 1 | (0–2) | 15 | (9–23) | 0.0% | +14 | +1,827% | 19.3× | 95.5% |
| **TOTAL** | **8,524** | **(5,997–12,402)** | **43,561** | **(32,392–64,992)** | **100.0%** | **+35,037** | **+411%** | **5.1×** | **94.0%** |
| **Prevalence by Age Group – Number of Prevalent Cases:** | | | | | | | | | |
| **Age Group** | **1990 Prevalent** | **95% UI** | **2023 Prevalent** | **95% UI** | **% of Total (2023)** | **Absolute Change** | **Relative Change (%)** | **Fold Change** | **Disease Duration*** |
| **15-19** | 39 | (28–53) | 162 | (128–201) | 0.5% | +123 | +317% | 4.2× | 8.1 |
| **20-24** | 50 | (36–68) | 272 | (217–339) | 0.8% | +222 | +443% | 5.4× | 8.1 |
| **25-29** | 129 | (94–173) | 950 | (763–1,171) | 2.7% | +821 | +637% | 7.4× | 8.7 |
| **30-34** | 245 | (182–325) | 2,217 | (1,783–2,727) | 6.2% | +1,972 | +805% | 9.1× | 8.4 |
| **35-39** | 343 | (257–452) | 3,419 | (2,716–4,327) | 9.5% | +3,076 | +897% | 10.0× | 8.2 |
| **40-44** | 479 | (362–625) | 4,734 | (3,818–5,847) | 13.2% | +4,256 | +889% | 9.9× | 8.2 |
| **45-49** | 470 | (355–614) | 4,583 | (3,726–5,654) | 12.8% | +4,113 | +876% | 9.8× | 8.1 |
| **50-54** | 532 | (402–693) | 4,393 | (3,570–5,400) | 12.3% | +3,861 | +726% | 8.3× | 8.7 |
| **55-59** | 453 | (339–595) | 4,029 | (3,261–4,961) | 11.3% | +3,576 | +789% | 8.9× | 9.3 |
| **60-64** | 579 | (436–758) | 4,280 | (3,474–5,265) | 12.0% | +3,701 | +639% | 7.4× | 9.2 |
| **65-69** | 413 | (305–549) | 2,776 | (2,263–3,379) | 7.8% | +2,363 | +572% | 6.7× | 9.2 |
| **70-74** | 375 | (276–502) | 1,778 | (1,434–2,193) | 5.0% | +1,403 | +374% | 4.7× | 9.0 |
| **75-79** | 240 | (173–327) | 1,326 | (1,045–1,657) | 3.7% | +1,086 | +452% | 5.5× | 9.1 |
| **80-84** | 135 | (94–190) | 610 | (469–784) | 1.7% | +476 | +354% | 4.5× | 6.1 |
| **85-89** | 37 | (25–53) | 209 | (158–272) | 0.6% | +172 | +472% | 5.7× | 7.1 |
| **90-94** | 6 | (4–10) | 59 | (44–79) | 0.2% | +53 | +822% | 9.2× | 9.6 |
| **95+** | 1 | (0–1) | 14 | (10–20) | 0.0% | +14 | +2,023% | 21.2× | 14.3 |
| **TOTAL** | **4,524** | **(3,510–5,806)** | **35,811** | **(28,656–49,712)** | **100.0%** | **+31,287** | **+692%** | **7.9×** | **8.6** |
| **Age Distribution Summary – 2023 Burden Proportions:** | | | | | | | | | |
| **Age Category** | **Incidence (%)** | **Deaths (%)** | **DALYs (%)** | **Prevalence (%)** | **YLLs (%)** | **YLDs (%)** | **Mean MIR** | **Peak Age Group** | **Interpretation** |
| **<40 years** | 20.3% | 13.6% | 22.2% | 19.6% | 21.6% | 31.3% | 0.17 | 35-39 | Younger onset burden |
| **40-49 years** | 27.4% | 20.4% | 27.0% | 26.0% | 26.7% | 30.5% | 0.21 | 40-44 | Peak incidence ages |
| **50-64 years** | 33.6% | 35.3% | 34.3% | 35.5% | 34.1% | 38.7% | 0.30 | 60-64 | Highest mortality proportion |
| **≥65 years** | 18.7% | 30.7% | 16.5% | 18.9% | 17.6% | 20.6% | 0.51 | 65-69 | Higher case fatality |
| **<50 years** | 47.6% | 33.9% | 49.1% | 45.6% | 48.3% | 61.8% | 0.19 | 40-44 | Half of burden |
| **≥50 years** | 52.4% | 66.1% | 50.9% | 54.4% | 51.7% | 59.3% | 0.36 | 60-64 | Majority of deaths |
| **Key Age-Specific Epidemiological Indicators (2023):** | | | | | | | | | |
| **Indicator** | | **Value** | **Age Group** | **Clinical Significance** | | | | | |
| Peak incidence age | | 577 cases (13.8%) | 40-44 years | Screening target: women aged 40-50 years | | | | | |
| Peak mortality age | | 150 deaths (12.5%) | 60-64 years | Treatment intensification needed for older patients | | | | | |
| Peak DALYs age | | 6,034 DALYs (13.9%) | 40-44 years | Greatest overall burden in early middle age | | | | | |
| Peak prevalence age | | 4,734 cases (13.2%) | 40-44 years | Survivorship care priorities | | | | | |
| Lowest MIR (<50) | | 0.13 | 15-24 years | Better prognosis in younger patients | | | | | |
| Highest MIR | | 1.63 | 95+ years | Near-universal fatality in very elderly | | | | | |
| MIR crossover (>0.5) | | 0.53 | 75-79 years | Age threshold for worse outcomes | | | | | |
| YLD proportion peak | | 7.3% | 20-24 years | Higher disability proportion in young survivors | | | | | |
| Fastest growth (fold) | | 20.7× | 95+ years | Population aging driving elderly burden | | | | | |
| Largest absolute increase | | +4,929 DALYs | 40-44 years | Greatest burden growth in middle age | | | | | |

***Abbreviations:*** *DALYs, disability-adjusted life-years; MIR, mortality-to-incidence ratio; UI, uncertainty interval; YLDs, years lived with disability; YLLs, years of life lost. Both sexes combined. 95% UI = 95% uncertainty interval. MIR = mortality-to-incidence ratio. *Disease duration approximated as prevalence/incidence ratio (years). Trend arrows indicate magnitude of relative change: ↑ (<200%), ↑↑ (200-500%), ↑↑↑ (500-1000%), ↑↑↑↑ (>1000%). Age groups with <0.1 cases in 1990 (10-14 years, <1 year, 1-4 years, 5-9 years) excluded due to negligible burden.*
