# Supplementary material for: National Burden of Breast Cancer in Saudi Arabia, 1990–2023, With Forecasts to 2050: A Systematic Analysis for the Global Burden of Disease Study 2023
Source: Evidance Health Sci. Author manuscript; Available in PMC 2026 May 7. (PMC13148422; doi:10.65416/ehealthsci.2026.117757)
Supplement: Appendix — Supplementary Figure 1: Joinpoint Regression Analysis of Incidence and Mortality Trends. Supplementary Figure 2: Lee-Carter Model Mortality Forecast To 2050. Supplementary Figure 3: Bayesian Age-Period-Cohort Variance Decomposition. Supplementary Figure 4: Compression Versus Expansion of Morbidity Analysis. Table 1: Annual Time Series of Breast Cancer Burden In Saudi Arabia, 1990–2023. Supplementary Table 2: Sex-Specific Annual Time Series of Breast Cancer Burden In Saudi Arabia, 1990–2023. Supplementary Table 3: Annual Time Series of YLLs, YLDs, and Prevalence For Breast Cancer In Saudi Arabia, 1990–2023. Supplementary Table 4: Detailed Statistical Analysis and Sensitivity Assessment of Breast Cancer Trends In Saudi Arabia, 1990–2023. [file NIHMS2163534-supplement-Appendix.zip › Supplementary Table 6.docx]

**Supplementary Table 6:** Age-Specific Rate Analysis with Log-Polynomial Smoothing for Female Breast Cancer, Saudi Arabia, 2023.

| **Age Group** | **Incidence Rate** | **95% UI** | **Mortality Rate** | **95% UI** |
| --- | --- | --- | --- | --- |
| **Observed Rates (per 100,000), Saudi Arabia, Females, 2023:** | | | | |
| 15–19 years | 1.65 | 0.96–2.57 | 0.20 | 0.12–0.32 |
| 20–24 years | 3.03 | 1.72–4.55 | 0.37 | 0.22–0.55 |
| 25–29 years | 8.56 | 5.18–13.00 | 1.23 | 0.77–1.76 |
| 30–34 years | 20.50 | 12.52–33.58 | 3.98 | 2.54–6.54 |
| 35–39 years | 35.77 | 21.22–62.87 | 7.50 | 4.61–13.01 |
| 40–44 years | 65.28 | 40.96–117.58 | 13.29 | 8.86–23.69 |
| 45–49 years | 89.87 | 57.32–147.33 | 19.75 | 13.12–31.65 |
| 50–54 years | 103.28 | 67.46–170.72 | 27.69 | 17.99–46.05 |
| 55–59 years | 116.79 | 75.62–168.99 | 36.59 | 23.89–51.83 |
| 60–64 years | 170.61 | 112.28–245.32 | 54.94 | 36.20–78.86 |
| 65–69 years | 176.31 | 118.19–258.17 | 63.26 | 42.44–89.77 |
| 70–74 years | 190.42 | 123.80–278.60 | 83.79 | 55.47–122.55 |
| 75–79 years | 206.24 | 126.28–296.87 | 109.22 | 66.12–157.53 |
| 80–84 years | 357.62 | 200.24–519.67 | 237.42 | 137.31–348.14 |
| 85–89 years | 286.64 | 171.46–437.28 | 215.23 | 128.19–327.10 |
| 90–94 years | 158.34 | 91.93–242.50 | 192.19 | 109.25–294.41 |
| 95+ years | 102.12 | 56.99–167.19 | 166.69 | 92.93–272.81 |
| **Age (years)** | **Observed** | **Smoothed** | **Residual** | **% Difference** |
| **Log-Polynomial Smoothed Incidence Rates (per 100,000):** | | | | |
| 17 (15–19) | 1.65 | 1.29 | +0.36 | +21.7% |
| 22 (20–24) | 3.03 | 4.10 | -1.07 | −35.2% |
| 27 (25–29) | 8.56 | 10.03 | -1.47 | −17.2% |
| 32 (30–34) | 20.50 | 20.09 | +0.41 | +2.0% |
| 37 (35–39) | 35.77 | 34.58 | +1.19 | +3.3% |
| 42 (40–44) | 65.28 | 53.25 | +12.03 | +18.4% |
| 47 (45–49) | 89.87 | 75.73 | +14.14 | +15.7% |
| 52 (50–54) | 103.28 | 101.87 | +1.41 | +1.4% |
| 57 (55–59) | 116.79 | 131.67 | -14.88 | −12.7% |
| 62 (60–64) | 170.61 | 164.75 | +5.86 | +3.4% |
| 67 (65–69) | 176.31 | 199.45 | -23.14 | −13.1% |
| 72 (70–74) | 190.42 | 231.61 | -41.19 | −21.6% |
| 77 (75–79) | 206.24 | 253.69 | -47.45 | −23.0% |
| 82 (80–84) | 357.62 | 255.65 | +101.97 | +28.5% |
| 87 (85–89) | 286.64 | 229.34 | +57.30 | +20.0% |
| 92 (90–94) | 158.34 | 175.76 | -17.43 | −11.0% |
| 97 (95+) | 102.12 | 109.55 | -7.43 | −7.3% |
| **Model Parameter** | **Incidence** | **Mortality** | **Unit** | **Interpretation** |
| **Smoothing Model Specifications:** | | | | |
| Model type | Log-polynomial | Log-polynomial | — | Log-transformed polynomial regression |
| Polynomial degree | 4 | 4 | — | Quartic polynomial on log scale |
| Number of parameters | 5 | 5 | — | Coefficients fitted |
| Transformation | log(rate + 0.01) | log(rate + 0.01) | — | Ensures positive fitted values |
| **Model Fit Statistics:** | | | | |
| R-squared | 0.8843 | 0.9310 | — | Variance explained |
| Adjusted R-squared | 0.8316 | 0.8996 | — | Penalized for model complexity |
| RMSE | 33.55 | 20.81 | per 100,000 | Root mean square error |
| MAE | 20.51 | 10.67 | per 100,000 | Mean absolute error |
| Residual SS | 19130.84 | 7360.08 | — | Sum of squared residuals |
| **Characteristic** | **Incidence** | **Mortality** | **Unit** | **Notes** |
| **Curve Characteristics:** | | | | |
| Peak age | 80.0 | 91.5 | years | Age at maximum rate |
| Peak rate | 257.9 | 199.2 | per 100,000 | Maximum smoothed rate |
| Rate at age 25 | 7.2 | 1.1 | per 100,000 | Young adult |
| Rate at age 40 | 45.3 | 9.3 | per 100,000 | Pre-screening |
| Rate at age 50 | 91.0 | 23.1 | per 100,000 | Screening age |
| Rate at age 65 | 185.6 | 65.9 | per 100,000 | Post-menopausal |
| Rate at age 80 | 257.9 | 150.6 | per 100,000 | Elderly |
| **Inflection Points (age, years):** | | | | |
| First inflection | 65 | 79 | years | Rate of increase slows |
| Second inflection | 95 | — | years | Late decline phase |
| **Age (years)** | **dRate/dAge** | **Trend** | **Phase** | **Clinical Relevance** |
| **First Derivative Analysis (Incidence):** | | | | |
| 20 | +0.60 | Slow increase | Pre-onset | Low baseline |
| 25 | +1.25 | Slow increase | Early onset | Rising risk |
| 30 | +2.10 | Moderate increase | Young adult | Increasing |
| 35 | +2.99 | Moderate increase | Pre-menopausal | Accelerating |
| 40 | +3.82 | Moderate increase | Screening start | Rapid rise |
| 45 | +4.57 | Moderate increase | Peri-menopausal | Peak acceleration |
| 50 | +5.30 | Rapid increase | Menopausal | Highest acceleration |
| 55 | +6.03 | Rapid increase | Post-menopausal | Sustained increase |
| 60 | +6.68 | Rapid increase | Elderly onset | High acceleration |
| 65 | +6.97 | Rapid increase | Late onset | Maximum derivative |
| 70 | +6.38 | Moderate increase | Plateau phase | Rate slowing |
| 75 | +4.20 | Moderate increase | Elderly | Deceleration |
| 80 | −0.05 | Plateau | Peak phase | Rate stabilization |
| 85 | −5.87 | Rapid decrease | Decline phase | Post-peak drop |
| 90 | −11.28 | Rapid decrease | Late decline | Survivorship bias |
| **Age Group** | **MIR** | **Category** | **Survival Proxy** | **Interpretation** |
| **Mortality-to-Incidence Ratio by Age Group:** | | | | |
| 15–19 years | 0.124 | Very low | High survival | Excellent prognosis |
| 20–24 years | 0.123 | Very low | High survival | Excellent prognosis |
| 25–29 years | 0.143 | Very low | High survival | Excellent prognosis |
| 30–34 years | 0.194 | Low | High survival | Good prognosis |
| 35–39 years | 0.210 | Low | High survival | Good prognosis |
| 40–44 years | 0.204 | Low | High survival | Good prognosis |
| 45–49 years | 0.220 | Low | High survival | Good prognosis |
| 50–54 years | 0.268 | Moderate | Moderate survival | Fair prognosis |
| 55–59 years | 0.313 | Moderate | Moderate survival | Fair prognosis |
| 60–64 years | 0.322 | Moderate | Moderate survival | Fair prognosis |
| 65–69 years | 0.359 | Moderate | Moderate survival | Fair prognosis |
| 70–74 years | 0.440 | High | Lower survival | Guarded prognosis |
| 75–79 years | 0.530 | High | Lower survival | Guarded prognosis |
| 80–84 years | 0.664 | Very high | Low survival | Poor prognosis |
| 85–89 years | 0.751 | Very high | Low survival | Poor prognosis |
| 90–94 years | 1.214 | Exceeds 1.0 | Very low survival | Competing mortality |
| 95+ years | 1.632 | Exceeds 1.0 | Very low survival | Competing mortality |
| **MIR Summary Statistics:** | | | | |
| Minimum MIR | 0.123 | 20–24 years | — | Best prognosis age |
| Maximum MIR | 1.632 | 95+ years | — | Worst prognosis age |
| Mean MIR | 0.454 | All ages | — | Overall average |
| MIR at screening age (50–74) | 0.27–0.44 | Range | — | Target population |

***Note:*** *Log-polynomial regression (degree 4) was used for smoothing to ensure all fitted values remain positive. Adjusted R² values are reported to account for model complexity (5 parameters). GBD age-specific rates are outputs from DisMod-MR Bayesian meta-regression, not raw observations.****Abbreviations:*** *df, degrees of freedom; dRate/dAge, first derivative of rate with respect to age; MAE, mean absolute error; MIR, mortality-to-incidence ratio; RMSE, root mean square error; SS, sum of squares; UI, uncertainty interval.*
