# Supplementary material for: National Burden of Breast Cancer in Saudi Arabia, 1990–2023, With Forecasts to 2050: A Systematic Analysis for the Global Burden of Disease Study 2023
Source: Evidance Health Sci. Author manuscript; Available in PMC 2026 May 7. (PMC13148422; doi:10.65416/ehealthsci.2026.117757)
Supplement: Appendix — Supplementary Figure 1: Joinpoint Regression Analysis of Incidence and Mortality Trends. Supplementary Figure 2: Lee-Carter Model Mortality Forecast To 2050. Supplementary Figure 3: Bayesian Age-Period-Cohort Variance Decomposition. Supplementary Figure 4: Compression Versus Expansion of Morbidity Analysis. Table 1: Annual Time Series of Breast Cancer Burden In Saudi Arabia, 1990–2023. Supplementary Table 2: Sex-Specific Annual Time Series of Breast Cancer Burden In Saudi Arabia, 1990–2023. Supplementary Table 3: Annual Time Series of YLLs, YLDs, and Prevalence For Breast Cancer In Saudi Arabia, 1990–2023. Supplementary Table 4: Detailed Statistical Analysis and Sensitivity Assessment of Breast Cancer Trends In Saudi Arabia, 1990–2023. [file NIHMS2163534-supplement-Appendix.zip › Supplementary Table 7.docx]

**Supplementary Table 7:** Temporal Trend Decomposition and Age-Period-Cohort Analysis for Breast Cancer in Saudi Arabia, 1990–2023.

| **Measure/Parameter** | **1990–2000** | **2000–2010** | **2010–2023** | **1990–2023** |
| --- | --- | --- | --- | --- |
| **Period-Specific Changes in Absolute Burden:** | | | | |
| Incidence, n (Δ) | +658 | +1,711 | +1,218 | +3,587 |
| Incidence, n (% change) | +150.1% | +156.1% | +43.4% | +818.6% |
| Deaths, n (Δ) | +235 | +527 | +154 | +916 |
| Deaths, n (% change) | +100.8% | +112.4% | +15.5% | +392.7% |
| DALYs (Δ) | +9,678 | +19,676 | +4,288 | +33,643 |
| DALYs (% change) | +117.8% | +110.0% | +11.4% | +409.7% |
| **Annual Percent Change (95% CI):** | | | | |
| Incidence ASR | +3.36% | +6.61% | +1.60% | +3.96% |
| (95% CI) | (3.00 to 3.72) | (6.34 to 6.88) | (0.95 to 2.25) | (3.64 to 4.28) |
| Mortality ASR | +1.33% | +5.17% | +0.13% | +2.42% |
| (95% CI) | (0.96 to 1.70) | (4.81 to 5.53) | (−0.40 to 0.66) | (2.12 to 2.72) |
| DALY rate ASR | +2.17% | +4.59% | −0.23% | +2.05% |
| (95% CI) | (1.84 to 2.50) | (4.30 to 4.88) | (−0.96 to 0.51) | (1.71 to 2.38) |
| **ASR Values (per 100,000):** | 1990 | 2000 | 2010 | 2023 |
| Incidence ASR | 15.28 | 21.84 | 40.54 | 49.36 |
| (95% UI) | (10.70–21.31) | (17.22–26.75) | (32.43–50.97) | (36.19–66.12) |
| Mortality ASR | 9.39 | 10.99 | 17.72 | 17.86 |
| (95% UI) | (6.53–12.92) | (8.78–13.10) | (14.44–21.64) | (13.01–22.54) |
| DALY rate ASR | 267.60 | 338.68 | 515.28 | 494.92 |
| (95% UI) | (188.02–374.19) | (267.85–409.95) | (414.49–633.11) | (365.40–681.99) |
| **Lee-Carter Model:** | | | | |
| Variance explained | 99.3% | — | — | — |
| Age groups analyzed | 13 | — | — | 15–79 years |
| Time points | 8 | — | — | 1990–2023 |
| **Lee-Carter Age Effects:** | ax (mean log-rate) | bx (sensitivity) | — | — |
| 15–19 years | 0.025 | 0.054 | Lowest rate | — |
| 20–24 years | 0.677 | 0.068 | — | — |
| 25–29 years | 1.795 | 0.066 | — | — |
| 30–34 years | 2.596 | 0.061 | — | — |
| 35–39 years | 3.100 | 0.061 | — | — |
| 40–44 years | 3.687 | 0.070 | — | — |
| 45–49 years | 3.961 | 0.089 | High sensitivity | — |
| 50–54 years | 4.243 | 0.079 | — | — |
| 55–59 years | 4.261 | 0.080 | — | — |
| 60–64 years | 4.633 | 0.068 | — | — |
| 65–69 years | 4.569 | 0.085 | — | — |
| 70–74 years | 4.839 | 0.099 | — | — |
| 75–79 years | 4.802 | 0.121 | Highest sensitivity | — |
| **Lee-Carter Period Effects (kt):** | Year | kt value | Interpretation |  |
| Reference period | 1990 | −8.62 | Lowest | — |
| Early increase | 1995 | −5.90 | Rising | — |
| Continued rise | 2000 | −3.73 | Rising | — |
| Inflection point | 2005 | 0.00 | Baseline | — |
| Rapid increase | 2010 | +4.18 | High | — |
| Plateau | 2015 | +3.94 | Stable | — |
| Stable high | 2019 | +4.25 | High | — |
| Current period | 2023 | +5.88 | Highest | — |
| **Lee-Carter Cohort Effects:** | Birth cohort | Effect range | n observations | — |
| Early cohorts | 1918–1927 | +0.08 to −0.10 | 2–3 | — |
| Pre-war generation | 1928–1937 | −0.13 to −0.04 | 4–5 | — |
| War generation | 1938–1947 | +0.06 to 0.00 | 6 | — |
| Post-war generation | 1948–1957 | −0.05 to −0.03 | 6 | — |
| Oil boom era | 1958–1967 | +0.02 to +0.04 | 6 | — |
| Modernization era | 1968–1977 | +0.04 to +0.05 | 6 | — |
| Urbanization era | 1978–1987 | +0.04 to 0.00 | 4–5 | — |
| Recent cohorts | 1988–1993 | −0.08 to −0.07 | 2–3 | — |

***Abbreviations:*** *APC, annual percent change; ASR, age-standardized rate; ax, average log-rate by age (Lee-Carter parameter); bx, age-specific sensitivity to temporal change (Lee-Carter parameter); CI, confidence interval; DALY, disability-adjusted life year; kt, period-specific time index (Lee-Carter parameter); n, number; UI, uncertainty interval; Δ, absolute change.*
