# Supplementary material for: National Burden of Breast Cancer in Saudi Arabia, 1990–2023, With Forecasts to 2050: A Systematic Analysis for the Global Burden of Disease Study 2023
Source: Evidance Health Sci. Author manuscript; Available in PMC 2026 May 7. (PMC13148422; doi:10.65416/ehealthsci.2026.117757)
Supplement: Appendix — Supplementary Figure 1: Joinpoint Regression Analysis of Incidence and Mortality Trends. Supplementary Figure 2: Lee-Carter Model Mortality Forecast To 2050. Supplementary Figure 3: Bayesian Age-Period-Cohort Variance Decomposition. Supplementary Figure 4: Compression Versus Expansion of Morbidity Analysis. Table 1: Annual Time Series of Breast Cancer Burden In Saudi Arabia, 1990–2023. Supplementary Table 2: Sex-Specific Annual Time Series of Breast Cancer Burden In Saudi Arabia, 1990–2023. Supplementary Table 3: Annual Time Series of YLLs, YLDs, and Prevalence For Breast Cancer In Saudi Arabia, 1990–2023. Supplementary Table 4: Detailed Statistical Analysis and Sensitivity Assessment of Breast Cancer Trends In Saudi Arabia, 1990–2023. [file NIHMS2163534-supplement-Appendix.zip › Supplementary Table 8.docx]

**Supplementary Table 8:** Infrastructure, Hierarchy Validation, and Life Table Analysis for Breast Cancer in Saudi Arabia.

| **Parameter** | **Classification** | **Value** | **95% UI** |
| --- | --- | --- | --- |
| **Cause Hierarchy:** | | | |
| Level 1 | All causes | — | — |
| Level 2 | Non-communicable diseases | — | — |
| Level 3 | Neoplasms | — | — |
| Level 4 | Breast cancer | — | ICD-10: C50 |
| **Disability Weights:** | | | |
| Diagnosis/treatment phase | Primary treatment | 0.288 | 0.193–0.399 |
| Controlled phase | Remission | 0.049 | 0.031–0.072 |
| Metastatic phase | Advanced disease | 0.451 | 0.307–0.600 |
| Terminal phase | End of life | 0.540 | 0.377–0.687 |
| **Associated Risk Factors:** | | | |
| High body-mass index | Metabolic | BMI ≥25 kg/m² | — |
| Alcohol use | Behavioral | Any consumption | — |
| Low physical activity | Behavioral | Below guidelines | — |
| High fasting plasma glucose | Metabolic | Elevated FPG | — |
| **Remaining Life Expectancy at Death (years), Females 2023:** | | | |
| 15–19 years | YLLs: 169 | 72.6 | — |
| 20–24 years | YLLs: 265 | 67.6 | — |
| 25–29 years | YLLs: 939 | 62.6 | — |
| 30–34 years | YLLs: 2,889 | 57.7 | — |
| 35–39 years | YLLs: 4,495 | 52.8 | — |
| 40–44 years | YLLs: 5,468 | 47.9 | — |
| 45–49 years | YLLs: 5,159 | 43.0 | — |
| 50–54 years | YLLs: 4,985 | 38.2 | — |
| 55–59 years | YLLs: 4,392 | 33.5 | — |
| 60–64 years | YLLs: 4,156 | 28.9 | — |
| 65–69 years | YLLs: 2,511 | 24.3 | — |
| 70–74 years | YLLs: 1,668 | 20.0 | — |
| 75–79 years | YLLs: 1,180 | 16.0 | — |
| 80–84 years | YLLs: 791 | 12.6 | — |
| 85–89 years | YLLs: 206 | 10.0 | — |
| 90–94 years | YLLs: 62 | 8.6 | — |
| 95+ years | YLLs: 13 | 8.2 | — |
| **Deaths by Age Group, n (95% UI), Females 2023:** | | | |
| 15–19 years | — | 2.3 | 1.4–3.7 |
| 20–24 years | — | 3.9 | 2.3–5.8 |
| 25–29 years | — | 15.0 | 9.5–21.5 |
| 30–34 years | — | 50.1 | 32.0–82.3 |
| 35–39 years | — | 85.1 | 52.4–147.7 |
| 40–44 years | — | 114.2 | 76.1–203.5 |
| 45–49 years | — | 119.9 | 79.7–192.2 |
| 50–54 years | — | 130.5 | 84.8–217.1 |
| 55–59 years | — | 131.3 | 85.7–186.0 |
| 60–64 years | Peak mortality | 144.0 | 94.9–206.7 |
| 65–69 years | — | 103.2 | 69.2–146.4 |
| 70–74 years | — | 83.3 | 55.1–121.8 |
| 75–79 years | — | 73.9 | 44.7–106.6 |
| 80+ years | Combined | 92.4 | 53.7–137.3 |
| Total | All ages | 1,149 | 852–1,625 |
| **Burden Estimates, Females 2023:** | | | |
| Incidence, n | New cases | 4,025 | 2,886–5,836 |
| Prevalence, n | Living with disease | 34,708 | 27,483–48,229 |
| Deaths, n | Mortality | 1,149 | 852–1,625 |
| YLLs | Premature death | 39,347 | 29,267–59,319 |
| YLDs | Disability | 2,508 | 1,625–4,009 |
| DALYs | Total burden | 41,855 | 31,446–62,282 |
| Incidence ASR per 100,000 | Age-standardized | 49.36 | 36.19–66.12 |
| Mortality ASR per 100,000 | Age-standardized | 17.86 | 13.01–22.54 |
| DALY rate ASR per 100,000 | Age-standardized | 494.92 | 365.40–681.99 |
| Mortality-to-incidence ratio | MIR | 0.285 | — |
| **Temporal Trends, Females 1990–2023:** | | | |
| Incidence, n | 438 → 4,025 | +3,587 | +818.6% |
| Prevalence, n | 4,406 → 34,708 | +30,302 | +687.7% |
| Deaths, n | 233 → 1,149 | +916 | +392.7% |
| DALYs | 8,212 → 41,855 | +33,643 | +409.7% |
| Incidence ASR per 100,000 | 15.28 → 49.36 | +34.08 | +223.0% |
| Mortality ASR per 100,000 | 9.39 → 17.86 | +8.47 | +90.2% |

***Abbreviations:*** *ASR, age-standardized rate; BMI, body-mass index; DALY, disability-adjusted life year; FPG, fasting plasma glucose; GBD, Global Burden of Disease; ICD, International Classification of Diseases; MIR, mortality-to-incidence ratio; n, number; SDI, Socio-demographic Index; UI, uncertainty interval; YLD, years lived with disability; YLL, years of life lost.*
